# First report of an Onchocercidae worm infecting *Psychodopygus carrerai carrerai* sandfly, a putative vector of *Leishmania braziliensis* in the Amazon

**DOI:** 10.1038/s41598-020-72065-9

**Published:** 2020-09-17

**Authors:** Andreia Fernandes Brilhante, Alessandra Lima de Albuquerque, Abraham Cézar de Brito Rocha, Constância Flávia Junqueira Ayres, Marcelo Henrique Santos Paiva, Márcia Moreira de Ávila, Cristiane de Oliveira Cardoso, Isabel L. Mauricio, Eunice Aparecida Bianchi Galati

**Affiliations:** 1 Public Health School, Epidemiology Department, University of São Paulo, São Paulo, Brazil; 2 Entomology Department, Aggeu Magalhães Institute, Oswaldo Cruz Foundation, Recife, Pernambuco Brazil; 3 National Center of Lymphatic Filariasis, Parasitology Department, Aggeu Magalhães, Institute, Oswaldo Cruz Foundation, Recife, Pernambuco Brazil; 4 Laboratory of Hospital Otávio de Freitas – Department of Health of the State of Pernambuco, Recife, Brazil; 5 Federal University of Pernambuco, Núcleo de Ciências da Vida - Centro Acadêmico do Agreste, Caruaru, Pernambuco Brazil; 6 Federal Institute of Acre, Rio Branco, Acre Brazil; 7 Center for Health and Sport Sciences, Federal University of Acre, Rio Branco, Acre Brazil; 8 Global Health and Tropical Medicine (GHTM), Instituto de Higiene e Medicina Tropical, Universidade Nova de Lisboa (UNL), Lisboa, Portugal

**Keywords:** Entomology, Parasitic infection

## Abstract

Sandflies are insects of public health interest due to their role as vectors of parasites of the genus *Leishmania*, as well as other pathogens. *Psychodopygus carrerai carrerai* is considered an important sylvatic vector of *Leishmania* (*Viannia*) *braziliensis* in Amazonia. In this study, sandflies were collected in a forested area in the Xapuri municipality, in the State of Acre (Northern Brazil). Two *Ps. carrerai carrerai* females were found parasitized with a larval form of a filarial worm, one in the labium of the proboscis, the other after the head was squashed, suggesting they were infective larvae. Sandflies were identified through morphological characters as well as amplification and sequencing of the cytochrome oxidase gene (COI). This was the first sequence obtained for *Ps. carrerai carrerai* for this marker. The obtained nematodes were also characterized through direct sequencing of a fragment of COI and 12S genes, both mitochondrial, and ITS1, a nuclear marker. Phylogenetic analyses revealed that the filarial nematodes belong to a species without sequences for these markers in the database, part of family Onchocercidade and closely related to genus *Onchocerca* (12S tree). Although sandfly infection with nematodes including members of the Onchocercidae has been reported in the Old World, this is the first report of sandfly infection by a member of the Onchocercidae family in the New World, to the best of our knowledge. Considering that the phylogenetic relationships and location in the insect, it can be expected that this is a parasite of mammals and the transmission cycle should be clarified.

## Introduction

Phlebotomine sandflies are dipterans of medical significance due to their role in the transmission of the aetiological agents of leishmaniasis and bartonellosis, as well as of arboviruses^1^. The greatest sandfly diversity in the Americas is found in the Amazon region^2^. An important sylvatic vector of American cutaneous leishmaniasis in the Bolivian Amazon rainforest^3^ is the sandfly *Psychodopygus carrerai carrerai* (Barretto, 1946). This species is anthropophilic and associated with primary forest environments, where it has been found to be naturally infected by *Leishmania* (*Viannia*) *braziliensis.* In addition to *Leishmania*, other protozoa, such as trypanosomatids from the genera *Endotrypanum* and *Trypanosoma*^4^ and from the phyla Apicomplexa, *Ascogregarina* and *Psychodiella*^5,6^ have been reported to infect sandflies. Infections by nematodes have rarely been reported^7^. Nonetheless, *Madathamugadia wanjii* (fam. Onchocercidae) in *Phlebotomus duboscq*^8^, *Didilia* sp., *Didilia ooglypta,* members of the Tylenchoidea superfamily, in *Phlebotomus papatasi* and *Phlebotomus sergenti*^9–12^ and members of the Steinernematidae family in *Phlebotomus tobbi*^13^ have been reported in the Old World. In South America, natural infections by *Anandarema phlebotophaga* (Allantonematidae: Tylenchida)^14^ and other nematodes, tentatively assigned to family Steinernematidae^15^, have been observed in colonies of *Lutzomyia longipalpis*. Additionally, in Argentina, natural populations of *Pintomyia fischeri* have been found infected by Tylenchid nematodes^16^. Unfortunately, few DNA sequences are available from these nematodes found in sandflies, which means that phylogenetic comparison between isolates and reports is very difficult.

Family Onchocercidae includes worms of the genus *Onchocerca*, which comprises 28 species. In humans, *Onchocerca volvulus* causes onchocerciasis^17^, also known as river blindness, while *O. lupi* is a mainly zoonotic parasite of dogs^18,19^. Other species such as *O. gutturosa*, *O. gibsoni*, *O. cervicalis* and *O. ochengi* are of veterinary importance in ruminants, horses and dogs^20^. Species of this genus are usually transmitted by simulid species (blackflies). Other members of this family include the agents of lymphatic filariasis (*Wuchereria bancrofti* and species of the genus *Brugia*) and mainly zoonotic parasites, of the genera *Dirofilaria*, *Mansonella*, *Acanthocheilonema*, among others^21^.

In a recent survey of American cutaneous leishmaniasis (ACL) and sandflies vectors performed in Xapuri, two *Ps. carrerai carrerai* females were found parasitized with a filarial larval form, whose infection is here reported. Xapuri is a municipality located in Acre State, Brazil, and it is an area of high prevalence of ACL with a high diversity of sandflies species^22,23^. There is no record of parasitic diseases caused by filarids in the municipality of Xapuri. However, in the surrounding municipalities, the occurrences of *Mansonella ozzardi* and *Onchocerca volvulus* infecting indigenous and riverside populations have been reported^24–26^, and recently, *Wuchereria bancrofti*, the filarial worm of lymphatic filariasis, transmitted by mosquitoes, has been identified in immigrants from Haiti^27^.

## Results

**Figure 1:**
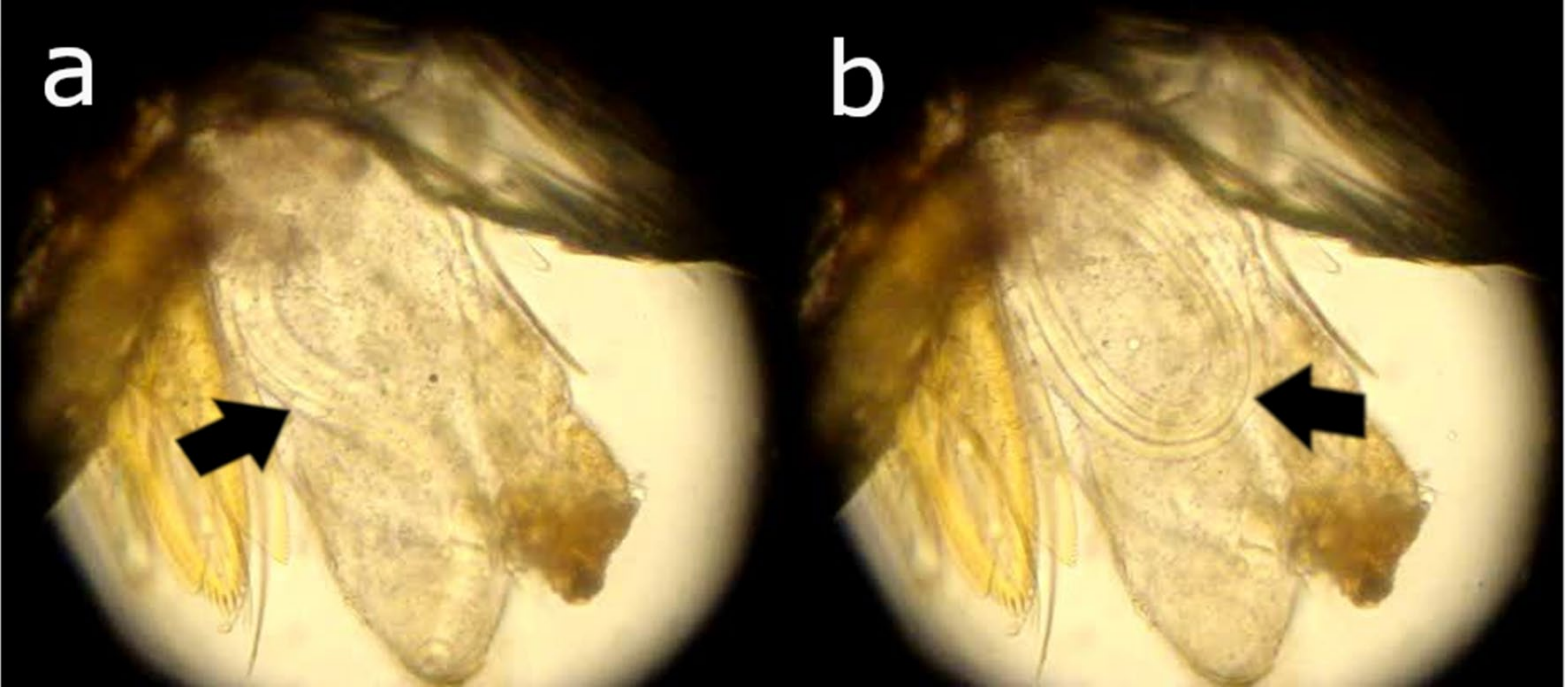
Microscopy photograph of the head of a specimen *Ps. carrerai carrerai* female with the presence of an Onchocercidae worm larva. (**A**) Larvae in a linear conformation, and (**B**) Larvae in a round conformation.

A total of 2,643 specimens of *Ps. carrerai carrerai* (2,233 females and 410 males) were collected, of which 139 females were dissected for trypanosomatids detection. Two females were found to be parasitized by a nematode larva. One of these nematodes was found on July 31, 2015, in the labium of the proboscis of the sandfly, while the other was only observed after the insect head was squashed on March 10, 2016 (Fig. 1 and Supplementary video). Ecological analyses of the phlebotomine fauna have been published elsewhere^23^.

The two sandflies samples were unambiguously identified as *Ps. carrerai carrerai*, based on morphological criteria. The PCR amplification of COI, using primers LCO1490 and HCO2198, from these two sandflies resulted in amplicons with the expected molecular weight (658 bp). The obtained sequences (GenBank MG029462 and MG029463) were identical for both specimens and had, upon a BLAST search, 90% nucleic acid identity with at least three different species of sandflies: *Ps. hirsutus hirsutus*, *Lutzomyia carrerai thula*, and *Psathyromyia pascalei* (Table 1).

**Table 1:** Sequencing results showing the BLAST (Megablast option, except where indicated) homology of sandfly and filarial parasite sequences obtained from Xapuri—Rio Branco in relation to sequences found in GenBank.

Molecular marker	Query sequence	Match	Identity	GenBank accession
COI	Sandfly (658 bp) MG029462–MG029463	*Psychodopygus (syn. Lutzomyia) hirsutus*	595/658 (90%)	KP112991.1
		*Psychodopygus (syn. Lutzomyia) thula*	592/657 (90%)	KC921238.1
		*Psathyromyia (syn. Lutzomyia) pascalei*	589/658 (90%)	KP112959.1
	Parasite (649 bp) MG029460–MG029461	*Onchocerca gibsoni*	587/648 (91%)	AJ271616.1
		*Onchocerca volvulus*	587/649 (90%)	KC167355.1
		*Onchocerca ochengi*	586/649 (90%)	KX181290.2
12S	Parasite (465 bp) MH049488	*Onchocerca takaokai*	439/471/ (93%)	AB972364.1
		*Onchocerca flexuosa*	429/465 (92%)	AP017692.1
		*Onchocerca sp type A*	431/470 (92%)	AB518879.1
		*Onchocerca cervipedis*	422/461 (92%)	JX075208.1
ITS	Parasite (376 bp) MH049489	*Onchocerca fasciata*	156/174 (90%)	JQ316671.1
		*Onchocerca volvulus*	145/161 (90%)	EU272179.1
		*Onchocerca* sp. 1 WS-2017	292/373 (78%) BLASTn	MG192126.1
		*Onchocerca dewittei japonica*	284/360 (79%) BLASTn	MG192134.1

The COI sequences (649 bp; GenBank MG029460 and MG029461) for both parasite samples, which were also identical, and the 12S (465 bp; MH049488) and ITS-1 (376 bp; MH049489) sequences derived from filarial nematoids had maximum BLASTn identity results of 90–93% with species of family Onchocercidae, in particular species of the genus *Onchocerca* (Table 1). The alignments of the sequences used for phylogenetic analyses from COI, 12S and ITS1 genes are presented in Supplementary Informations 1, 2 and Supplementary Fig. S2, respectively.

**Figure 2:**
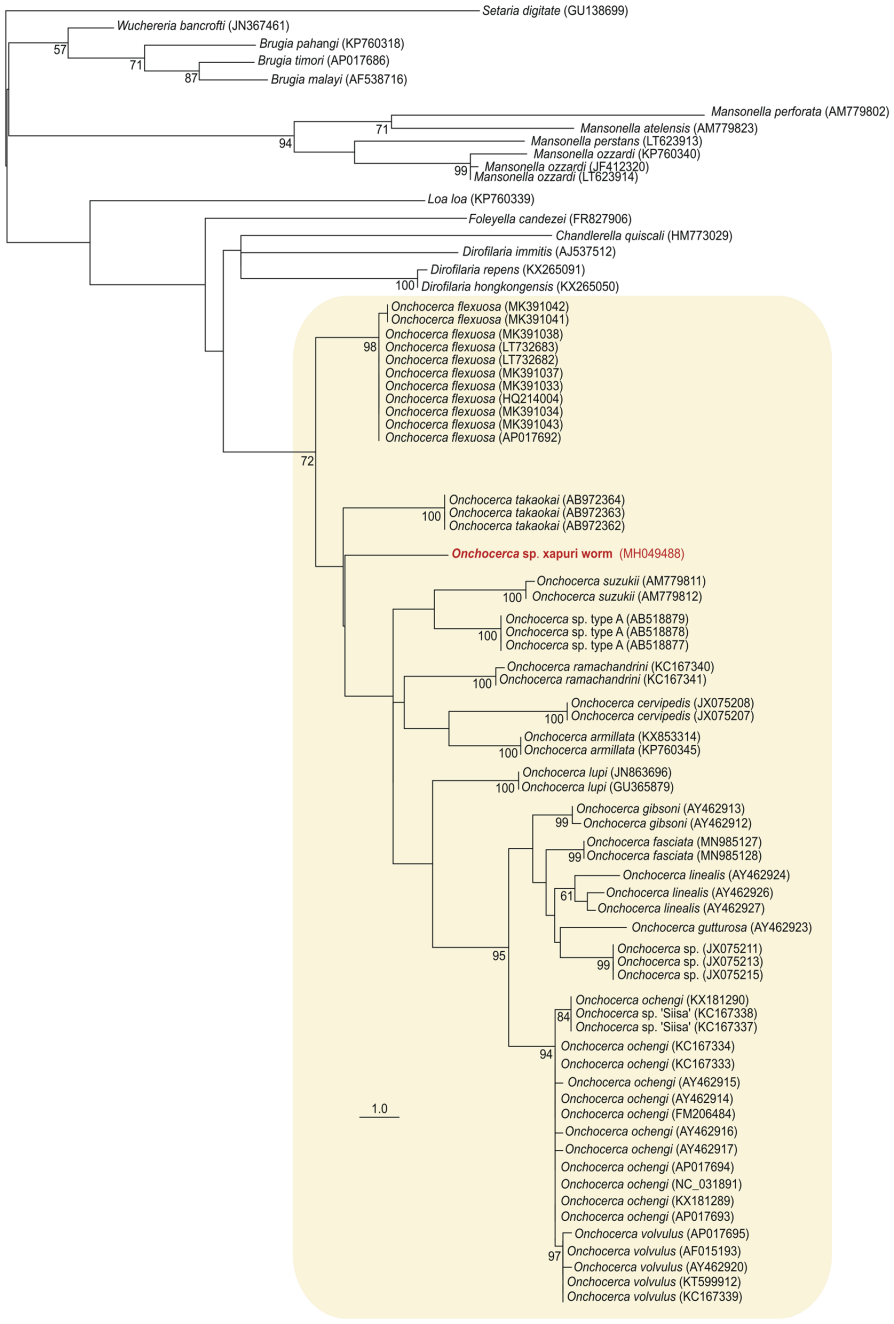
Phylogenetic tree based on the 12S gene of the new *Onchocerca* sp. Xapuri worm in Onchocercidae family. Phylogenetic tree inferred by maximum likelihood (403 characters, – Ln = 2,743.160330) of 12S sequences from 58 isolates belonging to the genus *Onchocerca* (yellow box) and 17 sequences that representing other genera/species from Onchocercidae family. Numbers at nodes are support values derived from 1,000 replicates in maximum likelihood analyses. Codes within parenthesis are GenBank accession numbers.

**Figure 3:**
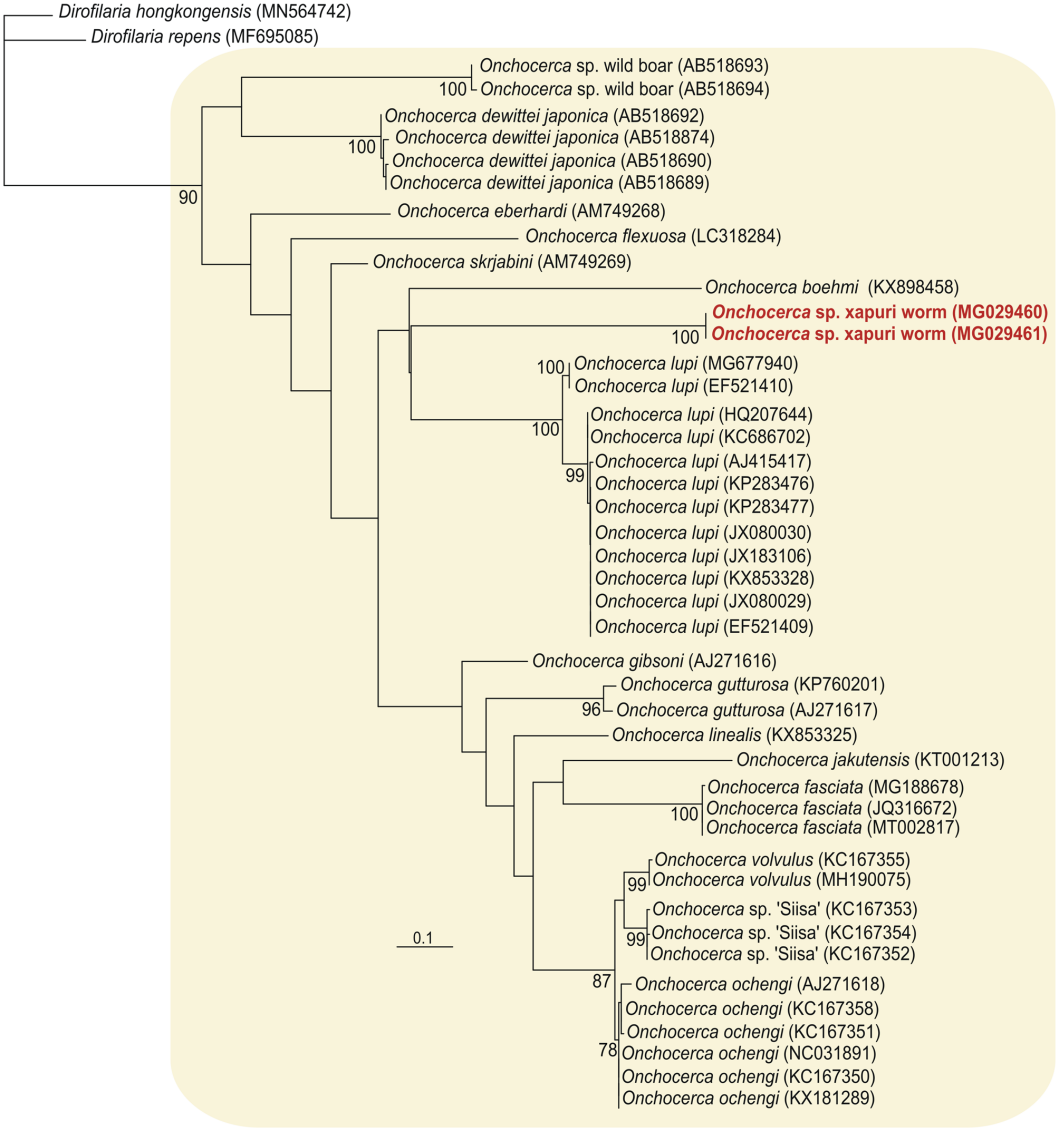
Phylogenetic tree based on the COI gene of the new Onchocerca sp. Xapuri worm. Phylogenetic tree inferred by maximum likelihood (649 characters, – Ln = 3,152.161339) of COI sequences from 43 isolates belonging to the genus *Onchocerca* (yellow box) and 2 sequences of *Dirofilaria* spp. used as outgroup. Numbers at nodes are support values derived from 1,000 replicates in maximum likelihood analyses. Codes within parenthesis are GenBank accession numbers.

The alignment of the 12S gene was performed using different species from the Onchocercidae family acoording to Crainey et al*.*^28^ and using *Setaria diditate* as an outgroup. For the COI gene, the alignment was made with different *Onchocerca* and *Dirofilaria* species (external group). Phylogenetic analyses with mitochondrial genes (12S and COI) clustered the new sequence from the Xapuri worm in the clade of *Onchocerca* species with 72% and 99% bootstrap, respectively. This clade was more closely related to the genus *Dirofilaria* (Figs. 2 and 3). In the network tree from all ITS-1 (Supplementary Fig. S1) available in the Genbank sequences from Family Onchocercidae and related species as outgroup, the Xapuri worm sequences clustered with Family Onchocercidae (bootstrap support > 80%). However with the analysis of this gene it was not possible to identify which genus or species it belongs to.

## Discussion

During a survey of *Leishmania* vectors in forest areas of Xapuri, Acre state, Brazilian Amazon, filarial worms were detected in phlebotomine sandflies. The vector species was unambiguously identified morphologically as *Ps. carrerai carrerai*, based on spermatheca characteristics, thorax coloration and labrum length. Barcoding analysis using COI sequences confirmed that the samples were more closely related to *Ps. hirsutus hirsutus*, but public databases lack *Ps. carrerai carrerai* sequences. As such, this was the first COI sequence deposited in GenBank for *Ps. carrerai carrerai*. COI DNA barcoding will become a more accurate tool to identify species of sandflies in Brazil as more sequences are added to the databases, as shown by Pinto et al*.*^29^. Therefore, the new sequences generated in this study for *Ps. carrerai carrerai* will be important for future identification based on molecular data.

It was not possible to identify morphologically the two samples of filarid worms because of field work conditions and lack of a nematode specialist during sandfly collection. Indeed, previous studies have demonstrated similarities among *Onchocerca* species, for example: *O. gibsoni* and *O. volvulus* have highly similar cuticle morphologies and chromosomal data^17,30^. According to Gasser^31^, some of the aspects that are taken into account to identify parasites are morphological features, the host they infect, and their geographical origin. However, these criteria are often insufficient for specific identification and there are limitations of traditional approaches as microscopic analysis. Molecular approaches have provided powerful alternative tools to overcome these complications.

The BLAST searches of three genetic markers (one nuclear—ITS-1—and two mitochondrial—COI and 12S) and phylogenetic trees based on mitochondrial genes sequences support the classification of Xapuri worm in the *Onchocerca* genus (Table 1, Figs. 2 and 3). However, the phylogenetic relationship of this isolate with the other species of the genus *Onchocerca* varied according to the analyzed gene. In the 12S phylogenetic tree, Xapuri worm it was closer to *O. takaokai*. But in the COI tree it was more related to *O. lupi*. Although there are many sequences of *Onchocerca* spp. deposited in the GenBank database, many species do not have sequences of both genes (12S and COI). Further studies are needed to verify whether this parasite is more related to a species of *Onchocerca* already described, or if it is a new species of the genus. For this reason it was denominated as *Onchocerca* sp. Xapuri worm.

To the best of our knowledge, this is the first record of sandflies carrying filarial worms of the family Onchocercidae in the Americas, and only the second in the world, the other being of *Madathamugadia wanjii* (fam. Onchocercidae) in *Phlebotomus duboscq*^8^. The genus *Onchocerca* is common in South America, and in Brazil, the most prevalent species of this genus are *O. gutturosa* in cattle and *O. cervicalis* in horses^20,32^, both with veterinary importance. In the Amazon region, human onchocerciasis caused by *O. volvulus* was described in the 1960s^33^. However, other filarids of similar medical and clinical importance, such as *Mansonella ozzardi* and *Ma. perstans,* have also been found in sympatry in this region^24,25,34^. Additionally, other atypical filarids are in circulation and result in unknown clinical aspects^35,36^. To distinguish sympatric filarial species from the Amazon Region, Tang et al*.*^34^ proposed a system based on amplification of the internal transcribed spacer (ITS-1). In this analysis, the size of the yielded amplicons varies for each Amazonian filariae species, *Ma. perstans* (312 bp), *Ma. ozzardi* (305 bp) and *O. volvulus* (344 bp). The ITS-1 fragment of the parasite here investigated by the same system had a different band size, 416 bp (including primers), which also differs from the other parasites as *Loa loa* (344 bp), *Wuchereria bancrofti* (301 bp) or *Dirofilaria immitis* (276 bp). ITS-1primers were designed to target a highly conserved genomic region among filarial species and represents an important region for gene splicing.

Another important point to be considered in the phylogenetic analyzes of these filarids is the occurrence of mtDNA pseudogenes, commonly known as Numts, something common found in *M. ozzardi* COI sequences, as shown by the study by Crainey et al*.*^28^. According to the authors, the 12S gene seems to be more reliable for diagnosis and phylogenetic studies of this parasite. They also emphasized of the need for a better screening of these mitochondrial sequences of cryptic pseudogenes before being deposited in public domains. Our all COI sequences (649 bp) do not contain indels or stop codons, and there is no evidence of being considered a pseudogene.The location where both larvae were found (head) and the position in the labrum, as well as the very active and oscillatory movement of the larva found in *Ps. carrerai carrerai* in the field are characteristic of infective L3^37^ (Supplementary Video). However, it cannot be affirmed with certainty that the larva recovered was L3, given that it was not possible to perform morphological studies. The highly anthropophilic behaviour of this phlebotomine sandfly suggests that humans may be exposed to such nematodes.

*Psychodopygus carrerai carrerai* was collected in Xapuri, in a primary forest area in the presence of wild animals that included rodents, marsupials, felids and others. In the surrounding region, it was possible to find rural properties with limited cattle, horse and sheep breeding, which suggests that sandflies could be feeding on these animals, which could, therefore, possibly be hosts of the Onchocerdidae nematode described here. However, further investigations are necessary to determine if this is an insect or a zoonotic parasite and its possible host. More studies are also needed to characterize the filarids in circulation in this region, as well as to elucidate the vectorial capacity and competence of sandflies to transmit these parasites.

## Methods

### Study area and sandfly sampling

**Figure 4:**
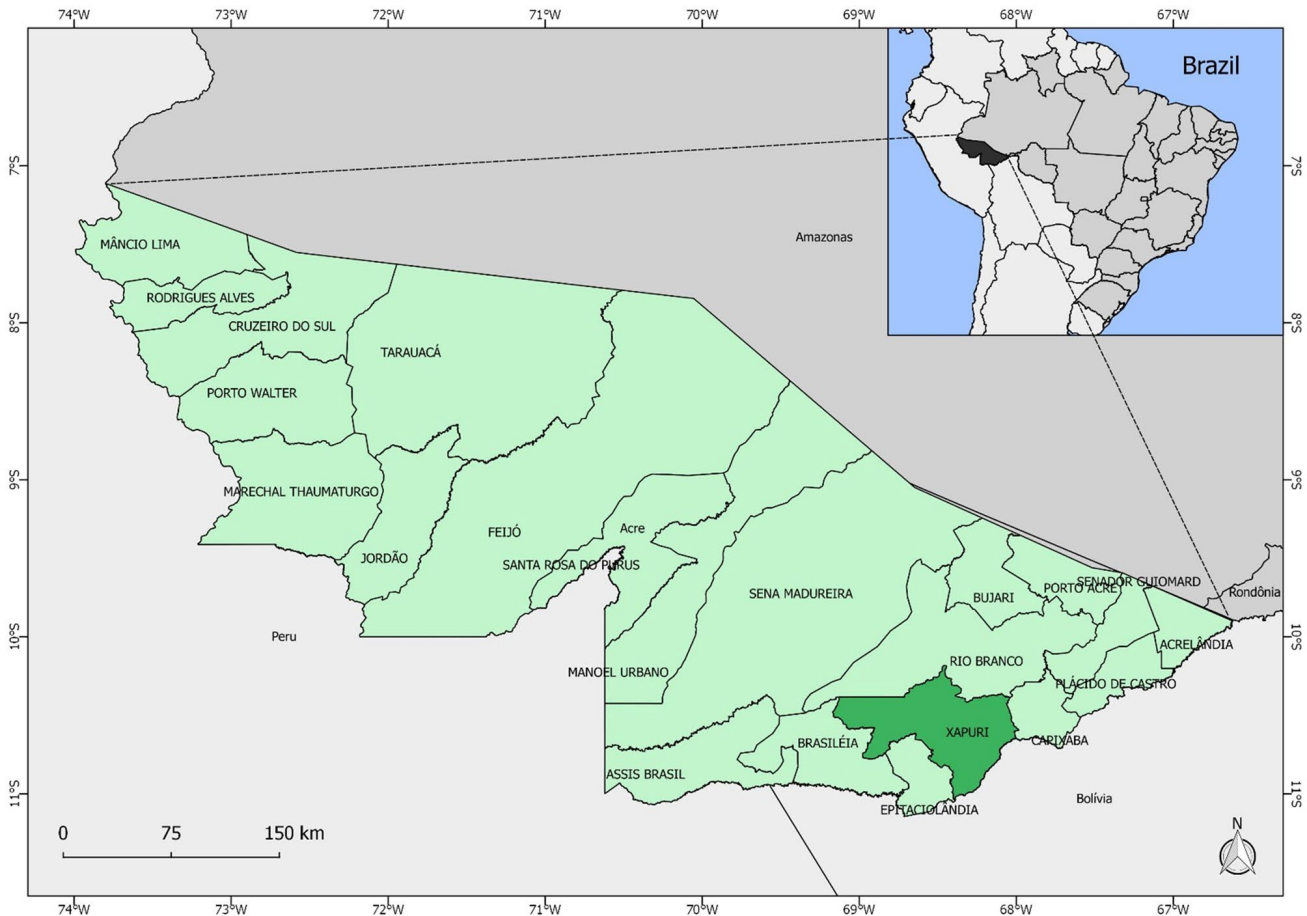
Map of Brazil highlighting the state of Acre and the Xapuri municipality.

Sandflies collections were undertaken in a forested area of the Xapuri municipality, which is approximately 175 km from Rio Branco, the capital of Acre state, Northern Brazil. Xapuri is situated in the Vale do Acre mesoregion and is bordered by the municipalities of Rio Branco, Brasiléia, Epitaciolândia and Capixaba, as well as the Amazonian border with Bolivia (Fig. 4). The local economy is based on rubber extraction and Brazil nuts^38^.

Two modified Shannon traps in black and white colours illuminated by LED lights (light emitting diodes—2 W) were installed in a primary forest area in which ecological and environmental tourism activities, such as hiking and tree climbing, are offered. The insects were collected during periods of varying length once a month between August 2013 and July 2015 and in March 2016.

Sandflies were maintained in separate vials for an interval of two hours and then taken to the field laboratory. Female insects were immobilized with ethyl acetate and dissected under a stereomicroscope on slides in a drop of sterile saline solution for exposure of the digestive tract and genitalia. Then, slides were covered with a coverslip and observed under the microscope to investigate the presence of flagellates in their guts. The guts were searched for natural infection by tripanosomatids and spermathecae for the identification of the sandfly species at a 400 X magnification. The identification of the insects collected in this study was performed following Galati’s identification keys^39^.

The insects with unidentified parasite in the proboscides were filmed and photographed, stored in absolute ethanol for molecular analysis, and then sent to the Oswaldo Cruz Foundation (FIOCRUZ) in Recife, Pernambuco, Brazil.

### Molecular identification of the species

#### DNA extraction and amplification

Before the DNA extraction, the two insects selected in this study were differently processed: from one, the unidentified parasite was separated from the insect, while the other was processed along with the parasite. Parasites and insects were submitted to individual DNA extraction, following protocol described in Ayres et al.^40^. Four different PCR reactions were performed with the same template, i.e., PCR I, which consisted of cytochrome oxidase subunit I (COI) using primers LCO1490 and HCO2198^41^ that targeted the insect (710 bp product size), and PCR II, which used a different set of primers (COIintF and COIintR), targeting a 689 bp DNA fragment of the parasite^42^. The PCR III and IV amplified only filarial DNA using respectively, primers FIL-2F and FIL-2R for 416 bp fragment of first internal transcribed spacer 1 (ITS-1)^34^ and primers 12SF and 12SdegR for 504 bp of 12S rDNA^43^. PCR reactions were performed in a 25-µL final volume containing the following: 1 × high-fidelity PCR buffer, 2 mM MgSO_4_, 0.5 U Platinum® *Taq* DNA Polymerase High-Fidelity (Invitrogen™), 200 µM dNTP, 0.4 µM of each primer, and 6.5 ng of template DNA. The PCR I program consisted of the following: 94 °C for 3 min; 35 cycles at 94 °C for 45 s, 55 °C for 1 min, 72 °C for 45 s; and one final extension step at 72 °C for 10 min. The program for the PCR II consisted of the following: 94 °C for 3 min; 40 cycles at 94 °C for 45 s, 52 °C for 45 s, and 72 °C for 90 s; and a final extension step at 72 °C for 10 min. PCR III and IV the program consisted of the following: 94 °C for 3 min; 35 cycles at 94 °C for 45 s, 50 °C for 45 s, and 72 °C for 45 s; and a final extension step at 72 °C for 5 min. The PCR products were analysed in a 2.5% agarose gel stained with ethidium bromide and visualized under UV light.

#### Sequencing and sequence analysis

PCR products were excised from the gel and purified using the *GFX™ PCR DNA and Gel Band Purification* Kit (GE Healthcare). Amplicons were sequenced in both directions using the PCR primers described above. Sequencing reactions were performed on an ABI Prism 3500xL Genetic Analyzer (Applied Biosystems). Quality assessment, edition, assembly and multiple alignments of data derived from sequencing were performed with CodonCode Aligner (v.3.7.1) and BioEdit/ClustalW^44^. The BLAST tool (www.ncbi.nlm.nih.gov/BLAST) was used to confirm the sequence identities by comparing our sequences to those deposited in GenBank database.

#### Phylogenetic analysis

Up to 500 homologous sequences were obtained from a BLAST search for COI, ITS-1 and 12S parasite sequences and aligned in BioEdit 7.2.5^44^. Regions of unreliable alignment were removed from ITS-1 alignments, as were sequences that were too short for all regions. Phylogenetic trees for 12S and COI genes were inferred by maximum likelihood (ML) method. The ML analyses were performed using RAxML v.7.0^45^ using the GTRGAMMA model, gamma shape parameter and proportion of invariable sites. Model parameters were estimated in RAxML over the duration of tree search. Nodal supports were estimated with 1,000 replicates in RAxML using the rapid bootstrapping algorithm. The network genealogy for ITS1 was perfomed by SplitsTree4 using the NeighborNet method^46^.

## Supplementary information

Supplementary Video.

Supplementary Information.

## Data Availability

All data generated or analyzed during this study are included in this published article.
