# Supplementary material for: First report of an Onchocercidae worm infecting Psychodopygus carrerai carrerai sandfly, a putative vector of Leishmania braziliensis in the Amazon
Source: Sci Rep. 2020 Sep 17;10:15246. doi: 10.1038/s41598-020-72065-9 (PMC7498610; doi:10.1038/s41598-020-72065-9)
Supplement: Supplementary file 2 — Supplementary Information. [file 41598_2020_72065_MOESM2_ESM.pdf]

Supplementary information

First report of an Onchocercidae worm infecting *Psychodopygus carrerai carrerai* sandfly, a putative vector of *Leishmania braziliensis* in the Amazon

Andreia Fernandes Brilhante[1,7], Alessandra Lima de Albuquerque[2], Abraham Cézar de Brito Rocha[3,4], Constância Flávia Junqueira Ayres[2*], Marcelo Henrique Santos Paiva[2,5], Márcia Moreira de Ávila[6], Cristiane de Oliveira Cardoso[7], Isabel L. Mauricio[8], Eunice Aparecida Bianchi Galati[1].

[1] Public Health School, Epidemiology Department, University of São Paulo, São Paulo, Brazil. Email: brilhanteaf@usp.br ; egalati@usp.br

[2] Entomology Department, Aggeu Magalhães Institute, Oswaldo Cruz Foundation, Recife, Pernambuco, Brazil. Email: ale@cpqam.fiocruz.br ; tans@cpqam.fiocruz.br ; marceloh@cpqam.fiocruz.br

[3] National Center of Lymphatic Filariasis, Parasitology Department, Aggeu Magalhães, Institute, Oswaldo Cruz Foundation, Recife, Pernambuco, Brazil. Email: rocha@cpqam.fiocruz.br

[4] Laboratory of Hospital Otávio de Freitas – Department of Health of the State of Pernambuco

[5] Federal University of Pernambuco, Núcleo de Ciências da Vida - Centro Acadêmico do Agreste, Caruaru, Pernambuco, Brazil.

[6] Federal Institute of Acre, Rio Branco, Acre, Brazil. Email: marcia.avila@ifac.edu.br

[7] Center for Health and Sport Sciences, Federal University of Acre, Rio Branco, Acre, Brazil. Email: crisdeocardoso@gmail.com

[8] Global Health and Tropical Medicine (GHTM), Instituto de Higiene e Medicina Tropical, Universidade Nova de Lisboa (UNL), Lisboa, Portugal. Email: isabel.mauricio@ihmt.unl.pt

*Correspondence and requests for materials should be addressed to Constância Flávia Junqueira Ayres (email: tans@cpqam.fiocruz.br)

Supplementary Video. Video of a female specimen of *Ps. carrerai carrerai* parasitized with an Onchocercidae worm larvae form.

List of Supplementary Figures:

Figure S1 - A network generated by the NeighborNet algorithm in the program SplitsTree, derived from Kimura-2-parameter distances of ITS sequences. The worm sequence from Xapuri and indistinguishable sequences are highlighted in red on the network.

Figure S2 - Alignment of the most similar sequences to Xapuri ITS obtained through a BLAST search and used to produce **Figure S1**. Sequences were aligned through Clustal X in BioEdit42, using default settings, and with gaps and regions of uncertain alignment removed manually. If more than one sample corresponded to each sequence it is indicated with a superscript number, as follows. **1** Xapuri ITS; AF228576.1 O. volvulus; KR080190.1 *Loa loa*; JQ327149.1 *Brugia malayi*; JQ316671.1 *Onchocerca fasciata*; EU419347.1 *Brugia malayi*; EU272178.1 *Wuchereria bancrofti*. 2 HG005138.1 *Acanthocheilonema spirocauda*; AF217801.2 *Dipetalonema reconditum*. 3 LN869521.1 Onchocercidae sp.; LN869520.1 Onchocercidae sp.; KY586137.1 Onchocercidae sp.. 4 LT623912.1 *Mansonella ozzardi*; LT623911.1 *Mansonella perstans*; KR080186.1 *Mansonella* sp.; KR080185.1 *Mansonella* sp.. 5 KX944557.1 *Malayfilaria sofiani*; KU757075.1 *Setaria yehi*. 6 EU087700.1 *Dirofilaria immitis*; AY621481.1 *Dirofilaria repens*.

Supplementary information 1. Alignment 12S – Nucleotide alignment of the 12S gene used in the phylogenetic analysis represented in Fig. 2.

Supplementary information 2. Alignment COI – Nucleotide alignment of the COI gene used in the phylogenetic analysis represented in Fig 3.

Figure S1

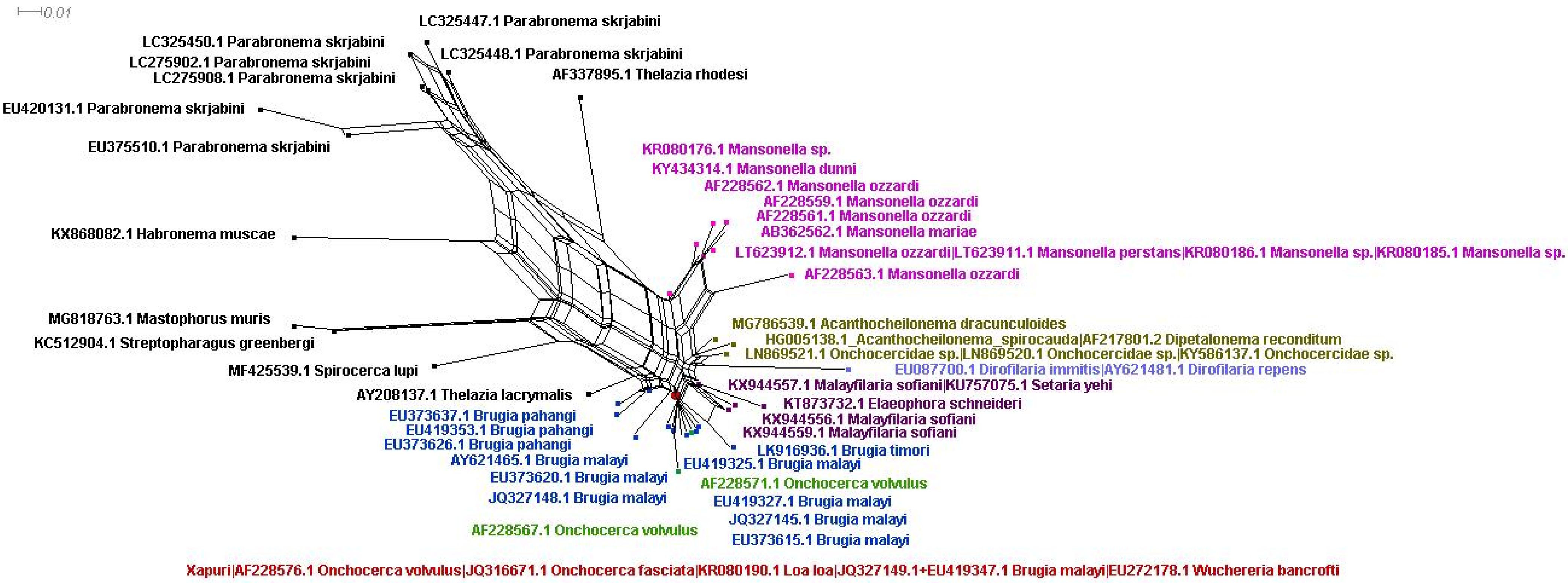
0.01
LC325447.1 Parabronema skrjabini
LC325450.1 Parabronema skrjabini
LC325448.1 Parabronema skrjabini
LC275902.1 Parabronema skrjabini
LC275908.1 Parabronema skrjabini
AF337895.1 Thelazia rhodesi
EU420131.1 Parabronema skrjabini
EU375510.1 Parabronema skrjabini
KR080176.1 Mansonella sp.
KY434314.1 Mansonella dunni
AF228562.1 Mansonella ozzardi
AF228559.1 Mansonella ozzardi
AF228561.1 Mansonella ozzardi
KX868082.1 Habronema muscae
AB362562.1 Mansonella mariae
LT623912.1 Mansonella ozzardi|LT623911.1 Mansonella perstans|KR080186.1 Mansonella sp.|KR080185.1 Mansonella sp.
AF228563.1 Mansonella ozzardi
MG818763.1 Mastophorus muris
MG786539.1 Acanthocheilonema dracunculoides
KC512904.1 Streptopharagus greenbergi
HG005138.1_Acanthocheilonema_spirocauda|AF217801.2 Dipetalonema reconditum
LN869521.1 Onchocercidae sp.|LN869520.1 Onchocercidae sp.|KY586137.1 Onchocercidae sp.
MF425539.1 Spirocerca lupi
EU087700.1 Dirofilaria immitis|AY621481.1 Dirofilaria repens
KX944557.1 Malayfilaria sofiani|KU757075.1 Setaria yehi
AY208137.1 Thelazia lacrymalis
KT873732.1 Elaeophora schneideri
EU373637.1 Brugia pahangi
KX944556.1 Malayfilaria sofiani
EU419353.1 Brugia pahangi
KX944559.1 Malayfilaria sofiani
EU373626.1 Brugia pahangi
LK916936.1 Brugia timori
AY621465.1 Brugia malayi
EU419325.1 Brugia malayi
EU373620.1 Brugia malayi
AF228571.1 Onchocerca volvulus
JQ327148.1 Brugia malayi
EU419327.1 Brugia malayi
JQ327145.1 Brugia malayi
AF228567.1 Onchocerca volvulus
EU373615.1 Brugia malayi
Xapuri|AF228576.1 Onchocerca volvulus|JQ316671.1 Onchocerca fasciata|KR080190.1 Loa loa|JQ327149.1+EU419347.1 Brugia malayi|EU272178.1 Wuchereria bancrofti

Figure S2

```
Position                                                10        20        30        40        50        60
                                              ....|....|....|....|....|....|....|....|....|....|....|....|....|..
Xapuri ITS[1]                                 AAACGGTGATTGGTGTCTATACTTTATCCAAGTTATCGCCCCGTCGATAACAATGAAGATAAAGCGA
AF228571.1 Onchocerca volvulus                ....................................................G..............
AF228567.1 Onchocerca volvulus                .............C..................C..................................
LK916936.1 Brugia timori                      ........................................G..............T...........
JQ327148.1 Brugia malayi                      .............C.....................................................
JQ327145.1 Brugia malayi                      ...................................C...............................
EU419327.1 Brugia malayi                      ............................................T......................
EU419325.1 Brugia malayi                      .....................................................C.............
EU373620.1 Brugia malayi                      ..................................................................G
EU373615.1 Brugia malayi                      ......................C............................................
EU419353.1 Brugia pahangi                     ........T..........................................................
EU373637.1 Brugia pahangi                     ........T................G.........................................
EU373626.1 Brugia pahangi                     .G......T..........................................................
MG786539.1 Acanthocheilonema dracunculoides   ...................................................G........G......
HG005138.1 Acanthocheilonema spirocauda[2]    ..............................................G....G...............
LN869521.1 Onchocercidae sp.[3]               ............................A......................G...............
LT623912.1 Mansonella ozzardi[4]              T..............................A..................TG...............
AF228563.1 Mansonella ozzardi                 T...A..........................A.....A.............G...............
AF228562.1 Mansonella ozzardi                 T........................G.....A..................TG...............
AF228561.1 Mansonella ozzardi                 T..................A...........A..................TG...............
AF228559.1 Mansonella ozzardi                 T..............C...............A..................TG...............
KR080176.1 Mansonella sp.                     ...............................A..................T................
KY434314.1 Mansonella dunni                   T...........................T..A..................TG...............
KX944559.1 Malayfilaria sofiani               ............................T..........................T...........
KX944557.1 Malayfilaria sofiani[5]            ............................T......................................
KX944556.1 Malayfilaria sofiani               .........C..................T......................................
KT873732.1 Elaeophora schneideri              ...........................GT.......T..............................
AY621465.1 Brugia malayi                      G.................................G................................
AB362562.1 Mansonella mariae                  T.......T......................A..................TG...............
EU087700.1 Dirofilaria immitis[6]             .....................T......T....................G.G...........AT..
AY208137.1 Thelazia lacrymalis                ........T...........T.............................TG...............
MF425539.1 Spirocerca lupi                    ........T...........T......T.G...........................A......A.G
AF337895.1 Thelazia rhodesi                   T.......T............T......T.T...................T.....T......AT..
KC512904.1 Streptopharagus greenbergi         C.......T....C.....AT...C.....................G.............G...A.G
KX868082.1 Habronema muscae                   C.......T..........A..G....TTG................G.T.......T.....C.T..
MG818763.1 Mastophorus muris                  C.......T....C.....AT...CG....................G.............G...A.G
LC275908.1 Parabronema skrjabini              C.......C...............C..TTG.A..................T.....T...G...T..
LC275902.1 Parabronema skrjabini              C.......C...............C..TTG.T..................T.....T...G...T..
LC325448.1 Parabronema skrjabini              C.......C...............C..TT..T.....C............T.....T...G...T..
LC325447.1 Parabronema skrjabini              C.....G.C...............C..TT..A.....C............T.....T...G...T..
LC325450.1 Parabronema skrjabini              C.......C...............C..TTG.A.....C............T.....T...G...T..
EU375510.1 Parabronema skrjabini              C.......T...............C..TGG.A.................GTG....T...G...T..
EU420131.1 Parabronema skrjabini              C.......T...............CG.TGG.A.................GTG....T..CG...T..
```

Supplementary information 1. Alignment 12S – Nucleotide alignment of the 12S gene used in the phylogenetic analysis represented in Fig. 2.

```
                                      10        20        30        40        50        60
                             ....|....|....|....|....|....|....|....|....|....|....|....|
GU138699.1 Setaria digitata mi  TTATTTC-ATTTTTT--GTAAAATATTTTGGTTTTTTT-----TTTTTGAAAAAG--TGC
AF538716.1 Brugia malayi mitoc  TTATGTT-ATTTTT---GTAAAATGTTTTAGTTTTTTTTA---TTATTTGAAGAGA-CAA
AP017686.1 Brugia timori mitoc  TTATGTT-ATTTTT---GTAAAATGTTTTAGTTTTTCTTA---TTATTTAAAAAGA-TAA
KP760318.1 Brugia pahangi vouc  TTATGTT-ATTTTT---GTAAAATGTTTTAGTTTTTAT-A---TTTTTTAAAAAGA-CAT
JN367461.1 Wuchereria bancroft  ATAT-TT-ATTTTT---GTAAAATGTTTTAGTTTTTTT-A---TTTTTTGAAAAGA-CAC
KP760339.1 Loa loa voucher 80Y  TTATATT-ATTTTT---GTAAAATGTTTTAATTTT-ATTA---TATTTT-TAAAGA-TAT
JF412320.1 Onchocercidae cf. M  NTATTTTAATTTTTT--GTAAAATGTTTTAGTTTA-TTTT---TGTTTTGAGGAAA-TAC
KP760340.1 Mansonella ozzardi   NTATTTTAAATTTTT--GTAAAATGTTTTAGTTTA-TTTT---TGTTTTGRGGAAA-TAC
LT623914.1 Mansonella ozzardi   NTATTTTAATTTTTT--GTAAAATGTTTTAGTTTA-TTTT---TGTTTTGAGGAAA-TAC
LT623913.1 Mansonella perstans  NTATTTTGATTTTTT--GTAAAATATTTCAATTT--TTTT---TTTTTTAAAAAAA-TTT
AM779823.1 Mansonella atelensi  NNNNTT-AATTTTTTTTGTAAAATATTTTAATTTTTATTT---TTCTTTGGAAAAA-TGT
AM779802.1 Mansonella perforat  NTATTTTAATTTTTTT-GTAAAATATTTTAATTTT-ATTT---TACTTTGAGAAGA-TAT
FR827906.1 Foleyella candezei   TTATTTT-ATTTTT---GTAAAATATTTTAATTTT--TTC---TTTTTTGTGAA-GATAT
HM773029.1 Chandlerella quisca  NTATTTT-TTGTTTTTTGTAAAATATTTCAATTTTTTTTG-A----TTTATAAAAGATGT
KX265050.1 Dirofilaria sp. 'ho  TTATGTT-TTTTTTT--GTAAAATGTTTTGATTTTTTT-GTATTTTTTTGTAA----TAT
KX265091.1 Dirofilaria repens   TTATGTT-TTTTTTTT-GTAAAATGTTTTGATTTTTTT-GTATTTTTTTGTAA----TAT
AJ537512.1 Dirofilaria immitis  TTATGTT-TTTTTTT--GTAAAATATTTAAATTTATTT-ATGTTTTTTTGTAA----TAT
MK391042.1 Onchocerca flexuosa  TTAAGTT-ATTTTTT--GTAAAATATTTTAATTTTTATTAT---TTTTTATTGTGG-TAT
MK391041.1 Onchocerca flexuosa  TTAAGTT-ATTTTTT--GTAAAATATTTTAATTTTTATTAT---TTTTTATTGTGG-TAT
MK391038.1 Onchocerca flexuosa  TTAAGTT-ATTTTTT--GTAAAATATTTTAATTTTTATTAT---TTTTTGTTGTGG-TAT
LT732683.1 Onchocerca flexuosa  TTAAGTT-ATTTTTT--GTAAAATATTTTAATTTTTATTAT---TTTTTGTTGTGG-TAT
LT732682.1 Onchocerca flexuosa  TTAAGTT-ATTTTTT--GTAAAATATTTTAATTTTTATTAT---TTTTTGTTGTGG-TAT
MK391037.1 Onchocerca flexuosa  TTAAGTT-ATTTTTT--GTAAAATATTTTAATTTTTATTAT---TTTTTGTTGTGG-TAT
MK391033.1 Onchocerca flexuosa  TTAAGTT-ATTTTTT--GTAAAATATTTTAATTTTTATTAT---TTTTTGTTGTGG-TAT
HQ214004.1 Onchocerca flexuosa  TTAAGTT-ATTTTTT--GTAAAATATTTTAATTTTTATTAT---TTTTTGTTGTGG-TAT
MK391034.1 Onchocerca flexuosa  TTAAGTT-ATTTTTT--GTAAAATATTTTAATTTTTATTAT---TTTTTGTTGTGG-TAT
MK391043.1 Onchocerca flexuosa  TTAAGTT-ATTTTTT--GTAAAATATTTTAATTTTTATTAT---TTTTTGTTGTGG-TAT
AP017692.1 Onchocerca flexuosa  TTAAGTT-ATTTTTT--GTAAAATATTTTAATTTTTATTAT---TTTTTGTTGTGG-TAT
AB972362.1 Onchocerca takaokai  TTATATT-ATTTTTTT-GTAAAATATTTTAATTTATTTTT----TTTTTGTTGAGA-TAT
AB972364.1 Onchocerca takaokai  TTATATT-ATTTTTTT-GTAAAATATTTTAATTTATTTTTT---TTTTTGTTGAGA-TAT
AB972363.1 Onchocerca takaokai  TTATATT-ATTTTTTT-GTAAAATATTTTAATTTATTTTT----TTTTTGTTGAGA-TAT
MH049488.1 Onchocerca sp. A AL  TTATGTTTATTTTTT--GTAAAATATTTTGATTTATTTTT---ATTTTT-TTAAGA-TGT
AM779811.1 Onchocerca suzukii   TTATATTTATTTTTT--GTAAAATGTTTTAATTTATTTTGT--TTTTTTTTTGAGA-TAT
AM779812.1 Onchocerca suzukii   TTATATTTATTTTTT--GTAAAATGTTTTAATTTATTTTGT--TTTTTTTTTGAGA-TAT
AB518879.1 Onchocerca sp. type  TTA-CTTTATTTTTT--GTAAAATGTTTTAATTTGATTTTG--TTTTT--TTAAGA-TAT
AB518877.1 Onchocerca sp. type  TTA-CTTTATTTTTT--GTAAAATGTTTTAATTTGATTTTG--TTTTT--TTAAGA-TAT
AB518878.1 Onchocerca sp. type  TTA-CTTTATTTTTT--GTAAAATGTTTTAATTTGATTTTG--TTTTT--TTAAGA-TAT
KC167341.1 Onchocerca ramachan  TTATGTT-ATTTTTT--GTAAAATATTTTAATTTATTATT---TTTTTTTTGGAGA-TGT
KC167340.1 Onchocerca ramachan  TTATGTATATTTTTT--GTAAAATATTTTAATTTATTATT---TTTTTTTTGGAGA-TGT
JX075208.1 Onchocerca cerviped  TTATGTT-ATTTTTT--GTAAAATATTTTAATTTGTTTT----ATTTTTTTAAAG--TGT
JX075207.1 Onchocerca cerviped  TTATGTT-ATTTTTT--GTAAAATATTTTAATTTGTTTT----ATTTTTTTAAAG--TGT
KP760345.2 Onchocerca armillat  TTATGTT-ATTTTTT--GTAAAATGTTTTAACTTTTTTTTT--ATTTTTT-GAAG--TAT
KX853314.1 Onchocerca armillat  TTATGTT-ATTTTTT--GTAAAATGTTTTAACTTTTTTTTT--ATTTTTT-GAAG--TAT
JN863696.1 Onchocerca lupi iso  TTATGTT-ATTTTTT--GTAAAATATTTTGATTTGTTTATT--TTTTTTTTTTTGG-TAT
GU365879.1 Onchocerca lupi 12S  TTATGTT-ATTTTTT--GTAAAATATTTTGATTTGTTTATT--TTTTTT-TT-TGG-TAT
AY462913.1 Onchocerca gibsoni   TTATGTT-ATTTTTT--GTAAAATATTTTAGTTTTTTTTTT---GTTTTCTTGTGG-TAT
AY462912.1 Onchocerca gibsoni   TTATGTT-ATTTTTT--GTAAAATATTTTAGTTTTTTTTTT---GTTTTCTTGTGG-TAT
MN985128.1 Onchocerca fasciata  TTATGTT-ATTTTTT--GTAAAATATTTTAGTTTTTTT-AT---GTTTTCTTATGG-TAT
MN985127.1 Onchocerca fasciata  TTATGTT-ATTTTTT--GTAAAATATTTTAGTTTTTTT-AT---GTTTTCTTATGG-TAT
JX075213.1 Onchocerca sp. QSM-  TTATGTT-ATTTTTT--GTAAAATATTTTAGTTTTTTTTTT---GTTTTCTTGTGA-TAT
JX075215.1 Onchocerca sp. QSM-  TTATGTT-ATTTTTT--GTAAAATATTTTAGTTTTTTTTTT---GTTTTCTTGTGA-TAT
JX075211.1 Onchocerca sp. QSM-  TTATGTT-ATTTTTT--GTAAAATATTTTAGTTTTTTTTT----GTTTTCTTGTGA-TAT
AY462926.1 Onchocerca linealis  TTACGTT-ATTTTTT--GTAAAATATTTTAGTTTTTTTT----AGTTT-CTTGTGG-TAT
AY462927.1 Onchocerca linealis  TTACGTT-ATTTTTT--GTAAAATATTTTAGTTTTTTTT----AGTTT-CTTGTGG-TAT
AY462924.1 Onchocerca linealis  TTACGTT-ATTTTTT--GTAAAATATTTTAGTTTTTTTT----AGTTT-CTTGTGG-TAT
AY462923.1 Onchocerca gutturos  TTATGTT-ATTTTTT--GTAAAATGTTTTAGGTTTTTTTTT---GTTTTGTTGTGA-TAT
KX181290.2 Onchocerca ochengi   TTATGTT-GTTTTTT--GTAAAATGTTTTGATTTTTTTT----AGTTT-CTTGTGG-TAT
KC167337.1 Onchocerca sp. 'Sii  TTATGTT-GTTTTTT--GTAAAATGTTTTGATTTTTTTT----AGTTT-CTTGTGG-TAT
KC167338.1 Onchocerca sp. 'Sii  TTATGTT-GTTTTTTT-GTAAAATGTTTTGATTTTTTTTT---AGTTT-CTTGTGG-TAT
KC167333.1 Onchocerca ochengi   TTATGTT-GTTTTTT--GTAAAATATTTTGATTTTTTTTTT--AGTTT-CTTGTGG-TAT
KC167334.1 Onchocerca ochengi   TTATGTT-GTTTTTT--GTAAAATATTTTGATTTTTTTTTT-TAGTTT-CTTGTGG-TAT
AY462914.1 Onchocerca ochengi   TTATGTT-GTTTTTT--GTAAAATATTTTGATTTTTTTTT---AGTTT-CTTGTGG-TAT
AY462915.1 Onchocerca ochengi   TTATGTT-GTTTTTT--GTAAAATATTTTGATTTTTTTTT---AGTTT-CTTGTGG-TAT
FM206484.1 Onchocerca ochengi   TTATGTT-GTTTTTT--GTAAAATATTTTGATTTTTTTTT---AGTTT-CTTGTGG-TAT
AY462916.1 Onchocerca ochengi   TTATGAT-GTTTTTT--GTAAAATATTTTGATTTTTTTTT---AGTTT-CTTGTGG-TAT
AY462917.1 Onchocerca ochengi   TTATGTT-GTTTTTT--GTAAAATATTTTGATTTTTTTTT---AGTTT-CTTGTGG-TAT
AP017694.1 Onchocerca ochengi   TTATGTT-GTTTTTT--GTAAAATATTTTGATTTTTTTT----AGTTT-CTTGTGG-TAT
NC_031891.2 Onchocerca ochengi  TTATGTT-GTTTTTT--GTAAAATATTTTGATTTTTTTT----AGTTT-CTTGTGG-TAT
KX181289.2 Onchocerca ochengi   TTATGTT-GTTTTTT--GTAAAATATTTTGATTTTTTTT----AGTTT-CTTGTGG-TAT
AP017693.1 Onchocerca ochengi   TTATGTT-GTTTTTT--GTAAAATATTTTGATTTTTTTT----AGTTT-CTTGTGG-TAT
AP017695.1 Onchocerca volvulus  TTATATT-GTTTTTT--GTAAAATATTTTAATTTTTTTTT---GGTTT-CTTATGG-TAT
AF015193.1 Onchocerca volvulus  TTATATT-GTTTTTT--GTAAAATATTTTAATTTTTTTTTT--AGTTT-CTTATGG-TAT
AY462920.1 Onchocerca volvulus  TTATATT-GTTTTTT--GTAAAATATTTTAATTTTTTTTT---AGTTT-CTTATGG-TAT
KT599912.1 Onchocerca volvulus  TTATATT-GTTTTTT--GTAAAATATTTTAATTTTTTTTT---AGTTT-CTTATGG-TAT
KC167339.1 Onchocerca volvulus  TTATATT-GTTTTTT--GTAAAATATTTTAATTTTTTTTT---AGTTT-CTTATGG-TAT
```

```
                                       70        80        90       100       110       120
                               ....|....|....|....|....|....|....|....|....|....|....|....|
GU138699.1 Setaria digitata mi TTA-AAATTTGGTTT--TTGAACTGGATTAGTACCCAGGTAAACAAAATTTATTAATTCG
AF538716.1 Brugia malayi mitoc TTA-AAATTTGGTTT--TTGAACTGGATTAGTACCCAGGTAATCAAAATTAATTAATTCG
AP017686.1 Brugia timori mitoc TTA-AAATTTGGTTT--TTGAACTGGATTAGTACCCAGGTAATCAAAATTTATTAATTCG
KP760318.1 Brugia pahangi vouc TTA-AAATTTGTTTT--TTGAACTGGATTAGTACCCAGGTAATCAAAATTAATTAATTCG
JN367461.1 Wuchereria bancroft TCA-AAATTTGGTTT--TTGAACTGGATTAGTACCCAGGTAATCAAAGTTTATTAATTCG
KP760339.1 Loa loa voucher 80Y TGA-AAATTTGTTTG--TTGAACTGGATTAGTACCCAGGTAATCAAAATTTATTAATTCG
JF412320.1 Onchocercidae cf. M TTA-AAATTTGTTTTA-TTGAACTGGATTAGTACCCAGGTAATCAAAATTAATTAATTCG
KP760340.1 Mansonella ozzardi  TTA-AAATTTGTTTTA-TTGAACTGGATTAGTACCCCGGTAATCAAAATTAATTAATTCG
LT623914.1 Mansonella ozzardi  TTA-AAATTTGTTTTA-TTGAACTGGATTAGTACCCAGGTAATCAAAATTAATTAATTCG
LT623913.1 Mansonella perstans TTA-AAATTTGGTTTAGTTGAACTGGATTAGTACCCAGGTAATCAAAATTAATTAATTCG
AM779823.1 Mansonella atelensi TTA-AAATTTGTTTTA-TTGAACTGGATTAGTACCCAGGTAATCAAAGTTTATTAATTCG
AM779802.1 Mansonella perforat CTA-AAATTTGTTTT--TTGAACTGGATTAGTACCCAGGTAATCAAAATTTATTAATTCG
FR827906.1 Foleyella candezei  TGA-AAATTTGATTT--TTGAACTGGATTAGTACCCAGGTAATCAAAATTTATTAATTCG
HM773029.1 Chandlerella quisca TAA-AAATTTGGTTT--TTGAACTGGATTAGTACCCAGGTAATCAAAATTTATTAATTCG
KX265050.1 Dirofilaria sp. 'ho TTA-AAATTTGTTTT--TTGAACTGGATTAGTACCCAGGTAATCAAAGTTTATTAATTCG
KX265091.1 Dirofilaria repens  TTA-AAATTTGTTTT--TTGAACTGGATTAGTACCCAGGTAATCAAAGTTTATTAATTCG
AJ537512.1 Dirofilaria immitis TGA-AAATTTGGTGT--TTGAACTGGATTAGTACCCAGGTAATCAAAATTTATTAATTCG
MK391042.1 Onchocerca flexuosa -CAAAAATTTGTTTT--TTGAACTGGATTAGTACCCAGGTAATCAAAATTTATTAATTCG
MK391041.1 Onchocerca flexuosa -CAAAAATTTGTTTT--TTGAACTGGATTAGTACCCAGGTAATCAAAATTTATTAATTCG
MK391038.1 Onchocerca flexuosa -CAAAAATTTGTTTT--TTGAACTGGATTAGTACCCAGGTAATCAAAATTTATTAATTCG
LT732683.1 Onchocerca flexuosa -CAAAAATTTGTTTT--TTGAACTGGATTAGTACCCAGGTAATCAAAATTTATTAATTCG
LT732682.1 Onchocerca flexuosa -CAAAAATTTGTTTT--TTGAACTGGATTAGTACCCAGGTAATCAAAATTTATTAATTCG
MK391037.1 Onchocerca flexuosa -CAAAAATTTGTTTT--TTGAACTGGATTAGTACCCAGGTAATCAAAATTTATTAATTCG
MK391033.1 Onchocerca flexuosa -CAAAAATTTGTTTT--TTGAACTGGATTAGTACCCAGGTAATCAAAATTTATTAATTCG
HQ214004.1 Onchocerca flexuosa -CAAAAATTTGTTTT--TTGAACTGGATTAGTACCCAGGTAATCAAAATTTATTAATTCG
MK391034.1 Onchocerca flexuosa -CAAAAATTTGTTTT--TTGAACTGGATTAGTACCCAGGTAATCAAAATTTATTAATTCG
MK391043.1 Onchocerca flexuosa -CAAAAATTTGTTTT--TTGAACTGGATTAGTACCCAGGTAATCAAAATTTATTAATTCG
AP017692.1 Onchocerca flexuosa -CAAAAATTTGTTTT--TTGAACTGGATTAGTACCCAGGTAATCAAAATTTATTAATTCG
AB972362.1 Onchocerca takaokai -CAAAAATTTGTTTT--TTGAACTGGATTAGTACCCAGGTAATCAAAATTAATTAATTCG
AB972364.1 Onchocerca takaokai -CAAAAATTTGTTTT--TTGAACTGGATTAGTACCCAGGTAATCAAAATTAATTAATTCG
AB972363.1 Onchocerca takaokai -CAAAAATTTGTTTT--TTGAACTGGATTAGTACCCAGGTAATCAAAATTAATTAATTCG
MH049488.1 Onchocerca sp. A AL -CAAAAATTTGTTTT--TTGAACTGGATTAGTACCCAGGTAATCAAAATTTATTAATTCG
AM779811.1 Onchocerca suzukii  -CAAAAATTTGGTTT--TTGAACTGGATTAGTACCCAGGTAATCAAAGTTTATTAATTCG
AM779812.1 Onchocerca suzukii  -CAAAAATTTGGTTT--TTGAACTGGATTAGTACCCAGGTAATCAAAGTTTATTAATTCG
AB518879.1 Onchocerca sp. type T-AAAAATTTGGTTT--TTGAACTGGATTAGTACCCAGGTAATCAAAGTTTATTAATTCG
AB518877.1 Onchocerca sp. type T-AAAAATTTGGTTT--TTGAACTGGATTAGTACCCAGGTAATCAAAGTTTATTAATTCG
AB518878.1 Onchocerca sp. type T-AAAAATTTGGTTT--TTGAACTGGATTAGTACCCAGGTAATCAAAGTTTATTAATTCG
KC167341.1 Onchocerca ramachan -CAAAAATTTGTTTT--TTGAACTGGATTAGTACCCAGGTAATCAAAATTTAATAATTCG
KC167340.1 Onchocerca ramachan -CAAAAATTTGTTTT--TTGAACTGGATTAGTACCCAGGTAATCAAAATTTAATAATTCG
JX075208.1 Onchocerca cerviped -CAAAAATTTGTTTT--TTGAACTGGATTAGTACCCAGGTAATCAAAATTTATTAATTCG
JX075207.1 Onchocerca cerviped -CAAAAATTTGTTTT--TTGAACTGGATTAGTACCCAGGTAATCAAAATTTATTAATTCG
KP760345.2 Onchocerca armillat -CAAAAATTTGTTTT--TTGAACTGGATTAGTACCCAGGTAATCAAAATTTATTAATTCG
KX853314.1 Onchocerca armillat -CAAAAATTTGTTTT--TTGAACTGGATTAGTACCCAGGTAATCAAAATTTATTAATTCG
JN863696.1 Onchocerca lupi iso -CAAAAATTTGTTTT--TTGAACTGGATTAGTACCCAGGTAATCAAAATTTATTAATTCG
GU365879.1 Onchocerca lupi 12S -CAAAAATTTGTTTT--TTGAACTGGATTAGTACCCAGGTAATCAAAATTTATTAATTCG
AY462913.1 Onchocerca gibsoni  -CAAAAATTTGTTTT--TTGAACTGGATTAGTACCCAGGTAATCAAAATTTAATAATTCG
AY462912.1 Onchocerca gibsoni  -CAAAAATTTGTTTT--TTGAACTGGACTAGTACCCAGGTAATCAAAATTTAATAATTCG
MN985128.1 Onchocerca fasciata -CAAAAATTTGTTTT--TTGAACTGGATTAGTACCCAGGTAATCAAAATTTAATAATTCG
MN985127.1 Onchocerca fasciata -CAAAAATTTGTTTT--TTGAACTGGATTAGTACCCAGGTAATCAAAATTTAATAATTCG
JX075213.1 Onchocerca sp. QSM- -CAAAAATTTGTTTT--TTGAACTGGATTAGTACCCAGGTAATCAAAATTTAGTAATTCG
JX075215.1 Onchocerca sp. QSM- -CAAAAATTTGTTTT--TTGAACTGGATTAGTACCCAGGTAATCAAAATTTAGTAATTCG
JX075211.1 Onchocerca sp. QSM- -CAAAAATTTGTTTT--TTGAACTGGATTAGTACCCAGGTAATCAAAATTTAGTAATTCG
AY462926.1 Onchocerca linealis -CAAAAATTTGTTTT--TTGAACTGGATTAGTACCCAGGTAATCAAAATTTAATAATTCG
AY462927.1 Onchocerca linealis -CAAAAATTTGTTTT--TTGAACTGGATTAGTACCCAGGTAATCAAAATTTAATAATTCG
AY462924.1 Onchocerca linealis -CAAAAATTTGTTTT--TTGAACTGGATTAGTACCCAGGTAATCAAAATTTAATAATTCG
AY462923.1 Onchocerca gutturos -CAAAAATTTGTTTT--TTGAACTGGATTAGTACCCAGGTAATCAAAGTTTAATAATTCG
KX181290.2 Onchocerca ochengi  -CAAAAATTTGTTTT--TTGAACTGGATTAGTACCCAGGTAATCAAAATTTAATAATTCG
KC167337.1 Onchocerca sp. 'Sii -CAAAAATTTGTTTT--TTGAACTGGATTAGTACCCAGGTAATCAAAATTTAATAATTCG
KC167338.1 Onchocerca sp. 'Sii -CAAAAATTTGTTTT--TTGAACTGGATTAGTACCCAGGTAATCAAAATTTAATAATTCG
KC167333.1 Onchocerca ochengi  -CAAAAATTTGTTTT--TTGAACTGGATTAGTACCCAGGTAATCAAAATTTAATAATTCG
KC167334.1 Onchocerca ochengi  -CAAAAATTTGTTTT--TTGAACTGGATTAGTACCCAGGTAATCAAAATTTAATAATTCG
AY462914.1 Onchocerca ochengi  -CAAAAATTTGTTTT--TTGAACTGGATTAGTACCCAGGTAATCAAAATTTAATAATTCG
AY462915.1 Onchocerca ochengi  -CAAAAATTTGTTTT--TTGAACTGGATTAGTACCCAGGTAATCAAAATTTAATAATTCG
FM206484.1 Onchocerca ochengi  -CAAAAATTTGTTTT--TTGAACTGGATTAGTACCCAGGTAATCAAAATTTAATAATTCG
AY462916.1 Onchocerca ochengi  -CAAAAATTTGTTTT--TTGAACTGGATTAGTACCCAGGTAATCAAAATTTAATAATTCG
AY462917.1 Onchocerca ochengi  -CAAAAATTTGTTTT--TTGAACTGGATTAGTACCCAGGTAATCAAAATTTAATAATTCG
AP017694.1 Onchocerca ochengi  -CAAAAATTTGTTTT--TTGAACTGGATTAGTACCCAGGTAATCAAAATTTAATAATTCG
NC_031891.2 Onchocerca ochengi -CAAAAATTTGTTTT--TTGAACTGGATTAGTACCCAGGTAATCAAAATTTAATAATTCG
KX181289.2 Onchocerca ochengi  -CAAAAATTTGTTTT--TTGAACTGGATTAGTACCCAGGTAATCAAAATTTAATAATTCG
AP017693.1 Onchocerca ochengi  -CAAAAATTTGTTTT--TTGAACTGGATTAGTACCCAGGTAATCAAAATTTAATAATTCG
AP017695.1 Onchocerca volvulus -CAAAAATTTGTTTT--TTGAACTGGATTAGTACCCAGGTAATCAAAATTTAATAATTCG
AF015193.1 Onchocerca volvulus -CAAAAATTTGTTTT--TTGAACTGGATTAGTACCCAGGTAATCAAAATTTAATAATTCG
AY462920.1 Onchocerca volvulus -CAAAAATTTGTTTT--TTGAACTGGATTAGTACCCAGGTAATCAAAATTTAATAATTCG
KT599912.1 Onchocerca volvulus -CAAAAATTTGTTTT--TTGAACTGGATTAGTACCCAGGTAATCAAAATTTAATAATTCG
KC167339.1 Onchocerca volvulus -CAAAAATTTGTTTT--TTGAACTGGATTAGTACCCAGGTAATCAAAATTTAATAATTCG
```

```
                                             130       140       150       160       170       180
                                    ....|....|....|....|....|....|....|....|....|....|....|....|
GU138699.1 Setaria digitata mi    GGAGTAAAGTTTTTTTTAAACCGAAAAAATATTGACTGACTTCGGATTTTTCTTTGGAAC
AF538716.1 Brugia malayi mitoc    GGAGTAAAGTTTTATTTAAACCGAAAAAATATTGACTGACTTTGGATTTTTCTTTGGAAT
AP017686.1 Brugia timori mitoc    GGAGTAAAGTTTTATTTAAACCGAAAAAATATTGACTGACTTTGGATTTTTCTTTGGAAT
KP760318.1 Brugia pahangi vouc    GGAGTAAAGTTTTGTTTAAACCGAAAAAATATTGACTGACCTTGGATTTTTCTTTGGAAT
JN367461.1 Wuchereria bancroft    GGAGTAAAGTTTTGTTTAAACCGAAAAAATATTGACTGACTTTAGATTTCTCTTTGGAAC
KP760339.1 Loa loa voucher 80Y    GGAGTAAAGTTTTGTTTAAACCGAAAAAATATTGACTGACTTTAGATTTTTCTTTGGAAC
JF412320.1 Onchocercidae cf. M    GGAGTAAAGTTTTGTTTAAACCGAAAAAATATTGACTGACTTTAGATTTCTTTTTGGAAC
KP760340.1 Mansonella ozzardi     GGAGTAAAGTTTTGTTTAAACCGAAAAAATATTGACTGACTTTAGATTTCTTTTTGGAAC
LT623914.1 Mansonella ozzardi     GGAGTAAAGTTTTGTTTAAACCGAAAAAATATTGACTGACTTTAGATTTCTTTTTGGAAC
LT623913.1 Mansonella perstans    GGAGTAAAGTTTTGTTTAAACCGAAAAAATATTGACTGACTCTGGATTTCTTTTTGGAAT
AM779823.1 Mansonella atelensi    GGAGTAAAGTTTTATTTAAACCGAAAAAATATTGACTGACTTTGGATTTTTTTTTGGAAC
AM779802.1 Mansonella perforat    GGAGTAAAGTTTTATTTAAACCGAAAAAATATTGACTGACTCTAGATTTTTTTTTGGACA
FR827906.1 Foleyella candezei     GGAGTAAAGTTTTGTTTAAACCGAAAAAATATTGACTGACCTTAGATTTCTCTTTGGAAT
HM773029.1 Chandlerella quisca    GGAGTAAAGTTGTATTTAAACCGTAAAAATATTGGCTGACTTTAAATTTTTCTTTGGAAT
KX265050.1 Dirofilaria sp. 'ho    GGAGTAAAGTTTTGTTTAAACCGAAAAAATATTGACTGACTTTAGATTTTTCTTTGGAAT
KX265091.1 Dirofilaria repens     GGAGTAAAGTTTTGTTTAAACCGAAAAAATATTGACTGACTTTAGATTTTTCTTTGGAAT
AJ537512.1 Dirofilaria immitis    GGAGTAAAGTTTTGTTTAAACCGAAAAAATATTGACTGACTTTAGATTTTTCTTTGGAAT
MK391042.1 Onchocerca flexuosa    GGAGTAAAGTTTTGTTTAAACCGAAAAAATATTGACTGACCTTAGATTTTTCTTTGGAAT
MK391041.1 Onchocerca flexuosa    GGAGTAAAGTTTTGTTTAAACCGAAAAAATATTGACTGACCTTAGATTTTTCTTTGGAAT
MK391038.1 Onchocerca flexuosa    GGAGTAAAGTTTTGTTTAAACCGAAAAAATATTGACTGACCTTAGATTTTTCTTTGGAAT
LT732683.1 Onchocerca flexuosa    GGAGTAAAGTTTTGTTTAAACCGAAAAAATATTGACTGACCTTAGATTTTTCTTTGGAAT
LT732682.1 Onchocerca flexuosa    GGAGTAAAGTTTTGTTTAAACCGAAAAAATATTGACTGACCTTAGATTTTTCTTTGGAAT
MK391037.1 Onchocerca flexuosa    GGAGTAAAGTTTTGTTTAAACCGAAAAAATATTGACTGACCTTAGATTTTTCTTTGGAAT
MK391033.1 Onchocerca flexuosa    GGAGTAAAGTTTTGTTTAAACCGAAAAAATATTGACTGACCTTAGATTTTTCTTTGGAAT
HQ214004.1 Onchocerca flexuosa    GGAGTAAAGTTTTGTTTAAACCGAAAAAATATTGACTGACCTTAGATTTTTCTTTGGAAT
MK391034.1 Onchocerca flexuosa    GGAGTAAAGTTTTGTTTAAACCGAAAAAATATTGACTGACCTTAGATTTTTCTTTGGAAT
MK391043.1 Onchocerca flexuosa    GGAGTAAAGTTTTGTTTAAACCGAAAAAATATTGACTGACCTTAGATTTTTCTTTGGAAT
AP017692.1 Onchocerca flexuosa    GGAGTAAAGTTTTGTTTAAACCGAAAAAATATTGACTGACCTTAGATTTTTCTTTGGAAT
AB972362.1 Onchocerca takaokai    GGAGTAAAGTTTTATTTAAACCGAAAAAATATTGACTGACTTTAGATTTTTCTTTGGAAT
AB972364.1 Onchocerca takaokai    GGAGTAAAGTTTTATTTAAACCGAAAAAATATTGACTGACTTTAGATTTTTCTTTGGAAT
AB972363.1 Onchocerca takaokai    GGAGTAAAGTTTTATTTAAACCGAAAAAATATTGACTGACTTTAGATTTTTCTTTGGAAT
MH049488.1 Onchocerca sp. A AL    GGAGTAAAGTTTTGTTTAAACCGAAAAAATATTGACTGACTTTAGATTTTTCTTTGGAAT
AM779811.1 Onchocerca suzukii     GGAGTAAAGTTTTGTTTAAACCGAAAAAATATTGACTGACCTTGGATTTTTCTTTGGAAC
AM779812.1 Onchocerca suzukii     GGAGTAAAGTTTTGTTTAAACCGAAAAAATATTGACTGACCTTGGATTTTTCTTTGGAAC
AB518879.1 Onchocerca sp. type    GGAGTAAAGTTTTGTTTAAACCGAAAAAATATTGACTGACCTTAGATTTTTCTTTGGAAT
AB518877.1 Onchocerca sp. type    GGAGTAAAGTTTTGTTTAAACCGAAAAAATATTGACTGACCTTAGATTTTTCTTTGGAAT
AB518878.1 Onchocerca sp. type    GGAGTAAAGTTTTGTTTAAACCGAAAAAATATTGACTGACCTTAGATTTTTCTTTGGAAT
KC167341.1 Onchocerca ramachan    GGAGTAAAGTTTTGTTTAAACCGAAAAAATATTGACTGACTTTAGATTTTTCTTTGGAAC
KC167340.1 Onchocerca ramachan    GGAGTAAAGTTTTGTTTAAACCGAAAAAATATTGACTGACTTTAGATTTTTCTTTGGAAC
JX075208.1 Onchocerca cerviped    GGAGTAAAGTTTTGTTTAAACCGAAAAAATATTGACTGACCTTAGATTTTTCTTTGGAAC
JX075207.1 Onchocerca cerviped    GGAGTAAAGTTTTGTTTAAACCGAAAAAATATTGACTGACCTTAGATTTTTCTTTGGAAC
KP760345.2 Onchocerca armillat    GGAGTAAAGTTGTGTTTAAACCGAAAAAATATTGACTGACCTTAGATTTTTCTTTGGAAC
KX853314.1 Onchocerca armillat    GGAGTAAAGTTGTGTTTAAACCGAAAAAATATTGACTGACCTTAGATTTTTCTTTGGAAC
JN863696.1 Onchocerca lupi iso    GGAGTAAAGTTTTGTTTAAACCGAAAAAATATTGACTGACTTTAGATTTTTCTTTGGAAT
GU365879.1 Onchocerca lupi 12S    GGAGTAAAGTTTTGTTTAAACCGAAAAAATATTGACTGACTTTAGATTTTTCTTTGGAAT
AY462913.1 Onchocerca gibsoni     GGAGTAAAGTTTTGTTTAAACCGAAAAAATATTGACTGACTTTAGATTTTTCTTTGGAAC
AY462912.1 Onchocerca gibsoni     GGAGTAAAGTTTTGTTTAAACCGAAAAAATATTGACTGACTTTAGATTTTTCTTTGGAAC
MN985128.1 Onchocerca fasciata    GGAGTAAAGTTTTGTTTAAACCGAAAAAATATTGACTGACTTTAGATTTTTCTTTGGAAC
MN985127.1 Onchocerca fasciata    GGAGTAAAGTTTTGTTTAAACCGAAAAAATATTGACTGACTTTAGATTTTTCTTTGGAAC
JX075213.1 Onchocerca sp. QSM-    GGAGTAAAGTTTTGTTTAAACCGAAAAAATATTGACTGACTTTAGATTTTTCTTTGGAAT
JX075215.1 Onchocerca sp. QSM-    GGAGTAAAGTTTTGTTTAAACCGAAAAAATATTGACTGACTTTAGATTTTTCTTTGGAAT
JX075211.1 Onchocerca sp. QSM-    GGAGTAAAGTTTTGTTTAAACCGAAAAAATATTGACTGACTTTAGATTTTTCTTTGGAAT
AY462926.1 Onchocerca linealis    GGAGTAAAGTTTTATTTAAACCGAAAAAATATTGACTGACTTTAGATTTTTCTTTGGAAT
AY462927.1 Onchocerca linealis    GGAGTAAAGTTTTATTTAAACCGAAAAAATATTGACTGACTTTAGATTTTTCTTTGGAAT
AY462924.1 Onchocerca linealis    GGAGTAAAGTTTTATTTAAACCGAAAAAATATTGACTGACTTTAGATTTTTCTTTGGAAT
AY462923.1 Onchocerca gutturos    GGAGTAAAGTTTTGTTTAAACCGAAAAAATATTGACTGACTTTAGATTTTTCTTTGGAAT
KX181290.2 Onchocerca ochengi     GGAGTAAAGTTTTGTTTAAACCGAAAAAATATTGACTGACTTTAGATTTTTCTTTGGAAT
KC167337.1 Onchocerca sp. 'Sii    GGAGTAAAGTTTTGTTTAAACCGAAAAAATATTGACTGACTTTAGATTTTTCTTTGGAAT
KC167338.1 Onchocerca sp. 'Sii    GGAGTAAAGTTTTGTTTAAACCGAAAAAATATTGACTGACTTTAGATTTTTCTTTGGAAT
KC167333.1 Onchocerca ochengi     GGAGTAAAGTTTTGTTTAAACCGAAAAAATATTGACTGACTTTAGATTTTTCTTTGGAAC
KC167334.1 Onchocerca ochengi     GGAGTAAAGTTTTGTTTAAACCGAAAAAATATTGACTGACTTTAGATTTTTCTTTGGAAC
AY462914.1 Onchocerca ochengi     GGAGTAAAGTTTTGTTTAAACCGAAAAAATATTGACTGACTTTAGATTTTTCTTTGGAAC
AY462915.1 Onchocerca ochengi     GGAGTAAAGTTTTGTTTAAACCGAAAAAATATTGACTGACTTTAGATTTTTCTTTGGAAC
FM206484.1 Onchocerca ochengi     GGAGTAAAGTTTTGTTTAAACCGAAAAAATATTGACTGACTTTAGATTTTTCTTTGGAAC
AY462916.1 Onchocerca ochengi     GGAGTAAAGTTTTGTTTAAACCGAAAAAATATTGACTGACTTTAGATTTTTCTTTGGAAC
AY462917.1 Onchocerca ochengi     GGAGTAAAGTTTTGTTTAAACCGAAAAAATATTGACTGACTTTAGATTTTTCTTTGGAAC
AP017694.1 Onchocerca ochengi     GGAGTAAAGTTTTGTTTAAACCGAAAAAATATTGACTGACTTTAGATTTTTCTTTGGAAC
NC_031891.2 Onchocerca ochengi    GGAGTAAAGTTTTGTTTAAACCGAAAAAATATTGACTGACTTTAGATTTTTCTTTGGAAC
KX181289.2 Onchocerca ochengi     GGAGTAAAGTTTTGTTTAAACCGAAAAAATATTGACTGACTTTAGATTTTTCTTTGGAAC
AP017693.1 Onchocerca ochengi     GGAGTAAAGTTTTGTTTAAACCGAAAAAATATTGACTGACTTTAGATTTTTCTTTGGAAC
AP017695.1 Onchocerca volvulus    GGAGTAAAGTTTTATTTAAACCGAAAAAATATTGACTGACTTTAGATTTTTCTTTGGAAT
AF015193.1 Onchocerca volvulus    GGAGTAAAGTTTTATTTAAACCGAAAAAATATTGACTGACTTTAGATTTTTCTTTGGAAT
AY462920.1 Onchocerca volvulus    GGAGTAAAGTTTTATTTAAACCGAAAAAATATTGACTGACTTTAGATTTTTCTTTGGAAT
KT599912.1 Onchocerca volvulus    GGAGTAAAGTTTTATTTAAACCGAAAAAATATTGACTGACTTTAGATTTTTCTTTGGAAT
KC167339.1 Onchocerca volvulus    GGAGTAAAGTTTTATTTAAACCGAAAAAATATTGACTGACTTTAGATTTTTCTTTGGAAT
```

```
                                                   190       200       210       220       230       240
                                          ....|....|....|....|....|....|....|....|....|....|....|....|
GU138699.1 Setaria digitata mi            ATGTGTTTTTT----GGAGAGTCCTCCCTTGTTGCTGGTTTTGTTG-GCTCATGTATGAT
AF538716.1 Brugia malayi mitoc            ATGCGTAT-------GGAGAGCCCTCCTTTTTAGTTAACTCTATTG-GCACATGTATGAT
AP017686.1 Brugia timori mitoc            ATGAGTAT-------GGAGAGCCCTCCTTTTTAGTTAATCCTATTG-GCACATGTATGAT
KP760318.1 Brugia pahangi vouc            ATGCGTAT-------GGAGAGCCCTCTTTTTTGGTGAGTCTTATCG-GCACATGTATGAT
JN367461.1 Wuchereria bancroft            ATGCGTAT-------GGAGAGCCCTCCTTTTTGGTTAATCTTGTTG-GCACATGTATGAT
KP760339.1 Loa loa voucher 80Y            ATGTGTAT-------GGAGAGTCCTCTTTTTTAGTGAGTTTTATTG-GCACATGTATGAT
JF412320.1 Onchocercidae cf. M            ATGTGTAT-------GGAGAGCCCTCCTTTTAAGTAAATTTTTTTTTGCTCATGTATGAT
KP760340.1 Mansonella ozzardi             CTGTGTAT-------GGAGAGCCCCCCCTTTAAGTTAAATTTTTTTTGCTCATGTATGAT
LT623914.1 Mansonella ozzardi             ATGTGTAT-------GGAGAGCCCTCCTTTTAAGTAAATTTTTTTTTGCTCATGTATGAT
LT623913.1 Mansonella perstans            ATGTGTAT-------GGAGAGCCCTCTTTTATAGTAAATCTTTTTTTGCACATGTATGAT
AM779823.1 Mansonella atelensi            ATGTGTAT-------GGAGAGCCCTCTTTAGTAGTAAATTTTTTTTTGCACATGTATGAT
AM779802.1 Mansonella perforat            ATGTGTAT-------GGAGAGCCCTCCTTTATAGTG-AATCTTATTGGCACATGTATGAT
FR827906.1 Foleyella candezei             ATGTG-TTT------GGAGAGCCCTCTTTTATAGTAATTTCTTATTTGCACATGTATGAT
HM773029.1 Chandlerella quisca            ATGTG-ATT------GGAGAGCCCTCCTTTTTGGTAGATTTTTTTG-GTACATGTATGAT
KX265050.1 Dirofilaria sp. 'ho            ATGTG-TTTTT----GGAGAGCCCTCTTTTTTGGTGAATTTTATTG-GCACATGTATGAT
KX265091.1 Dirofilaria repens             ATGTG-TTTTT----GGAGAGCCCTCTTTTTTGGTGAATTTTATTG-GCACATGTATGAT
AJ537512.1 Dirofilaria immitis            ATGTG-TTTTTTT--GGAGAGCCCTCTTTTATAGTGAATTTTGTTG-GCGCATGTATGAT
MK391042.1 Onchocerca flexuosa            ATGTG-TTTTGTT--GGAGAGCCCTCCTTATTAGTGAATTTTGTTG-GCACATGTATGAT
MK391041.1 Onchocerca flexuosa            ATGTG-TTTTGTT--GGAGAGCCCTCCTTATTAGTGAATTTTGTTG-GCACATGTATGAT
MK391038.1 Onchocerca flexuosa            ATGTG-TTTTGTT--GGAGAGCCCTCCTTATTAGTGAATTTTGTTG-GCACATGTATGAT
LT732683.1 Onchocerca flexuosa            ATGTG-TTTTGTT--GGAGAGCCCTCCTTATTAGTGAATTTTGTTG-GCACATGTATGAT
LT732682.1 Onchocerca flexuosa            ATGTG-TTTTGTT--GGAGAGCCCTCCTTATTAGTGAATTTTGTTG-GCACATGTATGAT
MK391037.1 Onchocerca flexuosa            ATGTG-TTTTGTT--GGAGAGCCCTCCTTATTAGTGAATTTTGTTG-GCACATGTATGAT
MK391033.1 Onchocerca flexuosa            ATGTG-TTTTGTT--GGAGAGCCCTCCTTATTAGTGAATTTTGTTG-GCACATGTATGAT
HQ214004.1 Onchocerca flexuosa            ATGTG-TTTTGTT--GGAGAGCCCTCCTTATTAGTGAATTTTGTTG-GCACATGTATGAT
MK391034.1 Onchocerca flexuosa            ATGTG-TTTTGTT--GGAGAGCCCTCCTTATTAGTGAATTTTGTTG-GCACATGTATGAT
MK391043.1 Onchocerca flexuosa            ATGTG-TTTTGTT--GGAGAGCCCTCCTTATTAGTGAATTTTGTTG-GCACATGTATGAT
AP017692.1 Onchocerca flexuosa            ATGTG-TTTTGTT--GGAGAGCCCTCCTTATTAGTGAATTTTGTTG-GCACATGTATGAT
AB972362.1 Onchocerca takaokai            ATGTG-TTTT-----GGAGAGCCCTCCTTTTTAGTGATTTTTGTTG-GCACATGTATGAT
AB972364.1 Onchocerca takaokai            ATGTG-TTTT-----GGAGAGCCCTCCTTTTTAGTGATTTTTGTTG-GCACATGTATGAT
AB972363.1 Onchocerca takaokai            ATGTG-TTTT-----GGAGAGCCCTCCTTTTTAGTGATTTTTGTTG-GCACATGTATGAT
MH049488.1 Onchocerca sp. A AL            ATGTG-TTTT-----GGAGAGCCCTCCTTTTTGGTAAGTTTTGTTG-GCACATGTATGAT
AM779811.1 Onchocerca suzukii             ATGTG-TTTTTATT-GGAGAGCCCTCTTTTTTGGTAAATTTTGTTG-GCACATGTATGAT
AM779812.1 Onchocerca suzukii             ATGTG-TTTTTATT-GGAGAGCCCTCTTTTTTGGTAAATTTTGTTG-GCACATGTATGAT
AB518879.1 Onchocerca sp. type            ATGTG-TTTTGCT--GGAGAGCCCTCTTTTTTGGTGAATTTTGTTG-GCACATGTATGAT
AB518877.1 Onchocerca sp. type            ATGTG-TTTTGCT--GGAGAGCCCTCTTTTTTGGTGAATTTTGTTG-GCACATGTATGAT
AB518878.1 Onchocerca sp. type            ATGTG-TTTTGCT--GGAGAGCCCTCTTTTTTGGTGAATTTTGTTG-GCACATGTATGAT
KC167341.1 Onchocerca ramachan            ATGTG-TTTTACT--GGAGAGCCCTCTTTTTTAGTGAATTTTGTTG-GCACATGTATGAT
KC167340.1 Onchocerca ramachan            ATGTG-TTTTACT--GGAGAGCCCTCTTTTTTAGTGAATTTTGTTG-GCACATGTATGAT
JX075208.1 Onchocerca cerviped            ATGTG-TTTTTAATTGGAGAGCCCTCTTTTTTGGTGAATTCTATTG-GCACATGTATGAT
JX075207.1 Onchocerca cerviped            ATGTG-TTTTTAATTGGAGAGCCCTCTTTTTTGGTGAATTCTATTG-GCACATGTATGAT
KP760345.2 Onchocerca armillat            ATGTG-TTTTGCT--GGAGAGCCCTCCTTTTTGGTAAATTTTGTTG-GCACATGTATGAT
KX853314.1 Onchocerca armillat            ATGTG-TTTTGCT--GGAGAGCCCTCCTTTTTGGTAAATTTTGTTG-GCACATGTATGAT
JN863696.1 Onchocerca lupi iso            ATGTGATTTTGTT--GGAGAGCCCTCTTTTTTGGTAAATTTTGTTG-GCACATGTATGAT
GU365879.1 Onchocerca lupi 12S            ATGTGATTTTGTT--GGAGAGCCCTCTTTTTTGGTAAATTTTGTTG-GCACATGTATGAT
AY462913.1 Onchocerca gibsoni             ATGTGATTTT-CT--GGAGAGCCCTCTTTTTTAGTGATTTTTATTG-GCACATGTATGAT
AY462912.1 Onchocerca gibsoni             ATGTGATTTT-CT--GGAGAGCCCTCTTTTTTAGTGATTTTTATTG-GCACATGTATGAT
MN985128.1 Onchocerca fasciata            ATGTGATTT-GCT--GGAGAGCCCTCTTTTTTGGTGAGTTTTATTG-GCACATGTATGAT
MN985127.1 Onchocerca fasciata            ATGTGATTT-GCT--GGAGAGCCCTCTTTTTTGGTGAGTTTTATTG-GCACATGTATGAT
JX075213.1 Onchocerca sp. QSM-            ATGTGATTT-GCT--GGAGAGCCCTCTTTTTTGGTGAATTTTATTG-GCACATGTATGAT
JX075215.1 Onchocerca sp. QSM-            ATGTGATTT-GCT--GGAGAGCCCTCTTTTTTGGTGAATTTTATTG-GCACATGTATGAT
JX075211.1 Onchocerca sp. QSM-            ATGTGATTT-GCT--GGAGAGCCCTCTTTTTTGGTGAATTTTATTG-GCACATGTATGAT
AY462926.1 Onchocerca linealis            ATGTGATTT-GCT--GGAGAGCCCTCTTCTTTGGTGAGTTTTATTG-GCACATGTATGAT
AY462927.1 Onchocerca linealis            ATGTGATTT-GCT--GGAGAGCCCTCTTTTTTGGTGAGTTTTATTG-GCATATGTATGAT
AY462924.1 Onchocerca linealis            ATGTGATTT-GCT--GGAGAGCCCTCTTTTTTGGTGAGTTTTATTG-GCACATGTATGAT
AY462923.1 Onchocerca gutturos            ATGTGATTT-GCT--GGAGAGCCCTCTTTCTTGGTGAGTTTTATTG-GCACATGTATGAT
KX181290.2 Onchocerca ochengi             ATGTGATTT-GCT--GGAGAGCCCTCTTTTTTGGTGAATTTTGTTG-GCACATGTATGAT
KC167337.1 Onchocerca sp. 'Sii            ATGTGATTT-GCT--GGAGAGCCCTCTTTTTTGGTGAATTTTGTTG-GCACATGTATGAT
KC167338.1 Onchocerca sp. 'Sii            ATGTGATTT-GCT--GGAGAGCCCTCTTTTTTGGTGAATTTTGTTG-GCACATGTATGAT
KC167333.1 Onchocerca ochengi             ATGTGATTT-GCT--GGAGAGCCCTCTTTTTTGGTGAATTTTGTTG-GCACATGTATGAT
KC167334.1 Onchocerca ochengi             ATGTGATTT-GCT--GGAGAGCCCTCTTTTTTGGTGAATTTTGTTG-GCACATGTATGAT
AY462914.1 Onchocerca ochengi             ATGTGATTT-GCT--GGAGAGCCCTCTTTTTTGGTGAATTTTGTTG-GCACATGTATGAT
AY462915.1 Onchocerca ochengi             ATGTGATTT-GCT--GGAGAGCCCTCTTTTTTGGTGAATTTTGTTG-GCACATGTATGAT
FM206484.1 Onchocerca ochengi             ATGTGATTT-GCT--GGAGAGCCCTCTTTTTTGGTGAATTTTGTTG-GCACATGTATGAT
AY462916.1 Onchocerca ochengi             ATGTGATTT-GCT--GGAGAGCCCTCTTTTTTGGTGAATTTTGTTG-GCACATGTATGAT
AY462917.1 Onchocerca ochengi             ATGTGATTT-GCT--GGAGAGCCCTCTTTTTTGGTGAATTTTGTTG-GCACATGTATGAT
AP017694.1 Onchocerca ochengi             ATGTGATTT-GCT--GGAGAGCCCTCTTTTTTGGTGAATTTTGTTG-GCACATGTATGAT
NC_031891.2 Onchocerca ochengi            ATGTGATTT-GCT--GGAGAGCCCTCTTTTTTGGTGAATTTTGTTG-GCACATGTATGAT
KX181289.2 Onchocerca ochengi             ATGTGATTT-GCT--GGAGAGCCCTCTTTTTTGGTGAATTTTGTTG-GCACATGTATGAT
AP017693.1 Onchocerca ochengi             ATGTGATTT-GCT--GGAGAGCCCTCTTTTTTGGTGAATTTTGTTG-GCACATGTATGAT
AP017695.1 Onchocerca volvulus            ATGTGATTT-GCT--GGAGAGCCCTCTTTTTTGGTGAATTTTGTTG-GCACATGTATGAT
AF015193.1 Onchocerca volvulus            ATGTGATTT-GCT--GGAGAGCCCTCTTTTTTGGTGAATTTTGTTG-GCACATGTATGAT
AY462920.1 Onchocerca volvulus            ATGTGATTT-GCT--GGAGAGCCCTCTTTTTTGGTGAATTTTGTTG-GCACATGTATGAT
KT599912.1 Onchocerca volvulus            ATGTGATTT-GCT--GGAGAGCCCTCTTTTTTGGTGAATTTTGTTG-GCACATGTATGAT
KC167339.1 Onchocerca volvulus            ATGTGATTT-GCT--GGAGAGCCCTCTTTTTTGGTGAATTTTGTTG-GCACATGTATGAT
```

```
                                         250       260       270       280       290       300
                                ....|....|....|....|....|....|....|....|....|....|....|....|
GU138699.1 Setaria digitata mi  TGTTTAGCTTTTATTTT-TTTTGTAATGCTATTTTTTGTTTTT----ATTTAAAACAGAT
AF538716.1 Brugia malayi mitoc  TGTTTAGTTTTTATTTTATTT-GTAATGCTTTT-AT-GTTTTGT--CATTTAAAACAGAT
AP017686.1 Brugia timori mitoc  TGTTTAGTTTTTATTTTATTT-GTAATGCTTTT-AT-GTTATGT--CATTTAAAACATAT
KP760318.1 Brugia pahangi vouc  TGTTTAGTTTTTATTTTATTT-GTAATGCTTTTTAT-GTTTTGT--CATTTAAAACAGAT
JN367461.1 Wuchereria bancroft  TGTTTAGTTTTTATTTT-TTTTGTAATGCTTTT-TT-GTTTTGT--CATTAAAAACAGAT
KP760339.1 Loa loa voucher 80Y  TGTTTAGTTTTTATGTTATTTTGTGATGCTTTTTTTTG-TTT--AA-TTGAAAAACAGAT
JF412320.1 Onchocercidae cf. M  TGTTTAGTTTTTATGTTATTTTGTAATGCTTTTTT---TTTTT-AA--TCTAAAACAGAT
KP760340.1 Mansonella ozzardi   TGTTTAGTTTTTATGTTATTTTGTAATGCTTTTTT---TTTT--AA--TTTAAAACAGAT
LT623914.1 Mansonella ozzardi   TGTTTAGTTTTTATGTTATTTTGTAATGCTTTTTT---TTTT--AA--TTTAAAACAGAT
LT623913.1 Mansonella perstans  TGTTTAGTTTTTATTTTATTTTGTAATGCTTTATT---TTTA-GA---TTAAAAACAGAT
AM779823.1 Mansonella atelensi  TGTTTAGTTTTTATGTTTTTTTGTAATGCTTT------TTTGGGA---TTTAGAACAGAT
AM779802.1 Mansonella perforat  TGTTTAGTTTTTATATTTTTTTGTAATGCTTTC-----TTTGAGA---TTTAAAACAGAT
FR827906.1 Foleyella candezei   TGTTTAGTTTTTATTTTATTTTGTAATGCTTTTTTGT-ATTTT-A-CATTAAAAACAGAT
HM773029.1 Chandlerella quisca  TGTTTAGTTTTTATTTTATTTTGTAATGCTTTT-----TTTTT-AG--TAAAAAACAGAT
KX265050.1 Dirofilaria sp. 'ho  TGTTTAGTTTTTATGTTATTT-GTGTTGCTTTTTT--GTTTTTTTGCATTTAAAACAGAT
KX265091.1 Dirofilaria repens   TGTTTAGTTTTTATGTTATTT-GTGTTGCTTTTTT--GTTTTTTTGCATTTAAAACAGAT
AJ537512.1 Dirofilaria immitis  TGTTTAGTTTTTACTTT-TTTGGTAATGCTTTGT---GTTTTATA-CATTTAAAACAGAT
MK391042.1 Onchocerca flexuosa  TGTTTAGTTTTTATTTTATTTTGTAATGCTTTTT---GTTTT--GGCATTTAAAACAGAT
MK391041.1 Onchocerca flexuosa  TGTTTAGTTTTTATTTTATTTTGTAATGCTTTTT---GTTTT--GGCATTTAAAACAGAT
MK391038.1 Onchocerca flexuosa  TGTTTAGTTTTTATTTTATTTTGTAATGCTTTTT---GTTTT--GGCATTTAAAACAGAT
LT732683.1 Onchocerca flexuosa  TGTTTAGTTTTTATTTTATTTTGTAATGCTTTTT---GTTTT--GGCATTTAAAACAGAT
LT732682.1 Onchocerca flexuosa  TGTTTAGTTTTTATTTTATTTTGTAATGCTTTTT---GTTTT--GGCATTTAAAACAGAT
MK391037.1 Onchocerca flexuosa  TGTTTAGTTTTTATTTTATTTTGTAATGCTTTTT---GTTTT--GGCATTTAAAACAGAT
MK391033.1 Onchocerca flexuosa  TGTTTAGTTTTTATTTTATTTTGTAATGCTTTTT---GTTTT--GGCATTTAAAACAGAT
HQ214004.1 Onchocerca flexuosa  TGTTTAGTTTTTATTTTATTTTGTAATGCTTTTT---GTTTT--GGCATTTAAAACAGAT
MK391034.1 Onchocerca flexuosa  TGTTTAGTTTTTATTTTATTTTGTAATGCTTTTT---GTTTT--GGCATTTAAAACAGAT
MK391043.1 Onchocerca flexuosa  TGTTTAGTTTTTATTTTATTTTGTAATGCTTTTT---GTTTT--GGCATTTAAAACAGAT
AP017692.1 Onchocerca flexuosa  TGTTTAGTTTTTATTTTATTTTGTAATGCTTTTT---GTTTT--GGCATTTAAAACAGAT
AB972362.1 Onchocerca takaokai  TGTTTAGTTTTTATTTTGTTTTGTAATGCTTTTT---GTTTTTTA-CATTTAAAACAGAT
AB972364.1 Onchocerca takaokai  TGTTTAGTTTTTATTTTGTTTTGTAATGCTTTTT---GTTTTTTA-CATTTAAAACAGAT
AB972363.1 Onchocerca takaokai  TGTTTAGTTTTTATTTTGTTTTGTAATGCTTTTT---GTTTTTTA-CATTTAAAACAGAT
MH049488.1 Onchocerca sp. A AL  TGTTTAGTTTTTATTTT-TTTTGTAATGCTTTTT---GTTTT--GGCATTTAAAATAGAT
AM779811.1 Onchocerca suzukii   TGTTTAGTTTTTATTTTATATTGGAATGCTTTT----GTTTT--GGCTTTTAAAACAGAT
AM779812.1 Onchocerca suzukii   TGTTTAGTTTTTATTTTATATTGTAATGCTTTT----GTTTT--GGCTTTTAAAACAGAT
AB518879.1 Onchocerca sp. type  TGTTTAGTTTTTATTTTATTTTGTAATGCTTTTT---GTGTT--GGCATTTAAAACAGAT
AB518877.1 Onchocerca sp. type  TGTTTAGTTTTTATTTTATTTTGTAATGCTTTTT---GTGTT--GGCATTTAAAACAGAT
AB518878.1 Onchocerca sp. type  TGTTTAGTTTTTATTTTATTTTGTAATGCTTTTT---GTGTT--GGCATTTAAAACAGAT
KC167341.1 Onchocerca ramachan  TGTTTAGTTTTTATTTTGTTTTGTAATGCTTTTT---GTTTT--GGCTTTTAAAACAGAT
KC167340.1 Onchocerca ramachan  TGTTTAGTTTTTATTTTGTTTTGTAATGCTTTTT---GTTTT--GGCTTTTAAAACAGAT
JX075208.1 Onchocerca cerviped  TGTTTAGTTTTTATTTTATCTTGTAATGCTTGTTT--GTTTT--GGCATTTAAAACAGAT
JX075207.1 Onchocerca cerviped  TGTTTAGTTTTTATTTTATCTTGTAATGCTTGTTT--GTTTT--GGCATTTAAAACAGAT
KP760345.2 Onchocerca armillat  TGTTTAGTTTTTATTTTATTTTGTAATGCTTATATT-GTTTT--GGCATTTAAAACAGAT
KX853314.1 Onchocerca armillat  TGTTTAGTTTTTATTTTATTTTGTAATGCTTATATT-GTTTT--GGCATTTAAAACAGAT
JN863696.1 Onchocerca lupi iso  TGTTTAGTTTTTATTTTATTTTGTAATGCTTTTTTT-GTTTT--GGCATTTAAAACAGAT
GU365879.1 Onchocerca lupi 12S  TGTTTAGTTTTTATTTTATTTTGTAATGCTTTTTTT-GTTTT--GGCATTTAAAACAGAT
AY462913.1 Onchocerca gibsoni   TGTTTAGTTTTTATTTTATTTTGTAATGCTTTTAT--GTTTT--GGCATTTAAAACAGAT
AY462912.1 Onchocerca gibsoni   TGTTTAGTTTTTATTTTATTTTGTAATGCTTTTAT--GTTTT--GGCATTTAAAACAGAT
MN985128.1 Onchocerca fasciata  TGTTTAGTTTTTATTTTATTTTGTAATGCTTTTTT--GTTTT--GGCATTTAAAACAGAT
MN985127.1 Onchocerca fasciata  TGTTTAGTTTTTATTTTATTTTGTAATGCTTTTTT--GTTTT--GGCATTTAAAACAGAT
JX075213.1 Onchocerca sp. QSM-  TGTTTAGTTTTTATTTTATTTTGTAATGCTTTTTTT-GTTTT--GGCATTAAGAACAGAT
JX075215.1 Onchocerca sp. QSM-  TGTTTAGTTTTTATTTTATTTTGTAATGCTTTTTTT-GTTTT--GGCATTAAGAACAGAT
JX075211.1 Onchocerca sp. QSM-  TGTTTAGTTTTTATTTTATTTTGTAATGCTTTTTTT-GTTTT--GGCATTAAGAACAGAT
AY462926.1 Onchocerca linealis  TGTTTAGTTTTTATTTTATTTTGTAGTGCTTTTT---GTTTT--GGCATTTAAAACAGAT
AY462927.1 Onchocerca linealis  TGTTTAGCTTTTATTTTATTTTGTAGTGCTTTTT---GTTTT--GGCATTTAAAACAGAT
AY462924.1 Onchocerca linealis  TGTTTAGTTTTTATTTTATTTTGCAATGCTTTTTTC-GTCTT--GGCATTTAAAACAGAT
AY462923.1 Onchocerca gutturos  TGTTTAGTTTTTATTTTATTTTGTAATGCTTTTTT--GTTTT--GGCATTTAAAACAGAT
KX181290.2 Onchocerca ochengi   TGTTTAGTTTTTATTTTATTTTGTAATGCTTTGTT--GTTTT--GGCATTAAAAACAGAT
KC167337.1 Onchocerca sp. 'Sii  TGTTTAGTTTTTATTTTATTTTGTAATGCTTTGTT--GTTTT--GGCATTAAAAACAGAT
KC167338.1 Onchocerca sp. 'Sii  TGTTTAGTTTTTATTTTATTTTGTAATGCTTTGTT--GTTTT--GGCATTAAAAACAGAT
KC167333.1 Onchocerca ochengi   TGTTTAGTTTTTATTTTATTTTGTAATGCTTTGTT--GTTTT--GGCATTAAAAACAGAT
KC167334.1 Onchocerca ochengi   TGTTTAGTTTTTATTTTATTTTGTAATGCTTTGTT--GTTTT--GGCATTAAAAACAGAT
AY462914.1 Onchocerca ochengi   TGTTTAGTTTTTATTTTATTTTGTAATGCTTTGTT--GTTTT--GGCATTAAAAACAGAT
AY462915.1 Onchocerca ochengi   TGTTTAGTTTTTATTTTATTTTGTAATGCTTTGTT--GTTTT--GGCATTAAAAACAGAT
FM206484.1 Onchocerca ochengi   TGTTTAGTTTTTATTTTATTTTGTAATGCTTTGTT--GTTTT--GGCATTAAAAACAGAT
AY462916.1 Onchocerca ochengi   TGTTTAGTTTTTATTTTATTTTGTAATGCTTTGTT--GTTTT--GGCATTAAAAACAGAT
AY462917.1 Onchocerca ochengi   TGTTTAGTTTTTATTTTATTTTGTAATGCTTTGTT--GTTTT--GGCATTAAAAACAGAT
AP017694.1 Onchocerca ochengi   TGTTTAGTTTTTATTTTATTTTGTAATGCTTTGTT--GTTTT--GGCATTAAAAACAGAT
NC_031891.2 Onchocerca ochengi  TGTTTAGTTTTTATTTTATTTTGTAATGCTTTGTT--GTTTT--GGCATTAAAAACAGAT
KX181289.2 Onchocerca ochengi   TGTTTAGTTTTTATTTTATTTTGTAATGCTTTGTT--GTTTT--GGCATTAAAAACAGAT
AP017693.1 Onchocerca ochengi   TGTTTAGTTTTTATTTTATTTTGTAATGCTTTGTT--GTTTT--GGCATTAAAAACAGAT
AP017695.1 Onchocerca volvulus  TGTTTAGTTTTTATTTTATTTTGTAATGCTTTATT--GTTTT--GGCATTAAAAACAGAT
AF015193.1 Onchocerca volvulus  TGTTTAGTTTTTATTTTATTTTGTAATGCTTTATT--GTTTT--GGCATTAAAAACAGAT
AY462920.1 Onchocerca volvulus  TGTTTAGTTTTTATTTTATTTTGTAATGCTTTATT--GTTTT--GGCATCAAAAACAGAT
KT599912.1 Onchocerca volvulus  TGTTTAGTTTTTATTTTATTTTGTAATGCTTTATT--GTTTT--GGCATTAAAAACAGAT
KC167339.1 Onchocerca volvulus  TGTTTAGTTTTTATTTTATTTTGTAATGCTTTATT--GTTTT--GGCATTAAAAACAGAT
```

```
                                                    310       320       330       340       350       360
                                           ....|....|....|....|....|....|....|....|....|....|....|....|
GU138699.1 Setaria digitata mi             ATATATTTGGTTTATGAATCAGTTA-TCATGTGTTACTATTATTAATTTTTTTC-GGATT
AF538716.1 Brugia malayi mitoc             ATATATTTGGCTTATGGGTTTGTGA-TCGTGTATTACTATTATTAATTTTTTTT-GGATT
AP017686.1 Brugia timori mitoc             ATATATTTGGCTTATGGATTTATGA-TCGTGTGTTACTATTATTAATTTTTTTTTGGATT
KP760318.1 Brugia pahangi vouc             ATATATTTGGCTTATGGACTTATAA-TCGTGTGTTACTATTATTAATTTTTTTT-GGATT
JN367461.1 Wuchereria bancroft             ATATATTTGGCTTATGGATTTGTTA-TCGTGTGTTACTATTATTAATTTTTTTT-GGATT
KP760339.1 Loa loa voucher 80Y             ATATATTTGGTTTATAAGCTTGTTAATCATGTGTTACTATTATTAATTTTTTTT-GGATC
JF412320.1 Onchocercidae cf. M             AAATACTTGGCTAATGGGTTTATGA-TCATGTGTTACTATTAATAATTTTTTTT-GGATA
KP760340.1 Mansonella ozzardi              AAATACTTGGCTAATGGGTTTATGA-TCATGTGTTACTATTAATAATTTTTTTT-GGATA
LT623914.1 Mansonella ozzardi              AAATACTTGGCTAATGGGTTTATGA-TCATGTGTTACTATTAATAATTTTTTTT-GGATA
LT623913.1 Mansonella perstans             AAATACTTGGCTTATGGGTTTATGAATCATGTATTACTATTAAAAATTTTTTTT-GGATG
AM779823.1 Mansonella atelensi             ATATATTTGGCTTATGGGTTTATTAATCATGTGTTACTATTAGAAATATTTTTT-GGATG
AM779802.1 Mansonella perforat             ATGTACTTGGTTTATGGGTTTGTTGGACATGTGTTACTATTAATAATTTTTTTT-GGAT-
FR827906.1 Foleyella candezei              ATATATTTGGTTTATGATTTTATTGTTCATGTATTACTATTATTAATTTTTTTT-GGATT
HM773029.1 Chandlerella quisca             ATATACTTGGCTTATGAATCTATTTTTCATGTATTACTATTATTAATTTTTTTT-GGAT-
KX265050.1 Dirofilaria sp. 'ho             ATATATTTGGCTTATAAATTTATTTTTCATGTATTACTATTATTAATTTTTTTT-GGATT
KX265091.1 Dirofilaria repens              ATATATTTGGCTTATAAATTTATTTTTCATGTATTACTATTATTAATTTTTTTT-GGATT
AJ537512.1 Dirofilaria immitis             ATATATTTGGCTTATGGATTTATTTTTCATGTGTTACTATTGTTAATTTTCTTT-GGATT
MK391042.1 Onchocerca flexuosa             ATATACTTGGCTTATAAATTTATTTTTCATGTATTACTATTATAAATTTTTTTT-GGATT
MK391041.1 Onchocerca flexuosa             ATATACTTGGCTTATAAATTTATTTTTCATGTATTACTATTATAAATTTTTTTT-GGATT
MK391038.1 Onchocerca flexuosa             ATATACTTGGCTTATAAATTTATTTTTCATGTATTACTATTATAAATTTTTTTT-GGATT
LT732683.1 Onchocerca flexuosa             ATATACTTGGCTTATAAATTTATTTTTCATGTATTACTATTATAAATTTTTTTT-GGATT
LT732682.1 Onchocerca flexuosa             ATATACTTGGCTTATAAATTTATTTTTCATGTATTACTATTATAAATTTTTTTT-GGATT
MK391037.1 Onchocerca flexuosa             ATATACTTGGCTTATAAATTTATTTTTCATGTATTACTATTATAAATTTTTTTT-GGATT
MK391033.1 Onchocerca flexuosa             ATATACTTGGCTTATAAATTTATTTTTCATGTATTACTATTATAAATTTTTTTT-GGATT
HQ214004.1 Onchocerca flexuosa             ATATACTTGGCTTATAAATTTATTTTTCATGTATTACTATTATAAATTTTTTTT-GGATT
MK391034.1 Onchocerca flexuosa             ATATACTTGGCTTATAAATTTATTTTTCATGTATTACTATTATAAATTTTTTTT-GGATT
MK391043.1 Onchocerca flexuosa             ATATACTTGGCTTATAAATTTATTTTTCATGTATTACTATTATAAATTTTTTTT-GGATT
AP017692.1 Onchocerca flexuosa             ATATACTTGGCTTATAAATTTATTTTTCATGTATTACTATTATAAATTTTTTTT-GGATT
AB972362.1 Onchocerca takaokai             ATATATTTGGCTTATAAATTTATTTTTCATGTATTACTATTATAAATTTTTTTT-GGATT
AB972364.1 Onchocerca takaokai             ATATATTTGGCTTATAAATTTATTTTTCATGTATTACTATTATAAATTTTTTTT-GGATT
AB972363.1 Onchocerca takaokai             ATATATTTGGCTTATAAATTTATTTTTCATGTATTACTATTATAAATTTTTTTT-GGATT
MH049488.1 Onchocerca sp. A AL             ATATACTTGGCTTATGAATTTATTTTTCATGTATTACTATTTTAAATTTTATTT-GGATT
AM779811.1 Onchocerca suzukii              ATATATTTGGCTTATAAATTTATTTTTCATGTGTTACTATTATAAATTTTTTTT-GGATT
AM779812.1 Onchocerca suzukii              ATATATTTGGCTTATAAATTTATTTTTCATGTGTTACTATTATAAATTTTTTTT-GGATT
AB518879.1 Onchocerca sp. type             ATATACTTGGCTTATAAATTTATTTTTCATGTGTTACTATTATAAATTTTTTTT-GGATT
AB518877.1 Onchocerca sp. type             ATATACTTGGCTTATAAATTTATTTTTCATGTGTTACTATTATAAATTTTTTTT-GGATT
AB518878.1 Onchocerca sp. type             ATATACTTGGCTTATAAATTTATTTTTCATGTGTTACTATTATAAATTTTTTTT-GGATT
KC167341.1 Onchocerca ramachan             ATATACTTGGCTTATGAATTTATTTTTCATGTGTTACTATTATAAATTTTTTTT-GGATT
KC167340.1 Onchocerca ramachan             ATATACTTGGCTTATGAATTTATTTTTCATGTGTTACTATTATAAATTTTTTTT-GGATT
JX075208.1 Onchocerca cerviped             ATATATTTGGCTTATGGATTTATTTTTCATGTGTTACTATTTTAAATTTTTTTT-GGATG
JX075207.1 Onchocerca cerviped             ATATATTTGGCTTATGGATTTATTTTTCATGTGTTACTATTTTAAATTTTTTTT-GGATG
KP760345.2 Onchocerca armillat             ATATATTTGGCTTATGGATTTATTTTTCATGTGTTACTATTATAAATTTTTTTT-GGATT
KX853314.1 Onchocerca armillat             ATATATTTGGCTTATGGATTTATTTTTCATGTGTTACTATTATAAATTTTTTTT-GGATT
JN863696.1 Onchocerca lupi iso             ATATATTTGGTTTATAAATTTATTTTTCATGTGTTACTATTATAAATTTTTTTT-GGATT
GU365879.1 Onchocerca lupi 12S             ATATATTTGGTTTATAAATTTATTTTTCATGTGTTACTATTATAAATTTTTTTT-GGATT
AY462913.1 Onchocerca gibsoni              ATATATTTGGCTTATAAATTTATGTTTCATGTGTTACTATTATGAATTTTTTTT-GGATT
AY462912.1 Onchocerca gibsoni              ATATATTTGGCTTATAAATTTATGTTTCATGTGTTACTATTATGAATTTTTTTT-GGATT
MN985128.1 Onchocerca fasciata             ATATACTTGGCTTATGAACTCATGTTTCATGTGTTACTATTATAAATTTTTTTT-GGATT
MN985127.1 Onchocerca fasciata             ATATACTTGGCTTATGAACTCATGTTTCATGTGTTACTATTATAAATTTTTTTT-GGATT
JX075213.1 Onchocerca sp. QSM-             ATATATTTGGCTTATGGATTCATGTTTCATGTGTTACTATTATAAATTTTTTTT-GGATT
JX075215.1 Onchocerca sp. QSM-             ATATATTTGGCTTATGGATTCATGTTTCATGTGTTACTATTATAAATTTTTTTT-GGATT
JX075211.1 Onchocerca sp. QSM-             ATATATTTGGCTTATGGATTCATGTTTCATGTGTTACTATTATAAATTTTTTTT-GGATT
AY462926.1 Onchocerca linealis             ATATATTTGGCTTATGAATTCATGTTTCATGTGTTACTATTATAAATTTTTTTT-GGATT
AY462927.1 Onchocerca linealis             ATATATTTGGCTTATGAATTCATGTTTCATGTGTTACTATTATAAATTTTTTTT-GGATT
AY462924.1 Onchocerca linealis             ATATATTTGGCTTATAAATTTATGTTTCATGTGTTACTATTATGAATTTTTTTT-GGATT
AY462923.1 Onchocerca gutturos             ATATATTTGGCTTATGAGTTCATGTTTCATGTGTTACTATTATGAATTTTTTTT-GGA-T
KX181290.2 Onchocerca ochengi              ATATATTTGGCTTATGAATTTATGTTTCATGTGTTACTATTATGAATTTTTTTT-GGATT
KC167337.1 Onchocerca sp. 'Sii             ATATATTTGGCTTATGAATTTATGTTTCATGTGTTACTATTATGAATTTTTTTT-GGATT
KC167338.1 Onchocerca sp. 'Sii             ATATATTTGGCTTATGAATTTATGTTTCATGTGTTACTATTATGAATTTTTTTT-GGATT
KC167333.1 Onchocerca ochengi              ATATATTTGGCTTATGAATTTATGTTTCATGTGTTACTATTATGAATTTTTTTT-GGATT
KC167334.1 Onchocerca ochengi              ATATATTTGGCTTATGAATTTATGTTTCATGTGTTACTATTATGAATTTTTTTT-GGATT
AY462914.1 Onchocerca ochengi              ATATATTTGGCTTATGAATTTATGTTTCATGTGTTACTATTATGAATTTTTTTT-GGATT
AY462915.1 Onchocerca ochengi              ATATATTTGGCTTATGAATTTATGTTTCATGTGTTACTATTATGAATTTTTTTT-GGGTT
FM206484.1 Onchocerca ochengi              ATATATTTGGCTTATGAATTTATGTTTCATGTGTTACTATTATGAATTTTTTTT-GGATT
AY462916.1 Onchocerca ochengi              ATATATTTGGCTTATGAATTTATGTTTCATGTGTTACTATTATGAATTTTTTTT-GGATT
AY462917.1 Onchocerca ochengi              ATATATTTGGCTTATGAATTTATGTTTCATGTGTTACTATTATGAATTTTTTTT-GGATT
AP017694.1 Onchocerca ochengi              ATATATTTGGCTTATGAATTTATGTTTCATGTGTTACTATTATGAATTTTTTTT-GGATT
NC_031891.2 Onchocerca ochengi             ATATATTTGGCTTATGAATTTATGTTTCATGTGTTACTATTATGAATTTTTTTT-GGATT
KX181289.2 Onchocerca ochengi              ATATATTTGGCTTATGAATTTATGTTTCATGTGTTACTATTATGAATTTTTTTT-GGATT
AP017693.1 Onchocerca ochengi              ATATATTTGGCTTATGAATTTATGTTTCATGTGTTACTATTATGAATTTTTTTT-GGATT
AP017695.1 Onchocerca volvulus             ATATATTTGGCTTATGAATTTATGTTTCATGTGTTACTATTATGAATTTTTTTT-GGATT
AF015193.1 Onchocerca volvulus             ATATATTTGGCTTATGAATTTATGTTTCATGTGTTACTATTATGAATTTTTTTT-GGATT
AY462920.1 Onchocerca volvulus             ATATATTTGGCTTATGAATTTATGTTTCATGTGTTACTATTATGAATTTTTTTT-GGATT
KT599912.1 Onchocerca volvulus             ATATATTTGGCTTATGAATTTATGTTTCATGTGTTACTATTATGAATTTTTTTT-GGATT
KC167339.1 Onchocerca volvulus             ATATATTTGGCTTATGAATTTATGTTTCATGTGTTACTATTATGAATTTTTTTT-GGATT
```

```
                                              370       380       390       400
                                          ....|....|....|....|....|....|....|...
GU138699.1 Setaria digitata mi           TAATTTTTA-TTTTTTTTT--GAAGTTGGAAAAGAAAGTAATT
AF538716.1 Brugia malayi mitoc           ATTTTTTTA-TTTTTTTTT--GAAATTGGAAAAGAAAGTAATT
AP017686.1 Brugia timori mitoc           ATTTTTTTA-TTTTTTTTT--GAAATTGGAAAAGAAAGTAATT
KP760318.1 Brugia pahangi vouc           ATTTTTTTA-TTTTTTTTT--GAAATTGGAAAAGAAAGTAATT
JN367461.1 Wuchereria bancroft           ATTTTTT-A-TTTTTTTTT--GAAATTGGAAAAGAAAGTAATT
KP760339.1 Loa loa voucher 80Y           TTTTTTTTA-ATATTTTGT--GAAATTGGAAAAGAAAGTAATT
JF412320.1 Onchocercidae cf. M           GTTTTTT-A-TTTTTTATTT-GAAATTGGAAAAAAAAGTAATT
KP760340.1 Mansonella ozzardi            GTTTTTT-A-TTTTTTATTT-GAAATTGGAAAAAAAAGTAATT
LT623914.1 Mansonella ozzardi            GTTTTTT-A-TTTTTTATTT-GAAATTGGAAAAAAAAGTAATT
LT623913.1 Mansonella perstans           GTTTTTT-A-TTTTTTATTT-GAAATTGGAAAAAAAAGTAATT
AM779823.1 Mansonella atelensi           ATTTTTTTA-GTTTGTATCT-GAAATTGGACAAAAAAGTAATT
AM779802.1 Mansonella perforat           AATTTTTTTTATTATTTTTT-GAAATTGGAAAAAAAAGTAATT
FR827906.1 Foleyella candezei            AATTTTTT--ATTTTTTTTT-GAAATTGGAAAAGAAAGTAATT
HM773029.1 Chandlerella quisca           AATATTTTA-ATTTTTATTT-GAAATTGGAAAAGAAAGTAATT
KX265050.1 Dirofilaria sp. 'ho           TATTTTTT--ATTTTATTTTTGAAATTGGAAAAGAAAGTAATT
KX265091.1 Dirofilaria repens            TATTTTTT--ATTTTGTTTTTGAAATTGGAAAAGAAAGTAATT
AJ537512.1 Dirofilaria immitis           AATTTTT-A-ATTTTTTTGTTGAAATTGGAAAAGAAAGTAATT
MK391042.1 Onchocerca flexuosa           AATTTTTT--ATTTTTTTTT-GAAATTGGAAAAGAAAGTAATT
MK391041.1 Onchocerca flexuosa           AATTTTTT--ATTTTTTTTT-GAAATTGGAAAAGAAAGTAATT
MK391038.1 Onchocerca flexuosa           AATTTTTT--ATTTTTTTTT-GAAATTGGAAAAGAAAGTAATT
LT732683.1 Onchocerca flexuosa           AATTTTTT--ATTTTTTTTT-GAAATTGGAAAAGAAAGTAATT
LT732682.1 Onchocerca flexuosa           AATTTTTT--ATTTTTTTTT-GAAATTGGAAAAGAAAGTAATT
MK391037.1 Onchocerca flexuosa           AATTTTTT--ATTTTTTTTT-GAAATTGGAAAAGAAAGTAATT
MK391033.1 Onchocerca flexuosa           AATTTTTT--ATTTTTTTTT-GAAATTGGAAAAGAAAGTAATT
HQ214004.1 Onchocerca flexuosa           AATTTTTT--ATTTTTTTTT-GAAATTGGAAAAGAAAGTAATT
MK391034.1 Onchocerca flexuosa           AATTTTTT--ATTTTTTTTT-GAAATTGGAAAAGAAAGTAATT
MK391043.1 Onchocerca flexuosa           AATTTTTT--ATTTTTTTTT-GAAATTGGAAAAGAAAGTAATT
AP017692.1 Onchocerca flexuosa           AATTTTTT--ATTTTTTTTT-GAAATTGGAAAAGAAAGTAATT
AB972362.1 Onchocerca takaokai           AATTTTTT--ATTTTTTTTT-GAAATTGGAAAAGAAAGTAATT
AB972364.1 Onchocerca takaokai           AATTTTTT--ATTTTTTTTT-GAAATTGGAAAAGAAAGTAATT
AB972363.1 Onchocerca takaokai           AATTTTTT--ATTTTTTTTT-GAAATTGGAAAAGAAAGTAATT
MH049488.1 Onchocerca sp. A AL           AATTTTTT--ATTTTTTTTT-GAAATTGGAAAAGAAAGTAATT
AM779811.1 Onchocerca suzukii            AATTTTTT--ATTTTTTTTT-GAAATTGGAAAAGAAAGTAATT
AM779812.1 Onchocerca suzukii            AATTTTTT--ATTTTTTTTT-GAAATTGGAAAAGAAAGTAATT
AB518879.1 Onchocerca sp. type           AATTTTTT--ATTTTTTTTT-GAAATTGGAAAAGAAAGTAATT
AB518877.1 Onchocerca sp. type           AATTTTTT--ATTTTTTTTTTGAAATTGGAAAAGAAAGTAATT
AB518878.1 Onchocerca sp. type           AATTTTTT--ATTTTTTTTTTGAAATTGGAAAAGAAAGTAATT
KC167341.1 Onchocerca ramachan           GATTTTTT--ATTTTTTTTT-GAAATTGGAAAAGAAAGTAATT
KC167340.1 Onchocerca ramachan           GATTTTTT--ATTTTTTTTT-GAAATTGGAAAAGAAAGTAATT
JX075208.1 Onchocerca cerviped           AATTTTTT--ATTTTTTTTT-GAAATTGGAAAAGAAAGTAATT
JX075207.1 Onchocerca cerviped           AATTTTTT--ATTTTTTTTT-GAAATTGGAAAAGAAAGTAATT
KP760345.2 Onchocerca armillat           AATTTTTT--ATTTTTTTTT-GAAATTGGAAAAGAAAGTAATT
KX853314.1 Onchocerca armillat           AATTTTTT--ATTTTTTTTT-GAAATTGGAAAAGAAAGTAATT
JN863696.1 Onchocerca lupi iso           AATTTTTT--ATTTTTTTTT-GAAATTGGAAAAGAAAGTAATT
GU365879.1 Onchocerca lupi 12S           AATTTTTT--ATTTTTTTTT-GAAATTGGAAAAGAAAGTAATT
AY462913.1 Onchocerca gibsoni            AATTTTTT--ATTTTTTTTT-GAAATTGGAAAAGAAAGTAATT
AY462912.1 Onchocerca gibsoni            AATTTTTT--ATTTTTTTTT-GAAATTGGAAAAGAAAGTAATT
MN985128.1 Onchocerca fasciata           AATTTTTT--ATTTTTTTTTTGAAATTGGAAAAGAAAGTAATT
MN985127.1 Onchocerca fasciata           AATTTTTT--ATTTTTTTTTTGAAATTGGAAAAGAAAGTAATT
JX075213.1 Onchocerca sp. QSM-           AATTTTTT--ATTTTTTTTT-GAAATTGGAAAAGAAAGTAATT
JX075215.1 Onchocerca sp. QSM-           AATTTTTT--ATTTTTTTTT-GAAATTGGAAAAGAAAGTAATT
JX075211.1 Onchocerca sp. QSM-           AATTTTTT--ATTTTTTTTT-GAAATTGGAAAAGAAAGTAATT
AY462926.1 Onchocerca linealis           AATTTTTT--ATTTTTTTTTTGAAATTGGAAAAGAAAGTAATT
AY462927.1 Onchocerca linealis           AATTTTTT--ATTTTTTTTTTGAAATTGGAAAAGAAAGTAATT
AY462924.1 Onchocerca linealis           AATTTTTT--ATTTTTTTTTTGAAATTGGAAAAGAAAGTAATT
AY462923.1 Onchocerca gutturos           AATTTTTTT-ATTTTTTTTTTGAAATTGGAAAAGAAAGTAATT
KX181290.2 Onchocerca ochengi            AGTTTTTT--ATTTTTTTTT-GAAATTGGAAAAGAAAGTAATT
KC167337.1 Onchocerca sp. 'Sii           AGTTTTTT--ATTTTTTTTT-GAAATTGGAAAAGAAAGTAATT
KC167338.1 Onchocerca sp. 'Sii           AGTTTTTT--ATTTTTTTTT-GAAATTGGAAAAGAAAGTAATT
KC167333.1 Onchocerca ochengi            AGTTTTTT--ATTTTTTTTT-GAAATTGGAAAAGAAAGTAATT
KC167334.1 Onchocerca ochengi            AGTTTTTT--ATTTTTTTTT-GAAATTGGAAAAGAAAGTAATT
AY462914.1 Onchocerca ochengi            AGTTTTTT--ATTTTTTTTT-GAAATTGGAAAAGAAAGTAATT
AY462915.1 Onchocerca ochengi            AGTTTTTT--ATTTTTTTTT-GAAATTGGAAAAGAAAGTAATT
FM206484.1 Onchocerca ochengi            AGTTTTTT--ATTTTTTTTT-GAAATTGGAAAAGAAAGTAATT
AY462916.1 Onchocerca ochengi            AGTTTTTT--ATTTTTTTTT-GAAATTGGAAAAGAAAGTAATT
AY462917.1 Onchocerca ochengi            AGTTTTTT--ATTTTTTTTT-GAAATTGGAAAGGAAAGTAATT
AP017694.1 Onchocerca ochengi            AGTTTTTT--ATTTTTTTTT-GAAATTGGAAAAGAAAGTAATT
NC_031891.2 Onchocerca ochengi           AGTTTTTT--ATTTTTTTTT-GAAATTGGAAAAGAAAGTAATT
KX181289.2 Onchocerca ochengi            AGTTTTTT--ATTTTTTTTT-GAAATTGGAAAAGAAAGTAATT
AP017693.1 Onchocerca ochengi            AGTTTTTT--ATTTTTTTTT-GAAATTGGAAAAGAAAGTAATT
AP017695.1 Onchocerca volvulus           AGTTTTTT--ATTTTTTTTT-GAAATTGGAAAAGAAAGTAATT
AF015193.1 Onchocerca volvulus           AGTTTTTT--ATTTTTTTTT-GAAATTGGAAAAGAAAGTAATT
AY462920.1 Onchocerca volvulus           AGTTTTTT--ATTTTTTTTT-GAAATTGGAAAAGAAAGTAATT
KT599912.1 Onchocerca volvulus           AGTTTTTT--ATTTTTTTTT-GAAATTGGAAAAGAAAGTAATT
KC167339.1 Onchocerca volvulus           AGTTTTTT--ATTTTTTTTT-GAAATTGGAAAAGAAAGTAATT
```

Supplementary information 2. Alignment COI – Nucleotide alignment of the COI gene used in the phylogenetic analysis represented in Fig 3.

```
                                            10        20        30        40        50        60
                                   ....|....|....|....|....|....|....|....|....|....|....|....|
MN564742.1 Dirofilaria sp. 'ho     TTGAATATTGCCTTTGATATTAGGAGCTCCTGAAATAGCTTTTCCTCGTGTTAATGCTTT
MF695085.1 Dirofilaria repens     TTGGATGTTGCCTTTGATATTGGGGGCTCCTGAGATGGCGTTTCCTCGTGTTAATGCTTT
AB518693.1 Onchocerca sp. wild    TTGGATATTGCCTTTGATGTTAGGTGCTCCTGAAATAGCATTTCCTCGTGTCAATGCTTT
AB518694.1 Onchocerca sp. wild    TTGGATATTGCCTTTGATGTTAGGTGCTCCCGAAATAGCATTTCCTCGTGTCAATGCTTT
AB518874.1 Onchocerca dewittei    TTGAATATTACCTTTGATGTTAGGTGCTCCAGAGATAGCTTTTCCTCGTGTTAATGCTTT
AB518690.1 Onchocerca dewittei    TTGAATATTACCTTTGATGTTAGGTGCTCCAGAGATAGCTTTTCCTCGTGTTAATGCTTT
AB518692.1 Onchocerca dewittei    TTGAATATTACCTTTGATGTTGGGTGCTCCAGAGATAGCTTTTCCTCGTGTTAATGCTTT
AB518689.1 Onchocerca dewittei    TTGAATATTACCTTTGATGTTAGGTGCTCCAGAGATAGCTTTTCCTCGTGTTAATGCTTT
AM749268.1 Onchocerca eberhard    NNNNNNNNTACCTTTGATGTTGGGGGCCCCGGAAATAGCGTTTCCTCGTGTTAATGCTTT
LC318284.1 Onchocerca flexuosa    TTGGATATTACCTTTGATATTGGGGGCTCCTGAGATGGCTTTTCCTCGTGTGAATGCTTT
AM749269.1 Onchocerca skrjabin    NNNNNNNNTACCTTTGATATTGGGGGCTCCTGAGATAGCTTTTCCTCGTGTTAATGCTTT
KX898458.1 Onchocerca boehmi c    NNNNNNNNNCCCTTTAATATTGGGAGCTCCTGAAATGGCTTTTCCTCGTGTTAACGCTTT
MG029461.1 Onchocerca sp. ALDA    TTGGATGTTACCTTTGATGTTAGGAGCTCCTGAGATGGCTTTTCCTCGTGTAAACGCTTT
MG029460.1 Onchocerca sp. ALDA    TTGGATGTTACCTTTGATGTTAGGAGCTCCTGAGATGGCTTTTCCTCGTGTAAACGCTTT
MG677940.1 Onchocerca lupi cyt    TTGGATGTTGCCTTTGATGTTGGGGGCTCCGGAGATGGCTTTTCCTCGAATTAACGCTTT
EF521410.1 Onchocerca lupi fro    TTGGATGTTGCCTTTGATGTTGGGGGCTCCGGAGATGGCTTTTCCTCGAATTAACGCTTT
HQ207644.1 Onchocerca lupi fro    TTGGATGTTGCCTTTGATGTTGGGGGCTCCAGAGATGGCTTTTCCTCGAATTAACGCTTT
KC686702.1 Onchocerca lupi cyt    TTGGATGTTGCCTTTGATGTTGGGGGCTCCAGAGATGGCTTTTCCTCGAATTAACGCTTT
AJ415417.1 Onchocerca lupi mit    TTGGATGTTGCCTTTGATGTTGGGGGCTCCAGAGATGGCTTTTCCTCGAATTAACGCTTT
KP283476.1 Onchocerca lupi iso    TTGGATGTTGCCTTTGATGTTGGGGGCTCCAGAGATGGCTTTTCCTCGAATTAACGCTTT
KP283477.1 Onchocerca lupi iso    TTGGATGTTGCCTTTGATGTTGGGGGCTCCAGAGATGGCTTTTCCTCGAATTAACGCTTT
JX080030.1 Onchocerca lupi iso    TTGGATGTTGCCTTTGATGTTGGGGGCTCCAGAGATGGCTTTTCCTCGAATTAACGCTTT
JX183106.1 Onchocerca lupi cyt    TTGGATGTTGCCTTTGATGTTGGGGGCTCCAGAGATGGCTTTTCCTCGAATTAACGCTTT
KX853328.1 Onchocerca lupi vou    NNNNNNNNNNNNNNNNNNNNNNNNNNNNNNNNNNNNNNCTTTTCCTCGAATTAACGCTTT
JX080029.1 Onchocerca lupi iso    TTGGATGTTGCCTTTGATGTTGGGGGCTCCAGAGATGGCTTTTCCTCGAATTAACGCTTT
EF521409.1 Onchocerca lupi fro    TTGGATGTTGCCTTTGATGTTGGGGGCTCCAGAGATGGCTTTTCCTCGAATTAACGCTTT
AJ271616.1 Onchocerca gibsoni     TTGG-TGTTACCTTTGATATTAGGGGCTCCTGAGATAGCTTTTCCTCGAGTTAATGCTTT
AJ271617.1 Onchocerca gutturos    NTTTTTGTTACCTTTGATATTGGGGGCTCCTGAGATGGCTTTTCCTCGGGTTAATGCTTT
KP760201.1 Onchocerca gutturos    NNNNNNNNNNNNNNNNNNNNNNNNNNNNNNNNNNNNNNCTTTTCCTCGGGTTAATGCTTT
KX853325.1 Onchocerca lienalis    NNNNNNNNNNNNNNNNNNNNNNNNNNNNNNNNNNNNNNNATTTCCTCGGGTTAATGCCTT
KT001213.1 Onchocerca jakutens    TTGGATATTGCCTTTGATGTTGGGGGCTCCCGAGATAGCGTTTCCTCGGGTAAATGCTTT
JQ316672.1 Onchocerca fasciata    TTGGATGTTGCCTTTAATGTTGGGGGCTCCAGAGATGGCTTTTCCTCGGGTGAATGCGTT
MG188678.1 Onchocerca fasciata    TTGGATGTTGCCTTTAATGTTGGGGGCTCCAGAGATGGCTTTTCCTCGGGTGAATGCGTT
MT002817.1 Onchocerca fasciata    TTGGATGTTGCCTTTAATGTTGGGGGCTCCAGAGATGGCTTTTCCTCGGGTGAATGCGTT
KC167355.1 Onchocerca volvulus    TTGGATGTTGCCTTTGATGTTGGGGGCTCCTGAGATGGCGTTTCCTCGGGTAAATGCTTT
MH190075.1 Onchocerca volvulus    TTGGATGTTGCCTTTGATGTTGGGGGCTCCTGAGATGGCGTTTCCTCGGGTAAATGCTTT
KC167354.1 Onchocerca sp. 'Sii    TTGGATGTTGCCTTTGATGTTAGGGGCTCCTGAGATGGCGTTTCCTCGGGTAAATGCTTT
KC167352.1 Onchocerca sp. 'Sii    TTGGATGTTGCCTTTGATGTTAGGGGCTCCTGAGATGGCGTTTCCTCGGGTAAATGCTTT
KC167353.1 Onchocerca sp. 'Sii    TTGGATGTTGCCTTTGATGTTAGGGGCTCCTGAGATGGCGTTTCCTCGAGTAAATGCTTT
AJ271618.1 Onchocerca ochengi     NNNGATGTTGCCTTTAAATTTAGGGGCTCCTGAGATGGCGTTTCCTCGGGTAAATGCTTT
KC167358.1 Onchocerca ochengi     TTGGATGTTGCCTTTAATATTAGGGGCTCCTGAGATGGCGTTTCCTCGGGTAAATGCTTT
KC167351.1 Onchocerca ochengi     TTGGATGTTGCCTTTAATATTAGGGGCTCCTGAGATAGCGTTTCCTCGGGTAAATGCTTT
NC_031891.2 Onchocerca ochengi    TTGGATGTTGCCTTTAATATTAGGGGCTCCTGAGATGGCGTTTCCTCGGGTAAATGCTTT
KC167350.1 Onchocerca ochengi     TTGGATGTTGCCTTTAATATTAGGGGCTCCTGAGATGGCGTTTCCTCGGGTAAATGCTTT
KX181289.2 Onchocerca ochengi     TTGGATGTTGCCTTTAATATTAGGGGCTCCTGAGATGGCGTTTCCTCGGGTAAATGCTTT

                                            70        80        90       100       110       120
                                   ....|....|....|....|....|....|....|....|....|....|....|....|
MN564742.1 Dirofilaria sp. 'ho     GTCTTTTTGATTTACTTTTGTTGCTTTATTGATGGTTTATCAGTCTTTTTTTATTGGTGG
MF695085.1 Dirofilaria repens     GTCTTTTTGGTTTACTTTTGTTGCTTTGTTAATGGTTTATCAATCTTTTTTTATTGGTGG
AB518693.1 Onchocerca sp. wild    GTCTTTTTGGTTTACTTTTTCTGCTTTGTTAATAGTTTATCAGTCTTTTTTTATTGGTGG
AB518694.1 Onchocerca sp. wild    GTCTTTTTGGTTTACTTTTTCTGCTTTGTTAATAGTTTATCAGTCTTTTTTTATTGGTGG
AB518874.1 Onchocerca dewittei    ATCTTTTTGATTTACTTTTTCTGCATTATTAATGGTATATCAATCTTTTTTTATTGGCGG
AB518690.1 Onchocerca dewittei    ATCTTTTTGATTTACTTTTTCTGCATTATTAATGGTATATCAATCTTTTTTTATTGGCGG
AB518692.1 Onchocerca dewittei    ATCTTTTTGATTTACTTTTTCTGCATTATTAATGGTATATCAATCTTTTTTTATTGGCGG
AB518689.1 Onchocerca dewittei    ATCTTTTTGATTTACTTTTTCTGCATTATTAATGGTATATCAATCTTTTTTTATTGGCGG
AM749268.1 Onchocerca eberhard    ATCTTTTTGATTTACTTTTGTGGCTTTGTTAATGGTTTATCAGTCTCTTTTTATTGGGGG
LC318284.1 Onchocerca flexuosa    GTCTTTTTGATTTACTTTTGTGGCTTTGTTGATAGTTTATCAATCTTTTTTTATTGGGGG
AM749269.1 Onchocerca skrjabin    ATCTTTTTGGTTTACTTTTGTGGCTTTATTAATGGTTTATCAGTCTTTTTTTATTGGAGG
KX898458.1 Onchocerca boehmi c    ATCTTTTTGGTTTACTTTTGTTGCTTTATTGATAGTGTATCAGTCTTTTTTTATTGGGGG
MG029461.1 Onchocerca sp. ALDA    GTCTTTTTGATTTACTTTTGTGGCGTTAATTATGGTTTATCAGTCTTTTTTTATTGGAGG
MG029460.1 Onchocerca sp. ALDA    GTCTTTTTGATTTACTTTTGTGGCGTTAATTATGGTTTATCAGTCTTTTTTTATTGGAGG
MG677940.1 Onchocerca lupi cyt    GTCTTTTTGGTTTACTTTTGTATCTTTGTTGATGGTTTATCAGTCTTTTTTTATTGGAGG
EF521410.1 Onchocerca lupi fro    GTCTTTTTGGTTTACTTTTGTATCTTTGTTGATGGTTTATCAGTCTTTTTTTATTGGAGG
HQ207644.1 Onchocerca lupi fro    GTCTTTTTGGTTTACTTTTGTATCTTTGTTGATGGTTTATCAGTCTTTTTTTATTGGAGG
KC686702.1 Onchocerca lupi cyt    GTCTTTTTGGTTTACTTTTGTATCTTTGTTGATGGTTTATCAGTCTTTTTTTATTGGAGG
AJ415417.1 Onchocerca lupi mit    GTCTTTTTGGTTTACTTTTGTATCTTTGTTGATGGTTTATCAGTCTTTTTTTATTGGAGG
KP283476.1 Onchocerca lupi iso    GTCTTTTTGGTTTACTTTTGTATCTTTGTTGATGGTTTATCAGTCTTTTTTTATTGGAGG
KP283477.1 Onchocerca lupi iso    GTCTTTTTGGTTTACTTTTGTATCTTTGTTGATGGTTTATCAGTCTTTTTTTATTGGAGG
JX080030.1 Onchocerca lupi iso    GTCTTTTTGGTTTACTTTTGTATCTTTGTTGATGGTTTATCAGTCTTTTTTTATTGGAGG
JX183106.1 Onchocerca lupi cyt    GTCTTTTTGGTTTACTTTTGTATCTTTGTTGATGGTTTATCAGTCTTTTTTTATTGGAGG
KX853328.1 Onchocerca lupi vou    GTCTTTTTGGTTTACTTTTGTATCTTTGTTGATGGTTTATCAGTCTTTTTTTATTGGAGG
JX080029.1 Onchocerca lupi iso    GTCTTTTTGGTTTACTTTTGTATCTTTGTTGATGGTTTATCAGTCTTTTTTTATTGGAGG
EF521409.1 Onchocerca lupi fro    GTCTTTTTGGTTTACTTTTGTATCTTTGTTGATGGTTTATCAGTCTTTTTTTATTGGAGG
AJ271616.1 Onchocerca gibsoni     GTCTTTTTGGTTTACTTTTGTAGCTTTGTTGATAGTTTATCAGTCTTTTTTTATTGGAGG
AJ271617.1 Onchocerca gutturos    GTCTTTTTGGTTTACTTTTGTAGCTTTGTTGATGGTTTATCAGTCTTTTTTTATTGGTGG
```

```
KP760201.1 Onchocerca gutturos  GTCTTTTTGGTTTACTTTTGTAGCTTTGTTGATGGTTTATCAGTCTTTTTTTATTGGTGG
KX853325.1 Onchocerca lienalis  GTCTTTTTGGTTTACTTTTGTGGCTTTGTTGATGGTTTATCAATCTTTTTTTATTGGAGG
KT001213.1 Onchocerca jakutens  GTCTTTTTGGTTTACTTTTGTGGCTTTATTAATGGTTTATCAGTCTTTTTTTATTGGTGG
JQ316672.1 Onchocerca fasciata  GTCTTTTTGGTTTACTTTTGTGGCTTTGTTGATAGTTTATCAATCTTTTTTTATTGGAGG
MG188678.1 Onchocerca fasciata  GTCTTTTTGGTTTACTTTTGTGGCTTTGTTGATAGTTTATCAATCTTTTTTTATTGGAGG
MT002817.1 Onchocerca fasciata  GTCTTTTTGGTTTACTTTTGTGGCTTTGTTGATAGTTTATCAATCTTTTTTTATTGGAGG
KC167355.1 Onchocerca volvulus  ATCTTTTTGGTTTACTTTTGTGGCTTTATTGATAGTTTATCAGTCTTTTTTTATTGGGGG
MH190075.1 Onchocerca volvulus  ATCTTTTTGGTTTACTTTTGTGGCTTTATTGATAGTTTATCAGTCTTTTTTTATTGGGGG
KC167354.1 Onchocerca sp. 'Sii  GTCTTTTTGATTTACTTTTGTGGCTTTGTTGATAGTTTATCAGTCTTTTTTTATCGGAGG
KC167352.1 Onchocerca sp. 'Sii  GTCTTTTTGATTTACTTTTGTGGCTTTGTTGATAGTTTATCAGTCTTTTTTTATCGGAGG
KC167353.1 Onchocerca sp. 'Sii  GTCTTTTTGATTTACTTTTGTGGCTTTGTTGATAGTTTATCAGTCTTTTTTTATCGGAGG
AJ271618.1 Onchocerca ochengi   ATCTTTTTGATTTACTTTTGTGGCTTTGTTGATAGTTTATCAGTCTTTTTTTATTGGGGG
KC167358.1 Onchocerca ochengi   ATCTTTTTGATTTACTTTTGTGGCTTTGTTGATAGTTTATCAGTCTTTTTTTATTGGGGG
KC167351.1 Onchocerca ochengi   ATCTTTTTGATTTACTTTTGTGGCTTTGTTGATAGTTTATCAGTCTTTTTTTATTGGGGG
NC_031891.2 Onchocerca ochengi  ATCTTTTTGATTTACTTTTGTGGCTTTGTTGATAGTTTATCAGTCTTTTTTTATTGGGGG
KC167350.1 Onchocerca ochengi   ATCTTTTTGATTTACTTTTGTGGCTTTGTTGATAGTTTATCAGTCTTTTTTTATTGGGGG
KX181289.2 Onchocerca ochengi   ATCTTTTTGATTTACTTTTGTGGCTTTGTTGATAGTTTATCAGTCTTTTTTTATTGGGGG

                                         130       140       150       160       170       180
                                ....|....|....|....|....|....|....|....|....|....|....|....|
MN564742.1 Dirofilaria sp. 'ho  TGGACCTGGTAGTAGTTGAACTTTTTATCCTCCTTTAAGTGTTGATGGTCAACCTGAGTT
MF695085.1 Dirofilaria repens   TGGACCTGGTAGTAGTTGAACTTTTTATCCTCCTTTAAGTGTTGATGGTCAACCTGAATT
AB518693.1 Onchocerca sp. wild  TGGTCCTGGAAGTAGTTGAACTTTTTATCCCCCTCTTAGAATTGAAGGTCAGCCAGAATT
AB518694.1 Onchocerca sp. wild  TGGTCCTGGAAGTAGTTGAACTTTTTATCCCCCTCTTAGAATTGAAGGTCAGCCAGAATT
AB518874.1 Onchocerca dewittei  TGGTCCTGGTAGTAGTTGAACTTTTTATCCTCCTTTAAGAGTTGAAGGTCAGCCTGAATT
AB518690.1 Onchocerca dewittei  TGGTCCTGGTAGTAGTTGAACTTTTTATCCTCCTTTAAGAGTTGAAGGTCAGCCTGAATT
AB518692.1 Onchocerca dewittei  TGGTCCTGGTAGTAGTTGAACTTTTTATCCTCCTTTAAGAGTTGAAGGTCAGCCTGAATT
AB518689.1 Onchocerca dewittei  TGGTCCTGGTAGTAGTTGAACTTTTTATCCTCCTTTAAGAGTTGAAGGTCAGCCTGAATT
AM749268.1 Onchocerca eberhard  TGGCCCTGGTAGTAGTTGAACTTTTTATCCTCCTTTAAGAGTTGAAGGTCAACCGGAGCT
LC318284.1 Onchocerca flexuosa  TGGTCCAGGTAGTAGTTGAACTTTTTACCCTCCTTTAAGAGTAGAAGGTCAGCCAGAGTT
AM749269.1 Onchocerca skrjabin  TGGTCCTGGTAGTAGTTGGACTTTTTATCCTCCTTTAAGAGTTGAGGGTCAGCCAGAATT
KX898458.1 Onchocerca boehmi c  TGGTCCTGGGAGTAGTTGGACTTTTTATCCTCCTCTCAGAGTTGAAGGTCAGCCGGAGTT
MG029461.1 Onchocerca sp. ALDA  TGGCCCTGGTAGTAGTTGGACTTTTTATCCTCCGCTAAGAACTGAGGGTCAACCTGAGTT
MG029460.1 Onchocerca sp. ALDA  TGGCCCTGGTAGTAGTTGGACTTTTTATCCTCCGCTAAGAACTGAGGGTCAACCTGAGTT
MG677940.1 Onchocerca lupi cyt  TGGTCCTGGTAGTAGTTGGACTTTTTATCCTCCTCTTAGAGTAGAGGGTCAGCCTGAGTT
EF521410.1 Onchocerca lupi fro  TGGTCCTGGTAGTAGTTGGACTTTTTATCCTCCTCTTAGAGTAGAGGGTCAGCCTGAGTT
HQ207644.1 Onchocerca lupi fro  TGGTCCTGGTAGTAGTTGAACTTTTTATCCTCCTCTTAGAGTAGAGGGTCAGCCTGAGTT
KC686702.1 Onchocerca lupi cyt  TGGTCCTGGTAGTAGTTGAACTTTTTATCCTCCTCTTAGAGTAGAGGGTCAGCCTGAGTT
AJ415417.1 Onchocerca lupi mit  CGGTCCTGGTAGTAGTTGAACTTTTTATCCTCCTCTTAGAGTAGAGGGTCAGCCTGAGTT
KP283476.1 Onchocerca lupi iso  CGGTCCTGGTAGTAGTTGAACTTTTTATCCTCCTCTTAGAGTAGAGGGTCAGCCTGAGTT
KP283477.1 Onchocerca lupi iso  CGGTCCTGGTAGTAGTTGAACTTTTTATCCTCCTCTTAGAGTAGAGGGTCAGCCTGAGTT
JX080030.1 Onchocerca lupi iso  CGGTCCTGGTAGTAGTTGAACTTTTTATCCTCCTCTTAGAGTAGAGGGTCAGCCTGAGTT
JX183106.1 Onchocerca lupi cyt  CGGTCCTGGTAGTAGTTGAACTTTTTATCCTCCTCTTAGAGTAGAGGGTCAGCCTGAGTT
KX853328.1 Onchocerca lupi vou  TGGTCCTGGTAGTAGTTGGACTTTTTATCCTCCTCTTAGAGTAGAGGGTCAGCCTGAGTT
JX080029.1 Onchocerca lupi iso  CGGTCCTGGTAGTAGTTGAACTTTTTATCCTCCTCTTAGAGTAGAGGGTCAGCCTGAGTT
EF521409.1 Onchocerca lupi fro  CGGTCCTGGTAGTAGTTGAACTTTTTATCCTCCTCTTAGAGTAGAGGGTCAGCCTGAGTT
AJ271616.1 Onchocerca gibsoni   AGGACCTGGAAGAAGTTGGACTTTTTATCCTCCTCTTAGGGTTGAAGGTCAACCAGAATT
AJ271617.1 Onchocerca gutturos  TGGTCCTGGTAGAAGTTGGACTTTTTATCCTCCTCTTAGGGTTGAGGGTCAACCGGAGTT
KP760201.1 Onchocerca gutturos  TGGTCCTGGTAGAAGTTGGACTTTTTATCCTCCTCTTAGGGTTGAGGGTCAACCGGAGTT
KX853325.1 Onchocerca lienalis  TGGCCCTGGTAGAAGCTGGACTTTTTATCCTCCTCTTAGGGTTGAGGGTCAACCTGAGTT
KT001213.1 Onchocerca jakutens  TGGTCCTGGTAGAAGATGGACTTTTTATCCTCCTTTAAGGGTTGAGGGTCAGCCGGAGTT
JQ316672.1 Onchocerca fasciata  TGGCCCTGGTAGAAGTTGGACTTTTTATCCTCCTTTGAGGGTTGAGGGTCAACCGGAATT
MG188678.1 Onchocerca fasciata  TGGCCCTGGTAGAAGTTGGACTTTTTATCCTCCTTTGAGGGTTGAGGGTCAACCGGAATT
MT002817.1 Onchocerca fasciata  TGGCCCTGGTAGAAGTTGGACTTTTTATCCTCCTTTGAGGGTTGAGGGTCAACCGGAATT
KC167355.1 Onchocerca volvulus  TGGTCCTGGTAGGAGTTGGACTTTTTATCCTCCTCTTAGGGTTGAAGGTCAACCAGAATT
MH190075.1 Onchocerca volvulus  TGGTCCTGGTAGGAGTTGGACTTTTTATCCTCCTCTTAGGGTTGAAGGTCAACCAGAATT
KC167354.1 Onchocerca sp. 'Sii  TGGTCCTGGTAGAAGTTGGACTTTTTATCCTCCTCTTAGGGTTGAAGGCCAACCAGAATT
KC167352.1 Onchocerca sp. 'Sii  TGGTCCTGGTAGAAGTTGGACTTTTTATCCTCCTCTTAGGGTTGAAGGCCAACCAGAATT
KC167353.1 Onchocerca sp. 'Sii  TGGTCCTGGTAGAAGTTGGACTTTTTATCCTCCTCTTAGGGTTGAAGGCCAACCAGAATT
AJ271618.1 Onchocerca ochengi   TGGCCCTGGTAGAAGTTGGACTTTTTATCCTCCTCTTAGGGTTGAAGGTCAACCAGAATT
KC167358.1 Onchocerca ochengi   TGGCCCTGGTAGAAGTTGGACTTTTTATCCTCCTCTTAGGGTTGAAGGTCAACCAGAATT
KC167351.1 Onchocerca ochengi   TGGCCCTGGTAGAAGTTGGACTTTTTATCCTCCTCTTAGGGTTGAAGGTCAACCAGAATT
NC_031891.2 Onchocerca ochengi  TGGCCCTGGTAGAAGTTGGACTTTTTATCCTCCTCTTAGGGTTGAAGGTCAACCAGAATT
KC167350.1 Onchocerca ochengi   TGGCCCTGGTAGAAGTTGGACTTTTTATCCTCCTCTTAGGGTTGAAGGTCAACCAGAATT
KX181289.2 Onchocerca ochengi   TGGCCCTGGTAGAAGTTGGACTTTTTATCCTCCTCTTAGGGTTGAAGGTCAACCAGAATT

                                         190       200       210       220       230       240
                                ....|....|....|....|....|....|....|....|....|....|....|....|
MN564742.1 Dirofilaria sp. 'ho  ATCTTTAGATAGTATAATTTTGGGATTACATACTGTAGGTATTGGTTCTTTGTTGGGTGC
MF695085.1 Dirofilaria repens   ATCTTTGGATAGTATAATTTTGGGTTTACATACTGTAGGTATTGGTTCTTTGTTGGGTGC
AB518693.1 Onchocerca sp. wild  GTCTTTAGATACTATGATTTTAGGTTTGCATACTGTAGGTATTGGTTCTTTATTGGGTGC
AB518694.1 Onchocerca sp. wild  GTCTTTAGATACTATGATTTTAGGTTTGCATACTGTAGGTATTGGTTCTTTATTGGGTGC
AB518874.1 Onchocerca dewittei  GTCTTTAGATACTATAATTTTGGGTTTGCATACTGTAGGTATTGGTTCTTTGTTAGGTGC
AB518690.1 Onchocerca dewittei  GTCTTTAGATACTATAATTTTGGGTTTACATACTGTAGGTATTGGTTCTTTGTTAGGTGC
AB518692.1 Onchocerca dewittei  GTCTTTAGATACTATAATTTTGGGTTTGCATACTGTAGGTATTGGTTCTTTGTTAGGTGC
AB518689.1 Onchocerca dewittei  GTCTTTAGATACTATAATTTTGGGTTTGCATACTGTAGGTATTGGTTCTTTGTTAGGTGC
AM749268.1 Onchocerca eberhard  GTCTTTAGATACTATGATTTTAGGTTTACATACTGTTGGTATTGGTTCTTTGTTAGGTGC
LC318284.1 Onchocerca flexuosa  GTCTTTGGATACCATGATTTTAGGCTTGCATACTGTTGGGATTGGTTCTTTGTTAGGTGC
```

```
AM749269.1 Onchocerca skrjabin  ATCATTGGATACTATAATTTTGGGTTTGCATACTGTTGGTATTGGTTCTTTGTTAGGTGC
KX898458.1 Onchocerca boehmi c  GTCTTTAGATACTATGATTTTGGGTTTGCATACTGTTGGTATTGGTTCTTTGTTGGGTGC
MG029461.1 Onchocerca sp. ALDA  GTCTTTGGATACAATAATTTTGGGATTACATACTGTAGGTATTGGTTCTTTGTTGGGTGC
MG029460.1 Onchocerca sp. ALDA  GTCTTTGGATACAATAATTTTGGGATTACATACTGTAGGTATTGGTTCTTTGTTGGGTGC
MG677940.1 Onchocerca lupi cyt  ATCTTTGGATACTATAGTTTTAGGTTTGCATACTGTTGGTATTGGTTCTTTGTTGGGTGC
EF521410.1 Onchocerca lupi fro  ATCTTTGGATACTATAGTTTTAGGTTTGCATACTGTTGGTATTGGTTCTTTGTTGGGTGC
HQ207644.1 Onchocerca lupi fro  ATCTTTGGATACTATAGTTTTGGGTTTGCATACTGTTGGTGTTGGTTCTTTGTTGGGTGC
KC686702.1 Onchocerca lupi cyt  ATCTTTGGATACTATAGTTTTGGGTTTGCATACTGTTGGTGTTGGTTCTTTGTTGGGTGC
AJ415417.1 Onchocerca lupi mit  ATCTTTGGATACTATAGTTTTGGGTTTGCATACTGTTGGTGTTGGTTCTTTGTTGGGTGC
KP283476.1 Onchocerca lupi iso  ATCTTTGGATACTATAGTTTTGGGTTTGCATACTGTTGGTGTTGGTTCTTTGTTGGGTGC
KP283477.1 Onchocerca lupi iso  ATCTTTGGATACTATAGTTTTGGGTTTGCATACTGTTGGTGTTGGTTCTTTGTTGGGTGC
JX080030.1 Onchocerca lupi iso  ATCTTTGGATACTATAGTTTTGGGTTTGCATACTGTTGGTGTTGGTTCTTTGTTGGGTGC
JX183106.1 Onchocerca lupi cyt  ATCTTTGGATACTATAGTTTTGGGTTTGCATACTGTTGGTGTTGGTTCTTTGTTGGGTGC
KX853328.1 Onchocerca lupi vou  ATCTTTGGATACTATAGTTTTAGGTTTGCATACTGTTGGTATTGGTTCTTTGTTGGGTGC
JX080029.1 Onchocerca lupi iso  ATCTTTGGATACTATAGTTTTGGGTTTGCATACTGTTGGTGTTGGTTCTTTGTTGGGTGC
EF521409.1 Onchocerca lupi fro  ATCTTTGGATACTATAGTTTTGGGTTTGCATACTGTTGGTGTTGGTTCTTTGTTGGGTGC
AJ271616.1 Onchocerca gibsoni   GTCTTTGGATACTATGATTTTGGGTTTGCATACTGTAGGTATTGGTTCTTTGTTGGGTGC
AJ271617.1 Onchocerca gutturos  GTCTTTGGATACTATAATTTTGGGTTTGCATACTGTAGGTATTGGTTCTTTGTTGGGTGC
KP760201.1 Onchocerca gutturos  GTCTTTGGATACTATAATTTTGGGTTTGCATACTGTGGGTATTGGTTCTTTGTTGGGTGC
KX853325.1 Onchocerca lienalis  GTCTTTGGATACTATGATTTTAGGTTTGCATACTGTTGGTATTGGTTCTTTGTTGGGTGC
KT001213.1 Onchocerca jakutens  ATCTCTGGATACTATAATTTTGGGTTTACATACTGTGGGTATTGGATCTTTATTGGGTGC
JQ316672.1 Onchocerca fasciata  GTCTTTGGATACTATGATTTTAGGTTTACATACTGTGGGTGTTGGTTCTTTGTTGGGTGC
MG188678.1 Onchocerca fasciata  GTCTTTGGATACTATGATTTTAGGTTTACATACTGTGGGTGTTGGTTCTTTGTTGGGTGC
MT002817.1 Onchocerca fasciata  GTCTTTGGATACTATGATTTTAGGTTTACATACTGTGGGTGTTGGTTCTTTGTTGGGTGC
KC167355.1 Onchocerca volvulus  GTCTTTAGATACTATGATTTTAGGTTTACATACTGTAGGAATTGGTTCTTTGTTGGGTGC
MH190075.1 Onchocerca volvulus  GTCTTTAGATACTATGATTTTAGGTTTACATACTGTAGGAATTGGTTCTTTGTTGGGTGC
KC167354.1 Onchocerca sp. 'Sii  GTCTTTGGATACTATGATTTTAGGTTTACATACTGTAGGAATTGGTTCTTTGTTGGGTGC
KC167352.1 Onchocerca sp. 'Sii  GTCTTTGGATACTATGATTTTAGGTTTACATACTGTAGGAATTGGTTCTTTGTTGGGTGC
KC167353.1 Onchocerca sp. 'Sii  GTCTTTGGATACTATGATTTTAGGTTTACATACTGTAGGAATTGGTTCTTTGTTGGGTGC
AJ271618.1 Onchocerca ochengi   GTCTTTAGATACTATGATTTTAGGTTTACATACTGTGGGAATTGGTTCTTTGTTGGGTGC
KC167358.1 Onchocerca ochengi   GTCTTTAGATACTATGATTTTAGGTTTACATACTGTGGGAATTGGTTCTTTGTTGGGTGC
KC167351.1 Onchocerca ochengi   GTCTTTAGATACTATGATTTTAGGTTTACATACTGTGGGAATTGGTTCTTTGTTGGGTGC
NC_031891.2 Onchocerca ochengi  GTCTTTAGATACTATGATTTTAGGTTTACATACTGTAGGAATTGGTTCTTTGTTGGGTGC
KC167350.1 Onchocerca ochengi   GTCTTTAGATACTATGATTTTAGGTTTACATACTGTAGGAATTGGTTCTTTGTTGGGTGC
KX181289.2 Onchocerca ochengi   GTCTTTAGATACTATGATTTTAGGTTTACATACTGTAGGAATTGGTTCTTTGTTGGGTGC

                                         250       260       270       280       290       300
                                ....|....|....|....|....|....|....|....|....|....|....|....|
MN564742.1 Dirofilaria sp. 'ho  TATTAATTTTATAGTTACTACTCAGAATATACGTTCTACTGCTGTTACTTTAGATCAAAT
MF695085.1 Dirofilaria repens   TATTAATTTTATGGTTACTACTCAGAATATGCGTTCTACTGCTGTTACTTTAGATCAGAT
AB518693.1 Onchocerca sp. wild  AATTAATTTTATGGTTACTACTCAAAATATGCGTTCTAGAGCTGTTACTTTGGATCAGAT
AB518694.1 Onchocerca sp. wild  AATTAATTTTATGGTTACTACTCAAAATATGCGTTCTAGAGCTGTTACTTTGGATCAGAT
AB518874.1 Onchocerca dewittei  TATTAATTTTATAGTTACTACTCAGAATATGCGTTCTACAGCTGTTACTTTAGATCAAAT
AB518690.1 Onchocerca dewittei  TATTAATTTTATAGTTACTACTCAGAATATGCGTTCTACAGCTGTTACTTTAGATCAAAT
AB518692.1 Onchocerca dewittei  TATTAATTTTATAGTTACTACTCAGAATATGCGTTCTACAGCTGTTACTTTAGATCAAAT
AB518689.1 Onchocerca dewittei  TATTAATTTTATAGTTACTACTCAGAATATGCGTTCTACAGCTGTTACTTTAGATCAAAT
AM749268.1 Onchocerca eberhard  TATTAATTTTATAGTTACTACTCAGAATATGCGTTCTACGGCTGTTACTTTGGATCAAAT
LC318284.1 Onchocerca flexuosa  TATTAATTTTATAGTTACTACTCAAAATATGCGTTCTACTGCTGTGACTTTGGATCAAAT
AM749269.1 Onchocerca skrjabin  TATTAATTTTATGGTTACTACTCAAAATATACGTTCTACAGCTGTGACTTTAGATCAAAT
KX898458.1 Onchocerca boehmi c  TATTAATTTTATAGTGACTACTCAAAATATGCGTTCTACGGCTGTTACTTTGGATCAAAT
MG029461.1 Onchocerca sp. ALDA  TATTAATTTTATAGTTACTACCCAAAATATGCGTTCTACTGCTGTGACTCTAGATCAGAT
MG029460.1 Onchocerca sp. ALDA  TATTAATTTTATAGTTACTACCCAAAATATGCGTTCTACTGCTGTGACTCTAGATCAGAT
MG677940.1 Onchocerca lupi cyt  TATTAATTTTATGGTTACTACTCAAAATATGCGTTCTACTGCTGTGACTTTAGACCAAAT
EF521410.1 Onchocerca lupi fro  TATTAATTTTATGGTTACTACTCAAAATATGCGTTCTACTGCTGTGACTTTAGACCAAAT
HQ207644.1 Onchocerca lupi fro  TATTAATTTTATGGTTACTACTCAAAATATGCGTTCTACTGCTGTGACTTTAGATCAAAT
KC686702.1 Onchocerca lupi cyt  TATTAATTTTATGGTTACTACTCAAAATATGCGTTCTACTGCTGTGACTTTAGATCAAAT
AJ415417.1 Onchocerca lupi mit  TATTAATTTTATGGTTACTACTCAAAATATGCGTTCTACTGCTGTGACTTTAGATCAAAT
KP283476.1 Onchocerca lupi iso  TATTAATTTTATGGTTACTACTCAAAATATGCGTTCTACTGCTGTGACTTTAGATCAAAT
KP283477.1 Onchocerca lupi iso  TATTAATTTTATGGTTACTACTCAAAATATGCGTTCTACTGCTGTGACTTTAGATCAAAT
JX080030.1 Onchocerca lupi iso  TATTAATTTTATGGTTACTACTCAAAATATGCGTTCTACTGCTGTGACTTTAGATCAAAT
JX183106.1 Onchocerca lupi cyt  TATTAATTTTATGGTTACTACTCAAAATATGCGTTCTACTGCTGTGACTTTAGATCAAAT
KX853328.1 Onchocerca lupi vou  TATTAATTTTATGGTTACTACTCAAAATATGCGTTCTACTGCTGTGACTTTAGACCAAAT
JX080029.1 Onchocerca lupi iso  TATTAATTTTATGGTTACTACTCAAAATATGCGTTCTACTGCTGTGACTTTAGATCAAAT
EF521409.1 Onchocerca lupi fro  TATTAATTTTATGGTTACTACTCAAAATATGCGTTCTACTGCTGTGACTTTAGATCAAAT
AJ271616.1 Onchocerca gibsoni   TATTAATTTTATGGTTACTACTCAAAATATGCGATCTACTGCTGTGACCTTGGATCAAAT
AJ271617.1 Onchocerca gutturos  TATTAATTTTATAGTTACTACTCAGAATATGCGATCTACTGCTGTAACTTTGGATCAGAT
KP760201.1 Onchocerca gutturos  TATTAATTTTATAGTTACTACTCAGAATATGCGATCTACTGCTGTAACTTTGGATCAGAT
KX853325.1 Onchocerca lienalis  TATTAATTTTATGGTTACTACTCAGAATATGCGGTCTACTGCTGTGACTTTGGATCAAAT
KT001213.1 Onchocerca jakutens  TATTAATTTTATGGTTACTACTCAAAATATACGATCTACTGCTGTAACTTTGGATCAAAT
JQ316672.1 Onchocerca fasciata  TATTAATTTTATGGTTACTACTCAGAATATGCGATCTACTGCTGTAACTTTGGATCAGAT
MG188678.1 Onchocerca fasciata  TATTAATTTTATGGTTACTACTCAGAATATGCGATCTACTGCTGTAACTTTGGACCAGAT
MT002817.1 Onchocerca fasciata  TATTAATTTTATGGTTACTACTCAGAATATGCGATCTACTGCTGTAACTTTGGATCAGAT
KC167355.1 Onchocerca volvulus  TATTAATTTTATGGTAACTACTCAGAATATACGGTCTACTGCTGTGACTTTGGATCAAAT
MH190075.1 Onchocerca volvulus  TATTAATTTTATGGTAACTACTCAGAATATACGGTCTACTGCTGTGACTTTGGATCAAAT
KC167354.1 Onchocerca sp. 'Sii  TATTAATTTTATGGTAACTACTCAGAATATACGGTCCACTGCTGTGACTTTGGATCAAAT
KC167352.1 Onchocerca sp. 'Sii  TATTAATTTTATGGTAACTACTCAGAATATACGGTCCACTGCTGTGACTTTGGATCAAAT
KC167353.1 Onchocerca sp. 'Sii  TATTAATTTTATGGTAACTACTCAGAATATACGGTCCACTGCTGTGACTTTGGATCAAAT
AJ271618.1 Onchocerca ochengi   TATTAATTTTATGGTAACTACTCAGAATATACGGTCTACTGCTGTGACTTTGGATCAAAT
```

```
KC167358.1 Onchocerca ochengi   TATTAATTTTATGGTAACTACTCAGAATATACGGTCTACTGCTGTGACTTTGGATCAAAT
KC167351.1 Onchocerca ochengi   TATTAATTTTATGGTAACTACTCAGAATATACGGTCTACTGCTGTGACTTTGGATCAAAT
NC_031891.2 Onchocerca ochengi  TATTAATTTTATGGTAACTACTCAGAATATACGGTCTACTGCTGTGACTTTGGATCAAAT
KC167350.1 Onchocerca ochengi   TATTAATTTTATGGTAACTACTCAGAATATACGGTCTACTGCTGTGACTTTGGATCAAAT
KX181289.2 Onchocerca ochengi   TATTAATTTTATGGTAACTACTCAGAATATACGGTCTACTGCTGTGACTTTGGATCAAAT

                                         310       320       330       340       350       360
                                ....|....|....|....|....|....|....|....|....|....|....|....|

MN564742.1 Dirofilaria sp. 'ho  TAGTATGTTTGTTTGAACTTCTTATTTGACTTCTTTTCTTTTGGTTTTATCTGTTCCTGT
MF695085.1 Dirofilaria repens   TAGTATGTTTGTTTGAACTTCTTATTTGACTTCTTTTCTTTTAGTTTTATCTGTTCCTGT
AB518693.1 Onchocerca sp. wild  TAGTATGTTTGTTTGAACTTCTTATTTAACTTCTTTTTTGTTAGTATTGTCTGTTCCTGT
AB518694.1 Onchocerca sp. wild  TAGTATGTTTGTTTGAACTTCTTATTTAACTTCTTTTTTGTTAGTCTTGTCTGTTCCTGT
AB518874.1 Onchocerca dewittei  TAGTATATTTGTTTGAACTTCTTATTTAACTTCTTTTTTGTTGGTATTGTCTGTTCCTGT
AB518690.1 Onchocerca dewittei  TAGTATGTTTGTTTGAACTTCTTATTTAACTTCTTTTTTGTTGGTATTGTCTGTTCCTGT
AB518692.1 Onchocerca dewittei  TAGTATGTTTGTTTGAACTTCTTATTTAACTTCTTTTTTGTTGGTATTGTCTGTTCCTGT
AB518689.1 Onchocerca dewittei  TAGTATGTTTGTTTGAACTTCTTATTTAACTTCTTTTTTGTTGGTATTGTCTGTTCCTGT
AM749268.1 Onchocerca eberhard  TAGTATGTTTGTTTGAACTTCTTATTTGACTTCTTTTTTATTGGTATTGTCTGTGCCTGT
LC318284.1 Onchocerca flexuosa  TAGTATGTTTGTTTGGACTTCTTATTTAACTTCTTTTTTGTTGGTTTTATCTGTTCCTGT
AM749269.1 Onchocerca skrjabin  TAGTATGTTTGTTTGGACTTCTTATTTGACTTCTTTTTTGTTGGTATTGTCTGTACCTGT
KX898458.1 Onchocerca boehmi c  TAGTATATTTGTTTGGACTTCTTATTTAACTTCTTTTTTGCTGGTGTTATCTGTGCCTAT
MG029461.1 Onchocerca sp. ALDA  TAGTATATTTGTTTGAACTTCTTATTTGACTTCTTTTTTGTTAGTTTTATCTGTGCCTGT
MG029460.1 Onchocerca sp. ALDA  TAGTATATTTGTTTGAACTTCTTATTTGACTTCTTTTTTGTTAGTTTTATCTGTGCCTGT
MG677940.1 Onchocerca lupi cyt  TAGTATGTTTGTCTGGACTTCTTATTTAACTTCTTTTTTATTAGTGTTATCTGTTCCTGT
EF521410.1 Onchocerca lupi fro  TAGTATGTTTGTCTGGACTTCTTATTTAACTTCTTTTTTATTAGTGTTATCTGTTCCTGT
HQ207644.1 Onchocerca lupi fro  TAGTATGTTTGTCTGGACTTCTTATTTAACTTCTTTTTTATTAGTATTATCTGTTCCTGT
KC686702.1 Onchocerca lupi cyt  TAGTATGTTTGTCTGGACTTCTTATTTAACTTCTTTTTTATTAGTATTATCTGTTCCTGT
AJ415417.1 Onchocerca lupi mit  TAGTATATTTGTCTGGACTTCTTATTTAACTTCTTTTTTATTAGTATTATCTGTTCCTGT
KP283476.1 Onchocerca lupi iso  TAGTATGTTTGTCTGGACTTCTTATTTAACTTCTTTTTTATTAGTATTATCTGTTCCTGT
KP283477.1 Onchocerca lupi iso  TAGTATGTTTGTCTGGACTTCTTATTTAACTTCTTTTTTATTAGTATTATCTGTTCCTGT
JX080030.1 Onchocerca lupi iso  TAGTATGTTTGTCTGGACTTCTTATTTAACTTCTTTTTTATTAGTATTATCTGTTCCTGT
JX183106.1 Onchocerca lupi cyt  TAGTATGTTTGTCTGGACTTCTTATTTAACTTCTTTTTTATTAGTATTATCTGTTCCTGT
KX853328.1 Onchocerca lupi vou  TAGTATGTTTGTCTGGACTTCTTATTTAACTTCTTTTTTATTAGTGTTATCTGTTCCTGT
JX080029.1 Onchocerca lupi iso  TAGTATGTTTGTCTGGACTTCTTATTTAACTTCTTTTTTATTAGTATTATCTGTTCCTGT
EF521409.1 Onchocerca lupi fro  TAGTATGTTTGTCTGGACTTCTTATTTAACTTCTTTTTTATTAGTATTATCTGTTCCTGT
AJ271616.1 Onchocerca gibsoni   TAGTATATTTGTTTGAACTTCTTATTTGACTTCTTTTTTGTTAGTTTTGTCTGTGCCTGT
AJ271617.1 Onchocerca gutturos  TAGTATGTTTGTTTGGACTTCTTATTTGACTTCTTTTTTGTTGGTTTTATCTGTGCCTGT
KP760201.1 Onchocerca gutturos  TAGTATGTTTGTTTGAACTTCTTATTTGACTTCTTTTTTGTTGGTTTTATCTGTGCCTGT
KX853325.1 Onchocerca lienalis  TAGTATATTTGTTTGAACTTCTTATTTGACTTCTTTTTTGTTGGTTTTGTCTGTACCTGT
KT001213.1 Onchocerca jakutens  TAGTATATTTGTTTGAACTTCTTATTTAACTTCTTTTTTGTTGGTTTTATCTGTGCCTGT
JQ316672.1 Onchocerca fasciata  TAGTATGTTTGTTTGGACTTCTTATTTGACTTCTTTTTTGTTGGTTTTGTCTGTGCCTGT
MG188678.1 Onchocerca fasciata  TAGTATGTTTGTTTGGACTTCTTATTTGACTTCTTTTTTGTTGGTTTTGTCTGTGCCTGT
MT002817.1 Onchocerca fasciata  TAGTATGTTTGTTTGGACTTCTTATTTGACTTCTTTTTTGTTGGTTTTGTCTGTGCCTGT
KC167355.1 Onchocerca volvulus  TAGTATGTTTGTTTGGACTTCTTATTTGACTTCTTTTTTATTAGTTTTATCTGTGCCTGT
MH190075.1 Onchocerca volvulus  TAGTATGTTTGTTTGGACTTCTTATTTGACTTCTTTTTTATTAGTTTTATCTGTGCCTGT
KC167354.1 Onchocerca sp. 'Sii  TAGTATGTTTGTTTGGACTTCTTATTTGACTTCTTTTTTATTAGTTTTGTCTGTGCCTGT
KC167352.1 Onchocerca sp. 'Sii  TAGTATGTTTGTTTGGACTTCTTATTTGACTTCTTTTTTATTAGTTTTGTCTGTGCCTGT
KC167353.1 Onchocerca sp. 'Sii  TAGTATGTTTGTTTGGACTTCTTATTTGACTTCTTTTTTATTAGTTTTGTCTGTGCCTGT
AJ271618.1 Onchocerca ochengi   TAGTATGTTTGTTTGGACTTCTTATTTGACTTCTTTTTTGTTAGTTTTGTCTGTGCCTGT
KC167358.1 Onchocerca ochengi   TAGTATGTTTGTTTGGACTTCTTATTTGACTTCTTTTTTGTTAGTTTTGTCTGTGCCTGT
KC167351.1 Onchocerca ochengi   TAGTATGTTTGTTTGGACTTCTTATTTGACTTCTTTTTTGTTAGTTTTGTCTGTGCCTGT
NC_031891.2 Onchocerca ochengi  TAGTATGTTTGTTTGGACTTCTTATTTGACTTCTTTTTTGTTAGTTTTGTCTGTGCCTGT
KC167350.1 Onchocerca ochengi   TAGTATGTTTGTTTGGACTTCTTATTTGACTTCTTTTTTGTTAGTTTTGTCTGTGCCTGT
KX181289.2 Onchocerca ochengi   TAGTATGTTTGTTTGGACTTCTTATTTGACTTCTTTTTTGTTAGTTTTGTCTGTGCCTGT

                                         370       380       390       400       410       420
                                ....|....|....|....|....|....|....|....|....|....|....|....|

MN564742.1 Dirofilaria sp. 'ho  TTTGGCTGGTTCTTTGTTGTTTTTATTGTTGGATCGTAATTTTAATACTTCTTTTTATGA
MF695085.1 Dirofilaria repens   TTTAGCTGGTTCTTTGTTGTTTTTGTTATTAGATCGTAATTTTAATACTTCTTTTTATGA
AB518693.1 Onchocerca sp. wild  TTTGGCTGGTTCTTTATTGTTTTTATTGTTAGATCGTAATTTTAATACTTCTTTTTATGA
AB518694.1 Onchocerca sp. wild  TTTGGCTGGTTCTTTATTGTTTTTATTGTTAGATCGTAATTTTAATACTTCTTTTTATGA
AB518874.1 Onchocerca dewittei  TTTGGCTGGGTCTTTATTGTTTTTATTGTTGGATCGTAATTTTAATACTTCTTTTTATGA
AB518690.1 Onchocerca dewittei  TTTGGCTGGGTCTTTATTGTTTTTATTGTTGGATCGTAATTTTAATACTTCTTTTTATGA
AB518692.1 Onchocerca dewittei  TTTGGCTGGGTCTTTATTGTTTTTATTGTTGGATCGTAATTTTAATACTTCTTTTTATGA
AB518689.1 Onchocerca dewittei  TTTGGCTGGGTCTTTATTGTTTTTATTGTTGGATCGTAATTTTAATACTTCTTTTTATGA
AM749268.1 Onchocerca eberhard  TTTGGCTGGATCTTTATTGTTTTTGTTGTTGGATCGTAATTTTAATACTTCTTTTTATGA
LC318284.1 Onchocerca flexuosa  TTTAGCAGGATCATTGTTGTTTTTATTGTTGGATCGTAATTTTAATACTTCTTTTTACGA
AM749269.1 Onchocerca skrjabin  TTTAGCTGGTTCTTTGTTGTTTTTGTTGTTGGATCGTAATTTTAATACTTCTTTTTATGA
KX898458.1 Onchocerca boehmi c  TTTGGCTGGGTCTTTGTTATTTTTGTTGTTGGATCGTAATTTTAATACTTCTTTTTATGA
MG029461.1 Onchocerca sp. ALDA  TTTGGCTGGGTCTTTATTATTTTTGTTACTTGATCGTAATTTTAATACTTCTTTTTATGA
MG029460.1 Onchocerca sp. ALDA  TTTGGCTGGGTCTTTATTATTTTTGTTACTTGATCGTAATTTTAATACTTCTTTTTATGA
MG677940.1 Onchocerca lupi cyt  TTTAGCTGGGTCTTTGTTATTTTTGTTGTTGGATCGTAATTTTAGTACTTCTTTTTATGA
EF521410.1 Onchocerca lupi fro  TTTAGCTGGGTCTTTGTTATTTTTGTTGTTGGATCGTAATTTTAGTACTTCTTTTTATGA
HQ207644.1 Onchocerca lupi fro  TTTAGCTGGGTCTTTGTTATTTTTGTTGTTGGATCGTAATTTTAGTACTTCTTTTTATGA
KC686702.1 Onchocerca lupi cyt  TTTAGCTGGGTCTTTGTTATTTTTGTTGTTGGATCGTAATTTTAGTACTTCTTTTTATGA
AJ415417.1 Onchocerca lupi mit  TTTAGCTGGGTCTTTGTTATTTTTGTTGTTGGATCGTAATTTTAGTACTTCTTTTTATGA
KP283476.1 Onchocerca lupi iso  TTTAGCTGGGTCTTTGTTATTTTTGTTGTTGGATCGTAATTTTAGTACTTCTTTTTATGA
KP283477.1 Onchocerca lupi iso  TTTAGCTGGGTCTTTGTTATTTTTGTTGTTGGATCGTAATTTTAGTACTTCTTTTTATGA
JX080030.1 Onchocerca lupi iso  TTTAGCTGGGTCTTTGTTATTTTTGTTGTTGGATCGTAATTTTAGTACTTCTTTTTATGA
```

```
JX183106.1 Onchocerca lupi cyt    TTTAGCTGGGTCTTTGTTATTTTTGTTGTTGGATCGTAATTTTAGTACTTCTTTTTATGA
KX853328.1 Onchocerca lupi vou    TTTAGCTGGGTCTTTGTTATTTTTGTTGTTGGATCGTAATTTTAGTACTTCTTTTTATGA
JX080029.1 Onchocerca lupi iso    TTTAGCTGGGTCTTTGTTATTTTTGTTGTTGGATCGTAATTTTAGTACTTCTTTTTATGA
EF521409.1 Onchocerca lupi fro    TTTAGCTGGGTCTTTGTTATTTTTGTTGTTGGATCGTAATTTTAGTACTTCTTTTTATGA
AJ271616.1 Onchocerca gibsoni     TTTAGCTGGTTCTTTGTTGTTTTTGTTGTTGGATCGTAATTTTAATACTTCTTTTTATGA
AJ271617.1 Onchocerca gutturos    TTTAGCTGGTTCTTTGTTATTTTTGTTGTTGGATCGTAATTTTAATACTTCTTTTTATGA
KP760201.1 Onchocerca gutturos    TTTAGCTGGTTCTTTGTTGTTTTTGTTGTTGGATCGTAATTTTAATACTTCTTTTTATGA
KX853325.1 Onchocerca lienalis    TTTAGCAGGTTCTTTGTTGTTTTTATTGTTGGATCGTAATTTTAATACTTCTTTTTATGA
KT001213.1 Onchocerca jakutens    TTTGGCTGGTTCTTTGTTGTTTTTATTGTTGGATCGTAATTTTAATACTTCTTTTTATGA
JQ316672.1 Onchocerca fasciata    TTTGGCGGGTTCTTTGTTATTTTTGTTGTTAGATCGTAATTTTAATACTTCTTTTTATGA
MG188678.1 Onchocerca fasciata    TTTGGCGGGTTCTTTGTTATTTTTGTTGTTAGATCGTAATTTTAATACTTCTTTTTATGA
MT002817.1 Onchocerca fasciata    TTTGGCGGGTTCTTTGTTATTTTTGTTGTTAGATCGTAATTTTAATACTTCTTTTTATGA
KC167355.1 Onchocerca volvulus    TTTGGCTGGTTCTTTATTGTTTTTGTTGTTGGATCGTAATTTTAATACTTCTTTTTATGA
MH190075.1 Onchocerca volvulus    TTTGGCTGGTTCTTTATTGTTTTTGTTGTTGGATCGTAATTTTAATACTTCTTTTTATGA
KC167354.1 Onchocerca sp. 'Sii    TTTGGCTGGTTCTTTATTGTTTTTGTTGTTGGATCGTAATTTTAATACTTCTTTTTATGA
KC167352.1 Onchocerca sp. 'Sii    TTTGGCTGGTTCTTTATTGTTTTTGTTGTTGGATCGTAATTTTAATACTTCTTTTTATGA
KC167353.1 Onchocerca sp. 'Sii    TTTGGCTGGTTCTTTATTGTTTTTGTTGTTGGATCGTAATTTTAATACTTCTTTTTATGA
AJ271618.1 Onchocerca ochengi     TTTGGCTGGTTCTTTATTGTTTTTGTTGTTGGATCGTAATTTTAATACTTCTTTTTATGA
KC167358.1 Onchocerca ochengi     TTTGGCTGGTTCTTTATTGTTTTTGTTGTTGGATCGTAATTTTAATACTTCTTTTTATGA
KC167351.1 Onchocerca ochengi     TTTGGCTGGTTCTTTATTGTTTTTGTTGTTGGATCGTAATTTTAATACTTCTTTTTATGA
NC_031891.2 Onchocerca ochengi    TTTGGCTGGTTCTTTATTGTTTTTGTTGTTGGATCGTAATTTTAATACTTCTTTTTATGA
KC167350.1 Onchocerca ochengi     TTTGGCTGGTTCTTTATTGTTTTTGTTGTTGGATCGTAATTTTAATACTTCTTTTTATGA
KX181289.2 Onchocerca ochengi     TTTGGCTGGTTCTTTATTGTTTTTGTTGTTGGATCGTAATTTTAATACTTCTTTTTATGA

                                           430       440       450       460       470       480
                                  ....|....|....|....|....|....|....|....|....|....|....|....|
MN564742.1 Dirofilaria sp. 'ho    TACTAAAAAGGGGGGTAATCCTTTATTGTATCAACATTTGTTTTGATTTTTTGGTCATCC
MF695085.1 Dirofilaria repens     TACTAAAAAGGGGGGTAATCCTTTGTTGTATCAGCATTTGTTTTGATTTTTTGGTCATCC
AB518693.1 Onchocerca sp. wild    TACAAAAAAGGGAGGTAATCCATTATTGTATCAGCATTTATTTTGATTTTTTGGTCATCC
AB518694.1 Onchocerca sp. wild    TACAAAAAAGGGAGGTAATCCATTATTGTATCAGCATTTATTTTGATTTTTTGGTCATCC
AB518874.1 Onchocerca dewittei    TACTAAAAAAGGGGGCAATCCTTTGTTGTATCAGCATTTGTTTTGATTTTTTGGCCATCC
AB518690.1 Onchocerca dewittei    TACTAAAAAAGGGGGCAATCCTTTGTTGTATCAGCATTTGTTTTGATTTTTTGGCCATCC
AB518692.1 Onchocerca dewittei    TACTAAAAAAGGGGGCAATCCTTTGTTGTATCAGCATTTGTTTTGATTTTTTGGCCATCC
AB518689.1 Onchocerca dewittei    TACTAAAAAAGGGGGCAATCCTTTGTTGTATCAGCATTTGTTTTGATTTTTTGGCCATCC
AM749268.1 Onchocerca eberhard    TACTAAGAAGGGGGGTAATCCTTTATTGTATCAGCATTTATTTTGATTTTTTGGTCATCC
LC318284.1 Onchocerca flexuosa    TACTAAAAAGGGTGGTAATCCTCTTTTATATCAGCATTTATTTTGATTTTTTGGTCATCC
AM749269.1 Onchocerca skrjabin    TACTAAAAAGGGGGGTAATCCTTTATTGTATCAGCATTTGTTTTGATTTTTTGGTCATCC
KX898458.1 Onchocerca boehmi c    TACTAAAAAAGGTGGGAATCCGTTATTGTATCAGCATTTGTTTTGATTTTTTGGTCATCC
MG029461.1 Onchocerca sp. ALDA    TACTAAGAAGGGTGGTAATCCATTATTGTATCAACATTTATTTTGATTTTTTGGTCACCC
MG029460.1 Onchocerca sp. ALDA    TACTAAGAAGGGTGGTAATCCATTATTGTATCAACATTTATTTTGATTTTTTGGTCACCC
MG677940.1 Onchocerca lupi cyt    TACTAAAAAAGGTGGTAATCCTTTGTTGTATCAGCATTTGTTTTGGTTTTTTGGTCATCC
EF521410.1 Onchocerca lupi fro    TACTAAAAAAGGTGGTAATCCTTTGTTGTATCAGCATTTGTTTTGGTTTTTTGGTCATCC
HQ207644.1 Onchocerca lupi fro    TACTAAAAAAGGTGGTAATCCTTTGTTGTATCAGCACTTGTTTTGGTTTTTCGGTCATCC
KC686702.1 Onchocerca lupi cyt    TACTAAAAAAGGTGGTAATCCTTTGTTGTATCAGCACTTGTTTTGGTTTTTCGGTCATCC
AJ415417.1 Onchocerca lupi mit    TACTAAAAAAGGTGGTAATCCTTTGTTGTATCAGCACTTGTTTTGGTTTTTCGGTCATCC
KP283476.1 Onchocerca lupi iso    TACTAAAAAAGGTGGTAATCCTTTGTTGTATCAGCACTTGTTTTGGTTTTTCGGTCATCC
KP283477.1 Onchocerca lupi iso    TACTAAAAAAGGTGGTAATCCTTTGTTGTATCAGCACTTGTTTTGGTTTTTCGGTCATCC
JX080030.1 Onchocerca lupi iso    TACTAAAAAAGGTGGTAATCCTTTGTTGTATCAGCACTTGTTTTGGTTTTTCGGTCATCC
JX183106.1 Onchocerca lupi cyt    TACTAAAAAAGGTGGTAATCCTTTGTTGTATCAGCACTTGTTTTGGTTTTTCGGTCATCC
KX853328.1 Onchocerca lupi vou    TACTAAAAAAGGTGGTAATCCTTTGTTGTATCAGCATTTGTTTTGGTTTTTTGGTCATCC
JX080029.1 Onchocerca lupi iso    TACTAAAAAAGGTGGTAATCCTTTGTTGTATCAGCACTTGTTTTGGTTTTTCGGTCATCC
EF521409.1 Onchocerca lupi fro    TACTAAAAAAGGTGGTAATCCTTTGTTGTATCAGCACTTGTTTTGGTTTTTCGGTCATCC
AJ271616.1 Onchocerca gibsoni     TACTAAAAAGGGAGGTAATCCTTTATTGTATCAGCATTTGTTTTGATTTTTTGGTCATCC
AJ271617.1 Onchocerca gutturos    TACTAAGAAGGGGGGTAACCCTTTGTTGTATCAGCATTTGTTTTGATTTTTTGGTCATCC
KP760201.1 Onchocerca gutturos    TACTAAGAAGGGGGGTAACCCTTTGTTGTATCAGCATTTGTTCTGATTTTTTGGTCATCC
KX853325.1 Onchocerca lienalis    TACTAAGAAGGGGGGTAATCCTTTGTTATATCAGCATTTGTTTTGATTTTTTGGTCATCC
KT001213.1 Onchocerca jakutens    CACTAAAAAGGGTGGTAATCCTTTGTTATATCAGCATTTATTTTGATTTTTTGGTCACCC
JQ316672.1 Onchocerca fasciata    CACTAAGAAAGGGGGTAATCCTTTGCTGTATCAGCATTTATTTTGATTTTTTGGTCATCC
MG188678.1 Onchocerca fasciata    CACTAAGAAAGGGGGTAATCCTTTGCTGTATCAGCATTTATTTTGATTTTTTGGTCATCC
MT002817.1 Onchocerca fasciata    CACTAAGAAAGGGGGTAATCCTTTGCTGTATCAGCATTTATTTTGATTTTTTGGTCATCC
KC167355.1 Onchocerca volvulus    TACTAAGAAGGGGGGTAATCCTTTGTTGTATCAGCATTTGTTTTGATTTTTTGGTCATCC
MH190075.1 Onchocerca volvulus    TACTAAGAAGGGGGGTAATCCTTTGTTGTATCAGCATTTGTTTTGATTTTTTGGTCATCC
KC167354.1 Onchocerca sp. 'Sii    TACTAAGAAGGGGGGTAATCCTTTGTTGTATCAGCATTTGTTTTGATTTTTTGGTCATCC
KC167352.1 Onchocerca sp. 'Sii    TACTAAGAAGGGGGGTAATCCTTTGTTGTATCAGCATTTGTTTTGATTTTTTGGTCATCC
KC167353.1 Onchocerca sp. 'Sii    TACTAAGAAGGGGGGTAATCCTTTGTTGTATCAGCATTTGTTTTGATTTTTTGGTCATCC
AJ271618.1 Onchocerca ochengi     TACTAAGAAGGGGGGTAATCCTTTGTTGTATCAGCATTTGTTTTGATTTTTTGGTCATCC
KC167358.1 Onchocerca ochengi     TACTAAGAAGGGGGGTAATCCTTTGTTGTATCAGCATTTGTTTTGATTTTTTGGTCATCC
KC167351.1 Onchocerca ochengi     TACTAAGAAGGGGGGTAATCCTTTGTTGTATCAGCATTTGTTTTGATTTTTTGGTCATCC
NC_031891.2 Onchocerca ochengi    TACTAAGAAGGGGGGTAATCCTTTGTTGTATCAGCATTTGTTTTGATTTTTTGGTCATCC
KC167350.1 Onchocerca ochengi     TACTAAGAAGGGGGGTAATCCTTTGTTGTATCAGCATTTGTTTTGATTTTTTGGTCATCC
KX181289.2 Onchocerca ochengi     TACTAAGAAGGGGGGTAATCCTTTGTTGTATCAGCATTTGTTTTGATTTTTTGGTCATCC

                                           490       500       510       520       530       540
                                  ....|....|....|....|....|....|....|....|....|....|....|....|
MN564742.1 Dirofilaria sp. 'ho    TGAGGTTTATGTTATTATTTTACCTGTTTTTGGTATTATTAGTGAATGTGTTTTATTTTT
MF695085.1 Dirofilaria repens     TGAGGTTTATGTTATTATTTTGCCTGTTTTTGGTATTATTAGTGAATGTGTTTTGTTTTT
AB518693.1 Onchocerca sp. wild    TGAGGTTTATGTTATTATTTTGCCGGTTTTTGGTATTATTAGGGAAGCGGTGTTATTTTT
AB518694.1 Onchocerca sp. wild    TGAGGTTTATGTTATTATTTTGCCGGTTTTTGGTATTATTAGGGAAGCGGTGTTATTTTT
```

```
AB518874.1 Onchocerca dewittei   TGAGGTTTATGTTATTATTTTACCTGTTTTTGGTATTATCAGGGAAGCTGTATTATTTTT
AB518690.1 Onchocerca dewittei   TGAGGTTTATGTTATTATTTTACCTGTTTTTGGTATTATCAGGGAAGCTGTATTATTTTT
AB518692.1 Onchocerca dewittei   TGAGGTTTATGTTATTATTTTACCTGTTTTTGGTATTATCAGGGAAGCTGTATTATTTTT
AB518689.1 Onchocerca dewittei   TGAGGTTTATGTTATTATTTTACCTGTTTTTGGTATTATCAGGGAAGCTGTATTATTTTT
AM749268.1 Onchocerca eberhard   AGAGGTTTATGTTATTATTTTACCTGTTTTTGGTATTATTAGGGAGGCTGTGTTATTTTT
LC318284.1 Onchocerca flexuosa   TGAAGTTTATGTTATTATTTTGCCGGTTTTTGGTATTATTAGAGAGGCTGTGCTGTTTTT
AM749269.1 Onchocerca skrjabin   TGAGGTTTATGTTATTATTTTGCCAGTTTTTGGTATTATTAGAGAGGCAGTGTTGTTTTT
KX898458.1 Onchocerca boehmi c   TGAAGTTTATGTTATTATTTTACCTGTGTTCGGTATTATTAGTGAGGCTGTTTTATTTTT
MG029461.1 Onchocerca sp. ALDA   TGAGGTTTATGTTATTATTTTGCCAGTTTTTGGTATTATTAGGGAGGCGGTTTTATTTTT
MG029460.1 Onchocerca sp. ALDA   TGAGGTTTATGTTATTATTTTGCCAGTTTTTGGTATTATTAGGGAGGCGGTTTTATTTTT
MG677940.1 Onchocerca lupi cyt   TGAGGTTTATGTTATTATTTTACCGGTTTTTGGTATTATTAGGGAGGCAGTTTTATTTTT
EF521410.1 Onchocerca lupi fro   TGAGGTTTATGTTATTATTTTACCGGTTTTTGGTATTATTAGGGAGGCAGTTTTATTTTT
HQ207644.1 Onchocerca lupi fro   CGAGGTTTATGTTATTATTTTACCGGTTTTTGGTATTATTAGGGAGGCAGTTTTATTTTT
KC686702.1 Onchocerca lupi cyt   CGAGGTTTATGTTATTATTTTACCGGTTTTTGGTATTATTAGGGAGGCAGTTTTATTTTT
AJ415417.1 Onchocerca lupi mit   CGAGGTTTATGTTATTATTTTACCGGTTTTTGGTATTATTAGGGAGGCAGTTTTATTTTT
KP283476.1 Onchocerca lupi iso   CGAGGTTTATGTTATTATTTTACCGGTTTTTGGTATTATTAGGGAGGCAGTTTTATTTTT
KP283477.1 Onchocerca lupi iso   CGAGGTTTATGTTATTATTTTACCGGTTTTTGGTATTATTAGGGAGGCAGTTTTATTTTT
JX080030.1 Onchocerca lupi iso   CGAGGTTTATGTTATTATTTTACCGGTTTTTGGTATTATTAGGGAGGCAGTTTTATTTTT
JX183106.1 Onchocerca lupi cyt   CGAGGTTTATGTTATTATTTTACCGGTTTTTGGTATTATTAGGGAGGCAGTTTTATTTTT
KX853328.1 Onchocerca lupi vou   TGAGGTTTATGTTATTATTTTACCGGTTTTTGGTATTATTAGGGAGGCAGTTTTATTTTT
JX080029.1 Onchocerca lupi iso   CGAGGTTTATGTTATTATTTTACCGGTTTTTGGTATTATTAGGGAGGCAGTTTTATTTTT
EF521409.1 Onchocerca lupi fro   CGAGGTTTATGTTATTATTTTACCGGTTTTTGGTATTATTAGGGAGGCAGTTTTATTTTT
AJ271616.1 Onchocerca gibsoni    TGAGGTTTATGTTATTATTTTGCCAGTTTTTGGTATTATTAGAGAAGCTGTTTTATTTTT
AJ271617.1 Onchocerca gutturos   TGAGGTTTATGTTATTATTTTGCCTGTTTTTGGTATTATTAGAGAGGCGGTTTTGTTTTT
KP760201.1 Onchocerca gutturos   TGAGGTTTATGTTATTATTTTGCCTGTTTTTGGTATTATTAGAGAGGCGGTTTTGTTTTT
KX853325.1 Onchocerca lienalis   TGAGGTTTATGTTATTATTTTGCCGGTTTTTGGTATTATTAGGGAAGCTGTTTTGTTTTT
KT001213.1 Onchocerca jakutens   TGAGGTTTATGTTATTATTTTACCGGTTTTTGGTATTATTAGGGAGGCGGTTTTGTTTTT
JQ316672.1 Onchocerca fasciata   TGAGGTTTATGTTATTATTTTGCCGGTTTTTGGTATTATTAGAGAGGCGGTTTTATTTTT
MG188678.1 Onchocerca fasciata   TGAGGTTTATGTTATTATTTTGCCGGTTTTTGGTATTATTAGAGAGGCGGTTTTATTTTT
MT002817.1 Onchocerca fasciata   TGAGGTTTATGTTATTATTTTGCCGGTTTTTGGTATTATTAGAGAGGCGGTTTTATTTTT
KC167355.1 Onchocerca volvulus   TGAGGTGTATGTTATTATTTTACCTGTTTTTGGTATTATTAGGGAGGCGGTTTTATTTTT
MH190075.1 Onchocerca volvulus   TGAGGTGTATGTTATTATTTTACCTGTTTTTGGTATTATTAGGGAGGCGGTTTTATTTTT
KC167354.1 Onchocerca sp. 'Sii   TGAGGTGTATGTTATTATTTTACCTGTTTTCGGTATTATTAGGGAAGCGGTTTTATTTTT
KC167352.1 Onchocerca sp. 'Sii   TGAGGTGTATGTTATTATTTTACCTGTTTTTGGTATTATTAGGGAAGCGGTTTTATTTTT
KC167353.1 Onchocerca sp. 'Sii   TGAGGTGTATGTTATTATTTTACCTGTTTTTGGTATTATTAGGGAAGCGGTTTTATTTTT
AJ271618.1 Onchocerca ochengi    TGAGGTGTATGTTATTATTTTACCTGTTTTTGGTATTATTAGGGAAGCGGTTTTATTTTT
KC167358.1 Onchocerca ochengi    TGAGGTGTATGTTATTATTTTACCTGTTTTTGGTATTATTAGGGAAGCGGTTTTATTTTT
KC167351.1 Onchocerca ochengi    TGAGGTGTATGTTATTATTTTACCTGTTTTTGGTATTATTAGGGAAGCGGTTTTATTTTT
NC_031891.2 Onchocerca ochengi   TGAGGTGTATGTTATTATTTTACCTGTTTTTGGTATTATTAGGGAAGCGGTTTTATTTTT
KC167350.1 Onchocerca ochengi    TGAGGTGTATGTTATTATTTTACCTGTTTTTGGTATTATTAGGGAAGCGGTTTTATTTTT
KX181289.2 Onchocerca ochengi    TGAGGTGTATGTTATTATTTTACCTGTTTTTGGTATTATTAGGGAAGCGGTTTTATTTTT

                                           550       560       570       580       590       600
                                 ....|....|....|....|....|....|....|....|....|....|....|....|

MN564742.1 Dirofilaria sp. 'ho   GACTGATAAGGATCGTTTGTTTGGTCAGACTAGAATAACTTTTGCTTCTATTTGGATTGC
MF695085.1 Dirofilaria repens    GACTGATAAGGATCGTTTGTTTGGTCAGACTAGTATAACTTTTGCTTCTATTTGGATTGC
AB518693.1 Onchocerca sp. wild   AACTGATAAGGATCGTTTGTTTGGTCAAACTAGAATAACTTTTGCTTCTATTTGAATTGC
AB518694.1 Onchocerca sp. wild   AACTGATAAGGATCGTTTGTTTGGTCAAACTAGAATAACTTTTGCTTCTATTTGAATTGC
AB518874.1 Onchocerca dewittei   GACTGATAAAGATCGTCTATTTGGTCAAACTAGAATAACTTTTGCATCTATTTGAATTGC
AB518690.1 Onchocerca dewittei   GACTGATAAAGATCGTTTATTTGGTCAAACTAGAATAACTTTTGCATCTATTTGGATTGC
AB518692.1 Onchocerca dewittei   GACTGATAAAGATCGTTTATTTGGTCAAACTAGAATAACTTTTGCATCTATTTGAATTGC
AB518689.1 Onchocerca dewittei   GACTGATAAAGATCGTTTATTTGGTCAAACTAGAATAACTTTTGCATCTATTTGGATTGC
AM749268.1 Onchocerca eberhard   GACTGATAAGGATCGTTTGTTTGGCCAGACTAGGATAACTTTTGCTTCTATTTGAATTGC
LC318284.1 Onchocerca flexuosa   AACTGATAAGGATCGTTTATTTGGTCAGACTAGAATGACTTTTGCTTCTATTTGAATTGC
AM749269.1 Onchocerca skrjabin   AACTGATAAGGACCGTTTGTTTGGTCAGACTAGGATAACTTTTGCTTCTATTTGAATTGC
KX898458.1 Onchocerca boehmi c   AACTGATAAGGATCGTTTGTTTGGTCAGACTAGTATAACTTTTGCTTCTATTTGGATCGC
MG029461.1 Onchocerca sp. ALDA   GACTGATAAGGATCGTTTATTTGGTCAAACTAGAATGACTTTTGCTTCTATTTGAATTGC
MG029460.1 Onchocerca sp. ALDA   GACTGATAAGGATCGTTTATTTGGTCAAACTAGAATGACTTTTGCTTCTATTTGAATTGC
MG677940.1 Onchocerca lupi cyt   AACTGATAAGGACCGTTTGTTTGGTCAAACTAGGATGACTTTTGCTTCTATTTGGATTGC
EF521410.1 Onchocerca lupi fro   AACTGATAAGGACCGTTTGTTTGGTCAAACTAGGATGACTTTTGCTTCTATTTGGATTGC
HQ207644.1 Onchocerca lupi fro   GACTGATAAGGATCGTTTGTTTGGTCAAACTAGGATGACTTTTGCTTCTATTTGGATTGC
KC686702.1 Onchocerca lupi cyt   GACTGATAAGGATCGTTTGTTTGGTCAAACTAGGATGACTTTTGCTTCTATTTGGATTGC
AJ415417.1 Onchocerca lupi mit   GACTGATAAGGATCGTTTGTTTGGTCAAACTAGGATGACTTTTGCTTCTATTTGGATTGC
KP283476.1 Onchocerca lupi iso   GACTGATAAGGATCGTTTGTTTGGTCAAACTAGGATGACTTTTGCTTCTATTTGGATTGC
KP283477.1 Onchocerca lupi iso   GACTGATAAGGATCGTTTGTTTGGTCAAACTAGGATGACTTTTGCTTCTATTTGGATTGC
JX080030.1 Onchocerca lupi iso   GACTGATAAGGATCGTTTGTTTGGTCAAACTAGGATGACTTTTGCTTCTATTTGGATTGC
JX183106.1 Onchocerca lupi cyt   GACTGATAAGGATCGTTTGTTTGGTCAAACTAGGATGACTTTTGCTTCTATTTGGATTGC
KX853328.1 Onchocerca lupi vou   AACTGATAAGGACCGTTTGTTTGGTCAAACTAGGATGACTTTTGCTTCTATTTGGATTGC
JX080029.1 Onchocerca lupi iso   GACTGATAAGGATCGTTTGTTTGGTCAAACTAGGATGACTTTTGCTTCTATTTGGATTGC
EF521409.1 Onchocerca lupi fro   GACTGATAAGGATCGTTTGTTTGGTCAAACTAGGATGACTTTTGCTTCTATTTGGATTGC
AJ271616.1 Onchocerca gibsoni    AACTGATAAGGATCGTTTGTTTGGTCAGACTAGAATGACTTTTGCTTCTATTTGAATTGC
AJ271617.1 Onchocerca gutturos   GACTGATAAGGACCGTTTGTTTGGTCAAACTAGGATGACTTTTGCTTCTATTTGGATTGC
KP760201.1 Onchocerca gutturos   GACTGATAAGGATCGTTTGTTTGGTCAAACTAGGATGACTTTTGCTTCTATTTGGATTGC
KX853325.1 Onchocerca lienalis   AACTGATAAGGATCGTTTGTTTGGTCAAACTAGGATAACTTTTGCTTCTATTTGAATTGC
KT001213.1 Onchocerca jakutens   AACTGATAAGGATCGTTTGTTTGGTCAGACTAGGATAACTTTTGCTTCTATTTGGATTGC
JQ316672.1 Onchocerca fasciata   AACTGATAAAGACCGTTTATTCGGTCAGACTAGGATGACTTTTGCTTCTATTTGGATTGC
MG188678.1 Onchocerca fasciata   AACTGATAAAGACCGTTTATTCGGTCAGACTAGGATGACTTTTGCTTCTATTTGGATTGC
MT002817.1 Onchocerca fasciata   AACTGATAAAGACCGTTTATTCGGTCAGACTAGGATGACTTTTGCTTCTATTTGGATTGC
```

```
KC167355.1 Onchocerca volvulus  GACTGATAAGGATCGTTTATTTGGTCAGACTAGGATGACTTTTGCTTCTATTTGGATTGC
MH190075.1 Onchocerca volvulus  GACTGATAAGGATCGTTTGTTTGGTCAGACTAGGATGACTTTTGCTTCTATTTGGATTGC
KC167354.1 Onchocerca sp. 'Sii  GACTGATAAGGATCGTTTGTTTGGTCAGACTAGGATGACTTTTGCTTCTATTTGAATTGC
KC167352.1 Onchocerca sp. 'Sii  GACTGATAAGGATCGTTTGTTTGGTCAGACTAGGATGACTTTTGCTTCTATTTGAATTGC
KC167353.1 Onchocerca sp. 'Sii  GACTGATAAGGATCGTTTGTTTGGTCAGACTAGGATGACTTTTGCTTCTATTTGAATTGC
AJ271618.1 Onchocerca ochengi   GACTGATAAGGATCGTTTGTTTGGTCAGACTAGGATAACTTTTGCTTCTATTTGGATTGC
KC167358.1 Onchocerca ochengi   GACTGATAAGGATCGTTTGTTTGGTCAGACTAGGATAACTTTTGCTTCTATTTGGATTGC
KC167351.1 Onchocerca ochengi   GACTGATAAGGATCGTTTGTTTGGTCAGACTAGGATAACTTTTGCTTCTATTTGGATTGC
NC_031891.2 Onchocerca ochengi  GACTGATAAGGATCGTTTGTTTGGTCAGACTAGGATAACTTTTGCTTCTATTTGGATTGC
KC167350.1 Onchocerca ochengi   GACTGATAAGGATCGTTTGTTTGGTCAGACTAGGATAACTTTTGCTTCTATTTGGATTGC
KX181289.2 Onchocerca ochengi   GACTGATAAGGATCGTTTGTTTGGTCAGACTAGGATAACTTTTGCTTCTATTTGGATTGC

                                         610       620       630       640
                                ....|....|....|....|....|....|....|....|....|....
MN564742.1 Dirofilaria sp. 'ho  TGTTTTAGGTACTTCTGTTTGGGGTCATCATATATACACTGCTGGTTTN
MF695085.1 Dirofilaria repens   TGTTTTAGGTACTTCTGTTTGAGGTCATCATATGTATACTGCTGGTTTA
AB518693.1 Onchocerca sp. wild  GGTTTTAGGTACTTCTGTTTGAGGTCACCATATGTACACTGCTGGTTTG
AB518694.1 Onchocerca sp. wild  GGTTTTAGGTACTTCTGTTTGAGGTCACCATATGTACACTGCTGGTTTG
AB518874.1 Onchocerca dewittei  TGTGTTGGGTACTTCTGTTTGGGGTCATCATATGTATACAGCTGGATTG
AB518690.1 Onchocerca dewittei  TGTGTTGGGTACTTCTGTTTGGGGTCATCATATGTATACAGCTGGATTG
AB518692.1 Onchocerca dewittei  TGTGTTGGGTACTTCTGTTTGGGGTCATCATATGTATACAGCTGGATTG
AB518689.1 Onchocerca dewittei  TGTGTTGGGTACTTCTGTTTGGGGTCATCATATGTATACAGCTGGATTG
AM749268.1 Onchocerca eberhard  TGTTTTGGGTACTTCTNNNNNNNNNNNNNNNNNNNNNNNNNNNNNNNNN
LC318284.1 Onchocerca flexuosa  TGTTTTAGGTACTTCTGTTTGAGGTCATCATATGTATACAGCTGGTTTG
AM749269.1 Onchocerca skrjabin  TGTTTTGGGGACTTCTNNNNNNNNNNNNNNNNNNNNNNNNNNNNNNNNN
KX898458.1 Onchocerca boehmi c  TGTTTTGGGGACTTCTGTTTGGGGTCATCATATGTATACTGCTGGTTTG
MG029461.1 Onchocerca sp. ALDA  TGTTTTGGGTACTTCTGTTTGGGGTCATCATATGTATACGGCAGGTTTG
MG029460.1 Onchocerca sp. ALDA  TGTTTTGGGTACTTCTGTTTGGGGTCATCATATGTATACGGCAGGTTTG
MG677940.1 Onchocerca lupi cyt  TGTTTTGGGGACTTCTGTTTGGGGTCACCATATGTATACGGCTGGTTTG
EF521410.1 Onchocerca lupi fro  TGTTTTGGGGACTTCTGTTTGGGGTCACCATATGTATACGGCTGGTTTG
HQ207644.1 Onchocerca lupi fro  TGTTTTGGGGACTTCTGTTTGGGGTCACCATATGTATACGGCTGGTTTG
KC686702.1 Onchocerca lupi cyt  TGTTTTGGGGACTTCTGTTTGGGGTCACCANNNNNNNNNNNNNNNNNNN
AJ415417.1 Onchocerca lupi mit  TGTTTTGGGGACTTCTGTTTGGGGTCACCATATGTATACGGCTGGTTTG
KP283476.1 Onchocerca lupi iso  TGTTTTGGGGACTTCTGTTTGGGGTCACCATATGTATACGGCTGGTTTG
KP283477.1 Onchocerca lupi iso  TGTTTTGGGGACTTCTGTTTGGGGTCACCATATGTATACGGCTGGTTTG
JX080030.1 Onchocerca lupi iso  TGTTTTGGGGACTTCTGTTTGGGGTCACCATATGTATACGGCTGGTTTG
JX183106.1 Onchocerca lupi cyt  TGTTTTGGGGACTTCTGTTTGGGGTCACCATATGTATACGGCTGGTTTG
KX853328.1 Onchocerca lupi vou  TGTTTTGGGGACTTCTGTTTGGGGTCACCATATGTATACGGCTGGTTTA
JX080029.1 Onchocerca lupi iso  TGTTTTGGGGACTTCTGTTTGGGGTCACCATATGTATACGGCTGGTTTG
EF521409.1 Onchocerca lupi fro  TGTTTTGGGGACTTCTGTTTGGGGTCACCATATGTATACGGCTGGTTTG
AJ271616.1 Onchocerca gibsoni   TGTTTTGGGGACGTCTGTGTGGGGTCATCATATGTATACGGCTGGTTTA
AJ271617.1 Onchocerca gutturos  TGTTTTAGGTACGTCTGTTTGGGGTCATCNNNNNNNNNNNNNNNNNNNN
KP760201.1 Onchocerca gutturos  TGTTTTAGGTACGTCTGTTTGGGGCCATCATATATATACTGCTGGTTTG
KX853325.1 Onchocerca lienalis  TGTTCTAGGTACTTCTGTGTGGGGTCATCATATGTATACGGCTGGTTTA
KT001213.1 Onchocerca jakutens  TGTTTTGGGTACTTCTGTGTGAGGTCATCATATATATACGGCTGGTTTG
JQ316672.1 Onchocerca fasciata  TGTTTTAGGGACTTCTGTGTGGGGTCATCATATGTATACAGCTGGTTTG
MG188678.1 Onchocerca fasciata  TGTTTTAGGGACTTCTGTGTGGGGTCATCATATGTATANNNNNNNNNNN
MT002817.1 Onchocerca fasciata  TGTTTTAGGGACTTCTGTGTGGGGTCATCATNNNNNNNNNNNNNNNNNN
KC167355.1 Onchocerca volvulus  TGTTTTAGGTACTTCTGTATGGGGCCATCATATGTATACGGCTGGTTTG
MH190075.1 Onchocerca volvulus  TGTTTTAGGTACTTCTGTATGNNNNNNNNNNNNNNNNNNNNNNNNNNNN
KC167354.1 Onchocerca sp. 'Sii  TGTTTTAGGCACTTCTGTGTGAGGTCATCATATGTATACGGCTGGTTTG
KC167352.1 Onchocerca sp. 'Sii  TGTTTTAGGCACTTCTGTGTGAGGTCATCATATGTATACGGCTGGTTTG
KC167353.1 Onchocerca sp. 'Sii  TGTTTTAGGCACTTCTGTGTGAGGTCATCATATGTATACGGCTGGTTTG
AJ271618.1 Onchocerca ochengi   TGTTTTAGGTACTTCTGTGTGAGGTCATCAATTGTATACGGCNNNNNNN
KC167358.1 Onchocerca ochengi   TGTTTTAGGTACTTCTGTGTGAGGTCATCATATGTATACGGCTGGTTTG
KC167351.1 Onchocerca ochengi   TGTTTTAGGTACTTCTGTGTGAGGTCATCATATGTATACGGCTGGTTTG
NC_031891.2 Onchocerca ochengi  TGTTTTAGGTACTTCTGTGTGAGGTCATCATATGTATACGGCTGGTTTG
KC167350.1 Onchocerca ochengi   TGTTTTAGGTACTTCTGTGTGAGGTCATCATATGTATACGGCTGGTTTG
KX181289.2 Onchocerca ochengi   TGTTTTAGGTACTTCTGTGTGAGGTCATCATATGTATACGGCTGGTTTG
```
